# Cobalt‐Catalyzed Green Alkylations of Anilines with Tetrahydrofurans

**DOI:** 10.1002/cssc.202402622

**Published:** 2025-08-25

**Authors:** Alexandra I. Balalaeva, Zechen Wu, Evgeniya Podyacheva, Oleg I. Afanasyev, Rajenahally V. Jagadeesh, Matthias Beller, Denis Chusov

**Affiliations:** ^1^ A.N. Nesmeyanov Institute of Organoelement compounds of the Russian Academy of Sciences Moscow 119991 Russian Federation; ^2^ Department of Applied Homogeneous Catalysis Leibniz‐Institut für Katalyse e. V. Albert‐Einstein‐Straße 29A 18059 Rostock Germany; ^3^ Faculty of Chemistry National Research University Higher School of Economics Moscow 101000 Russian Federation; ^4^ Nanotechnology Centre Centre for Energy and Environmental Technologies VŠB Technical University of Ostrava Ostrava‐Poruba 708 00 Czech Republic

**Keywords:** alkylation, biomass, renewable resources, syngas, tetrahydrofuran

## Abstract

The use of bio‐renewable resources as starting materials and reagents in synthetic chemistry is an important area for sustainable development. The use of tetrahydrofuran (THF) and 2‐methyltetrahydrofuran (2‐MeTHF) is reported, which can be obtained from lignocellulosic biomass, as potential alkylating agents for anilines. The developed N‐alkylation process is catalyzed by the readily available cobalt salts and employs industrially available syngas as a reducing agent. The reported approach allows for the green and cost‐effective production of N‐alkylanilines from readily available feedstocks.

## Graphical abstract

Is it possible to perform selective alkylation of amines using known green solvents, a simple cobalt salt, and an available commercial gas mixture? It is shown that it is not only possible but also easy to perform selective production of mono‐ and bis‐alkylated amines without alkyl halides using solvents from biomass instead.

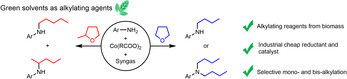

## 1 Introduction

The efficient and environmentally friendly conversion of available resources into value‐added chemicals is a key challenge for modern synthetic chemistry.^[^ ^1^, ^2^, ^3^, ^4^, ^5^ ^]^ Carbohydrates and their derivatives, e.g., polyols, are versatile renewable resources. They are already industrially converted into tetrahydrofuran (THF) derivatives, which are used as solvents, among other applications.^[^ ^6^ ^]^ In this work, we show that anilines can be selectively alkylated with such ethers to yield the corresponding N‐butyl‐ and N‐pentylanilines.

**Scheme 1:**
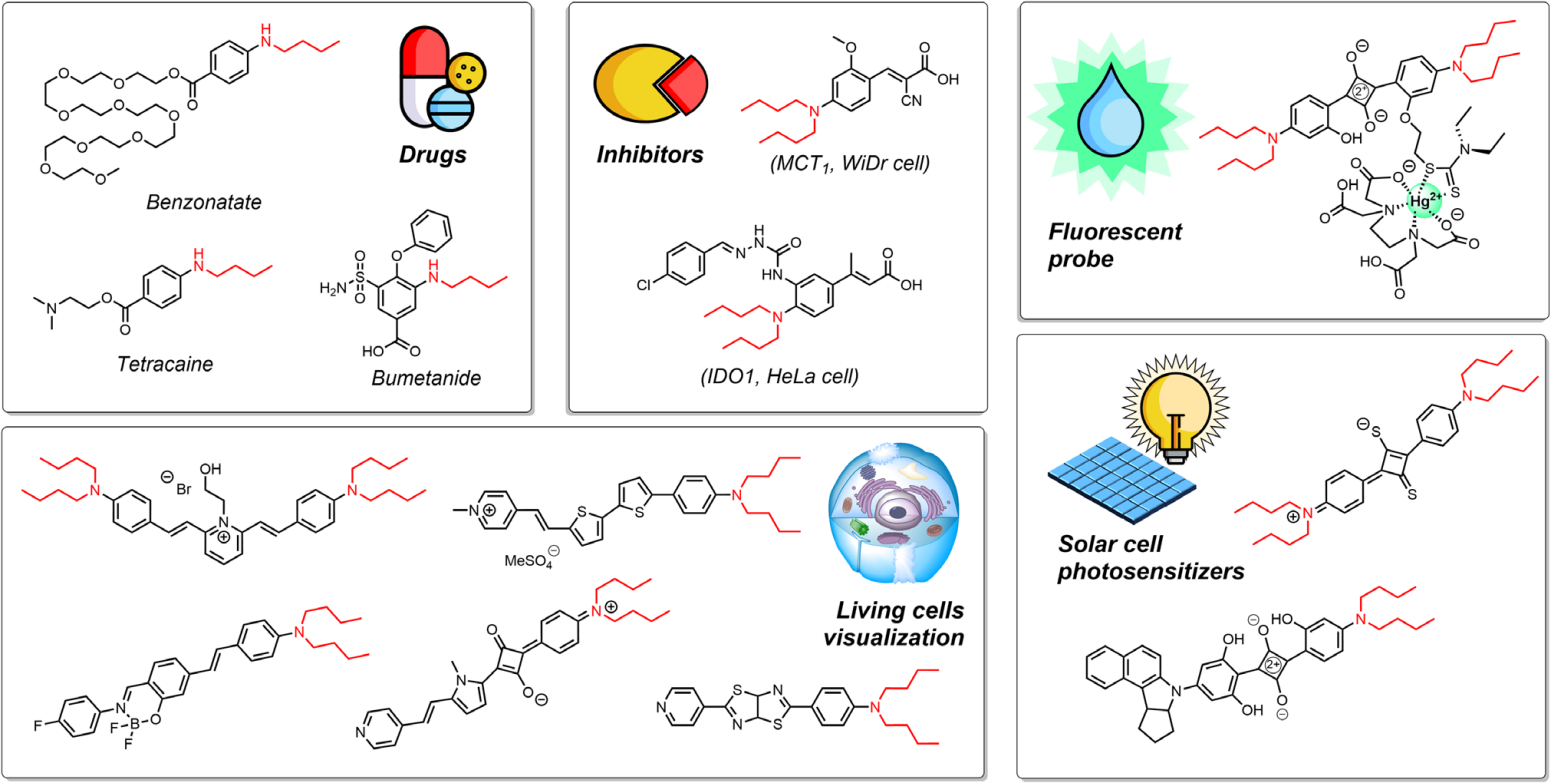
Applications of N‐butylanilines.

N‐alkylated anilines are of interest for a variety of applications in our daily lives (**Scheme** **1**). For example, the butylamine moiety is found in organic light emitting diodes and other photoelectronic materials as well as in photosensitizers for solar cells.^[^ ^7^, ^8^, ^9^ ^]^ Corresponding butylamines are used, among other things, for the visualization of living cells^[^ ^10^ ^]^ and as fluorescent probes.^[^ ^11^ ^]^ In addition, this structural unit is found in a number of currently approved drug molecules such as tetracaine, benzonatate, and bumetanide, and it has been shown that such structures are efficient inhibitors of various enzymes and suppress the proliferation of cell cultures.^[^ ^12^, ^13^, ^14^ ^]^

**Scheme 2:**
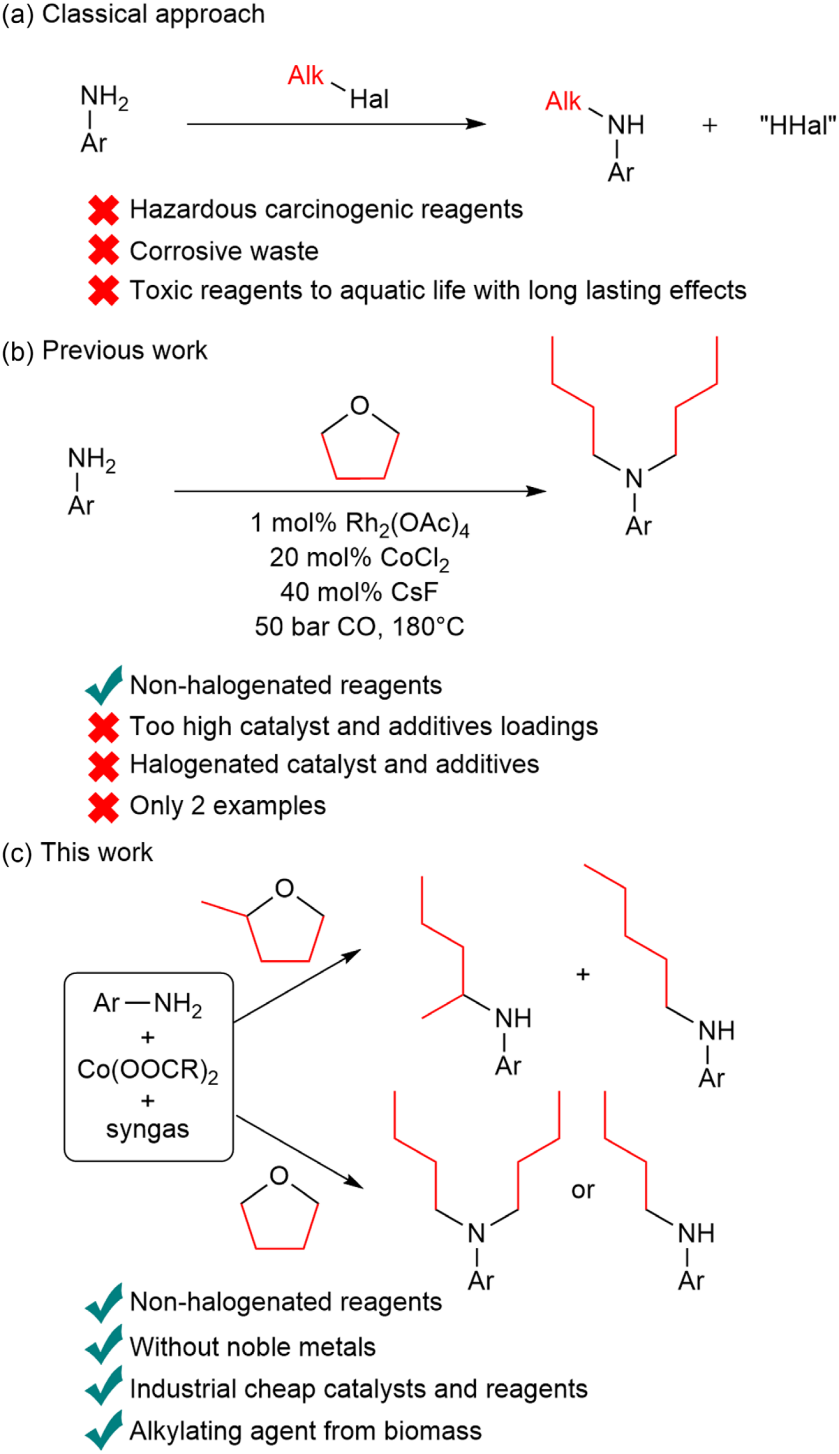
Selected approaches for the N‐alkylation of amines. a) Classical approach. b) Previous work. c) This work.

The traditional synthesis of N‐butylanilines is based on the alkylation of aniline derivatives with alkyl halides.^[^ ^10^, ^14^, ^15^ ^]^ Disadvantages of this method are the comparatively higher cost of the alkyl halide substrates and the generation of equimolar amounts of halogenated waste, which is not in line with the basic principles of green chemistry. In addition, such alkylating agents are potentially carcinogenic and toxic to aquatic life with long‐lasting effects. Another disadvantage is the potential corrosion of the equipment used in the production of alkylating agents (**Scheme** **2**a,b). For these reasons, the development of environmentally friendly approaches for the alkylation of amines is of great importance to both basic research and the chemical industry. In this respect, utilizing THF derivatives as alkylating agents offers interesting opportunities. Recently, we were able to show the feasibility of such transformations in principle.^[^ ^16^ ^]^ For example, anilines react with THF in the presence of 20 mol% CoCl_2_, 40 mol% of CsF, and 1 mol% of Rh_2_(OAc)_4_ and carbon monoxide as a reducing agent to form N‐mono‐ and N,N‐dibutylanilines. However, the high catalyst loading, the need for noble metals, and the need for additional activators are disadvantageous.

In this study, we propose a novel protocol for this N‐alkylation reaction (Scheme 2c). In particular, we demonstrate that anilines can undergo alkylation with THF and 2‐MeTHF using simple cobalt salts. As a beneficial reductant, the available industrial gas mixture, syngas, is used. In agreement with previous literature, the implementation of syngas, as opposed to its constituent elements (i.e., CO and H_2_), enables the execution of amination reactions under more benign conditions and facilitates the utilization of compounds derived from 3d metals in lieu of noble metals, thereby enhancing the efficacy of catalysts.^[^ ^17^, ^18^, ^19^ ^]^

## 2 Results and Discussion

We selected the alkylation of 4‐methoxyaniline with tetrahydrofuran (THF) as a model reaction. Comprehensive optimization specifics are delineated in the ESI. **Table** **1** presents a summary of the relevant results. As indicated by several previous works, cobalt salts without additives have the capacity to catalyze redox processes.^[^ ^20^, ^21^, ^22^, ^23^ ^]^ Nevertheless, it was a surprise to find that as little as 5 mol% of cobalt acetate could catalyze the reductive alkylation of anisidine by THF in the presence of syngas to give **3a**. These conditions are more favorable in comparison to the previously published ones, which involved the use of 1 mol% of Rh_2_(OAc)_4_ and 20 mol% of cobalt compounds (Scheme 2).^[^ ^16^ ^]^ Notably, using Rh_2_(OAc)_4_ in the presence of syngas led to a reduction in the selectivity between mono and dialkylated products (Table 1, entries 1 and 2). Varying the catalyst loading revealed a significant impact on the chemoselectivity of this model reaction. Both a decrease in catalyst loading, as well as an increase, resulted in the prevailing formation of **2a** (Table 1, entries 3 and 6, respectively). Also, a decrease in temperature resulted in a complete shift of the selectivity toward **2a** formation (Table 1, entry 7).

**Table 1:** N‐alkylation of 4‐methoxy anilines with THF: Optimization of the reaction conditions.

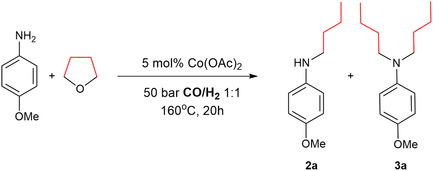			
Entry	Deviation from the stated conditions	2a yield, [%]a)	3a yield, [%]a)
1	None	0	50
2	+1 mol% Rh_2_(OAc)_4_	17	46
3	2 mol% Co(OAc)_2_	37	0
4	33 bar H_2_ + 17 bar CO	2	55
5	150 °C	60	15
6	10 mol% Co(OAc)_2_, 150 °C	70	0
7	10 mol% Co(OAc)_2_, 150 °C, 30 bar H_2_ + 10 bar CO	81	0
8	+1.5 equiv. H_2_O	0	48
9	+10 equiv. H_2_O	51	15
10	Co(acac)_3_	0	55
11	Co(OPiv)_2_	0	57
12	30 bar	1	73
13	Co(OPiv)_2_, 30 bar	1	76

a) NMR or GC yields. Details are given in ESI. Cobalt‐catalyst (2–10 mol%), 0.36 mmol anilines, 12.2–17.6 mmol of THF, 30–50 bar CO:H_2_ (1:1 or 1:3), 150–160 °C, 20 h.

Industrially produced syngas mixtures are distinguished by their respective carbon monoxide‐to‐hydrogen ratios: 1:1 and 1:3. The application of syngas with a 1:3 CO:H_2_ ratio has been demonstrated to suppress the formation of N‐(4‐methoxyphenyl)formamide, a predominant by‐product during mono‐alkylation optimization (ESI, Table S12, Supporting Information). Concurrently, this approach has been shown to enhance the yield of **2a**. Table 1, entry 7 showed the optimal conditions for the mono‐butylation process.

Subsequent optimization revealed that the addition of a minimal amount of water did not affect the reaction outcome (Table 1, entry 8). The application of alternative cobalt sources could potentially reverse the selectivity to the desired product. Cobalt pivalate has been demonstrated to be marginally more efficacious than cobalt acetate (entry 11). In the model reaction, the reduction in pressure facilitated the attainment of a higher yield of **3a** (Table 1, entry 13), attributable to the diminution in the yield of 5‐(butyl(4‐methoxyphenyl)amino)pentan‐1‐ol, the predominant by‐product (ESI, Table S3, Supporting Information). It has been observed that an increase in the H_2_ excess (1:2 CO:H_2_, Table 1, entry 4; Table S9, Supporting Information) results in an increase in the yield of this by‐product to 41% in the reaction mixture. Consequently, the conditions delineated in Table 1, entry 13, are regarded as the optimal conditions for dibutylation. A more thorough presentation of the optimization data, in addition to the screening of all catalysts, can be found in the ESI.

**Scheme 3:**
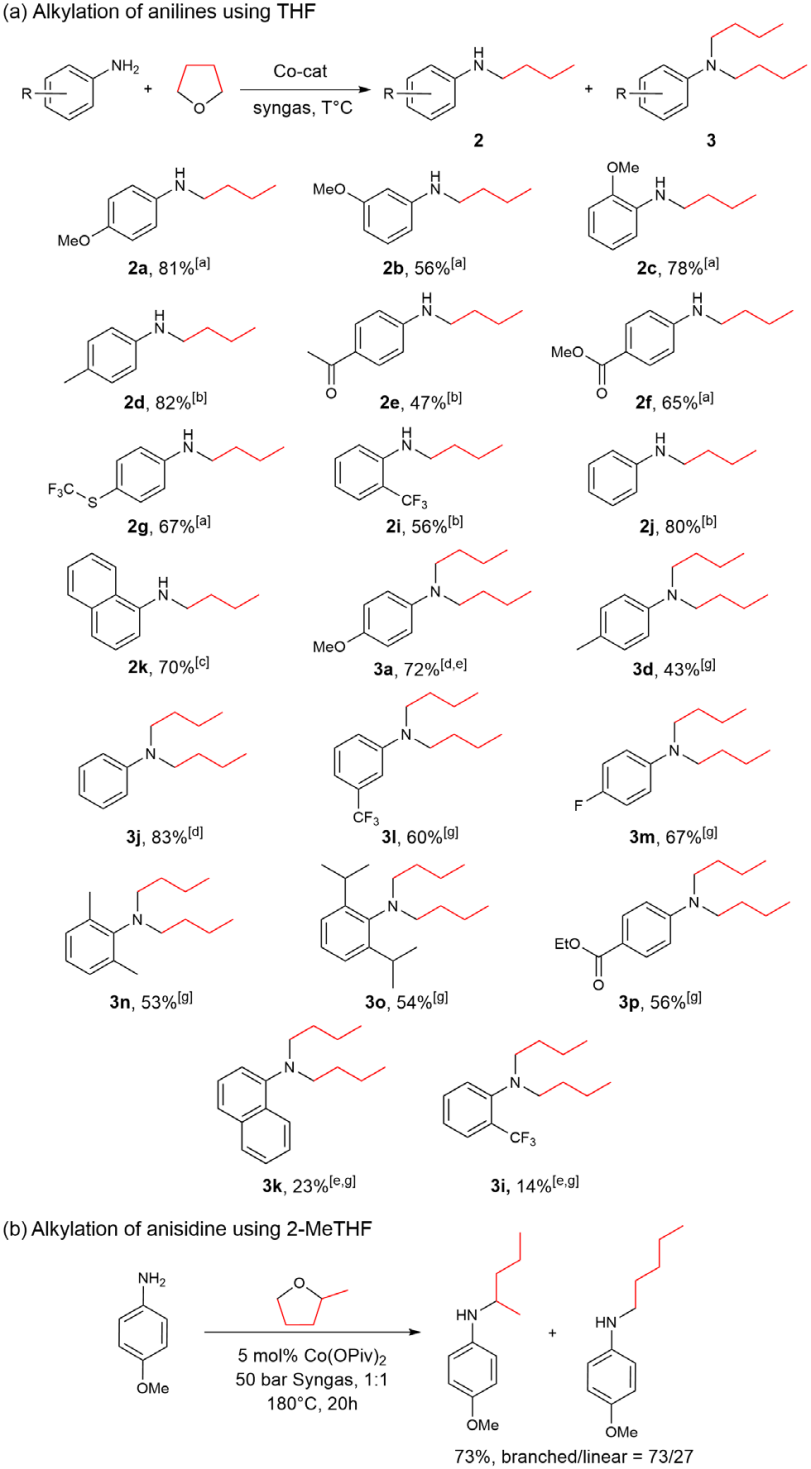
Cobalt‐catalyzed N‐alkylation of anilines with THF a) and 2‐MeTHF b): Substrate scope. [a] 0.36 mmol anilines, 12.2‐17.6 mmol of THF, 10 mol% Co(OAc)_2_, 40 bar CO/H_2_ (1:3), 150 °C; [b] 10 mol% Co(OAc)_2_, 140 °C, 40 bar CO/H_2_ (1:3); [c] 5 mol% Co(OAc)_2_, 150 °C, 40 bar CO/H_2_ (1:3); [d] 5 mol% Co(OPiv)_2_, 160 °C, 30 bar syngas (1:1); [e] NMR yields; [g] 5 mol% Co(OPiv)_2_, 160 °C, 50 bar syngas (1:1).

With these results in hand, we switched to the substrate scope investigation (**Scheme** **3**a). It was shown that developed protocols are applicable to a wide range of aromatic amines. Mono and dialkylation suitably works on substrates with electron‐donating and electron‐withdrawing substituents, for example, the ester moiety in the para position (**2f**, **3p**) does not inhibit the reaction. The different functional groups, like trifluoromethylthio (**2 g**), trifluoromethyl (**2i, 3l**), and even keto (**2e**) were tolerated. Noteworthy, **2f** is a direct precursor of the local anesthetic, tetracaine, and it was obtained up to 65% yield.^[^ ^24^ ^]^ Ortho‐disubstituted sterically hindered anilines could be easily dialkylated to give **3n** and **3o**. However, *o*‐trifluoromethylaniline was monoalkylated to provide the corresponding product in a good yield (**2i**), whereas it gave only a low yield under the dialkylation conditions (**3i**). This might be related to the combination of nucleophilicity of the nitrogen atom, together with the steric effect. For the ortho‐disubstituted anilines nucleophilicity of the nitrogen atom is still high, and the sterically hindered product can be synthesized with an appropriate yield (**3n**, **3o**). While the electron‐withdrawing effect of ortho‐trifluoromethyl and alpha‐naphthyl groups leads to the lower yield (**2k** vs. **3k**, **2i** vs. **3i**). It should be noted that the synthesis of dialkylated aniline derivatives other than the model 4‐methoxy‐dibutyl aniline necessitates an additional tuning of the reaction conditions. In order to achieve a higher level of selectivity with regard to di‐/mono‐alkylated products, it is necessary to increase the pressure of syngas 1:1 from 30 bar to 50 bar.

**Scheme 4:**
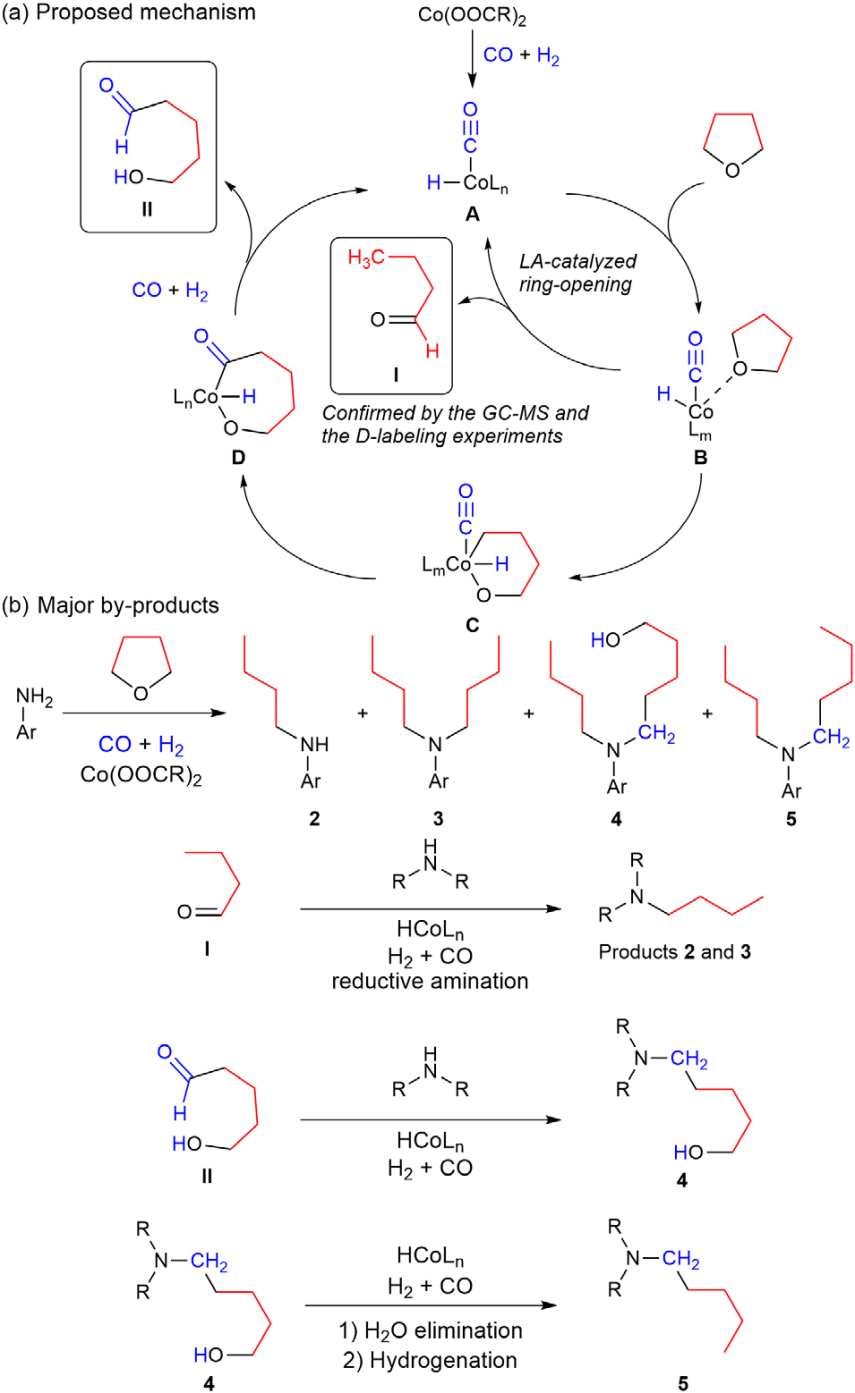
Proposed mechanism for the alkylation of anilines by THF and possible ways of by‐product formation.

To demonstrate the application of this approach in biomass conversion, we further tested the alkylation of 4‐methoxyaniline with 2‐methyltetrahydrofuran (Scheme 3b). In this case, the formation of a mixture of linear and branched products in a good yield occurred. Higher selectivity branched/linear products were observed at higher temperature (ESI, Table S13, Supporting Information). This could be explained by the prevailing formation of a thermodynamically more stable ketone intermediate compared to a kinetic aldehyde intermediate, leading to a linear product (vide infra **Scheme** **4**).

Based on several control experiments, we propose the following mechanism for this N‐alkylation reaction (Scheme 4, ESI). We suppose that the key intermediate in this process is butanal, which is formed from THF. This was confirmed by gas chromatography mass spectrometry analysis (ESI, Figure S1, Supporting Information). Moreover, in an additional control experiment in the presence of CO/H_2_ with a 49/1 ratio, we detected the formation of quinoline **12a** (Table S24, Supporting Information). This latter product could be formed via in situ generation of butanal, its self‐condensation to 2‐methylheptanal, followed by a Michael addition of 4‐methoxyaniline and condensation (ESI, Scheme S1, Supporting Information).

Next, we checked the distribution of deuterium atoms in the product using THF‐d_8_ as an alkylating agent (ESI, Scheme S3, Supporting Information). In this case, we detected one hydrogen atom in the α‐position to nitrogen and two hydrogens in the β‐position. This indicates that butanal‐d_8_ undergoes hydrogen exchange via tautomeric equilibrium, followed by the classical reductive amination.

Regarding the active catalyst species, we propose a mixture of cobalt hydride species, which are formed from cobalt salt and syngas in the presence of the amine. Anilines are known to coordinate with cobalt species. However, these complexes were too unstable to be isolated and characterized. The importance of the amine was also demonstrated in an experiment in which we used a tenfold decrease in aniline (see ESI). In this experiment, the amine‐to‐cobalt ratio was 2:1, resulting in the observation of a proportional amount of dibutyl‐anisidine within the complex product mixture (Scheme 4a).^[^ ^25^, ^26^, ^27^ ^]^

To prove the nature of the active catalyst, we conducted a mercury test experiment (see ESI). Interestingly, the reaction selectivity changed in the presence of metallic Hg, though the conversion remained the same. Therefore, we conclude that several active catalyst species, including homogeneous complexes and nanoparticles, might exist. The components of syngas (CO and H_2_) are beneficial for the reductive process. While both can serve as reducing agents, CO can also stabilize metal‐hydride particles as a ligand.^[^ ^17^ ^]^

As shown in Scheme 4, the catalytic cycle starts with the formation of carbonyl‐hydride cobalt species (**A**). In this case, L represents any coordinating molecule in the system, such as an amine, solvent, or CO. THF then coordinates to the Co center, forming species **B**, followed by THF ring opening to furnish butanal (intermediate **I**). In this case, the cobalt‐catalyst is likely to act as a simple Lewis acid.

As major by‐products of the model reaction, aminoalcohol **4** and N,N‐butylpentylamine **5** were observed (see Scheme 4b). The formation of these by‐products led us to propose a way in which they form via cobalt‐catalysis. It is known that cobalt salts can form cyclic compounds with various bifunctional substances containing a cobalt atom in the cycle in the presence of CO/H_2_.^[^ ^28^ ^]^ The formation of species **C**, followed by the insertion of CO into the skeleton to give complex **D**, is likely to furnish the β‐hydroxyaldehyde II via β‐hydride elimination.^[^ ^29^, ^30^ ^]^

Next, we assume the following processes. Butanal (**I**) reacts with amine via the classical reductive amination pathway to give products **2** and **3**. Reductive amination of 4‐methoxyaniline with **II** leads to the product **4**.^[^ ^31^ ^]^ Finally, **4** could be dehydrated and hydrogenated to give product **5**.

## 3 Conclusions

In conclusion, we have developed a useful protocol for the selective alkylation of anilines with THF to produce monoalkylated and dialkylated products. This protocol does not necessitate the use of halogenated reagents and is ecologically sustainable. The starting materials and reagents utilized in this process are all readily available and inexpensive, with some being produced on a multiton scale. The use of simple cobalt salts as catalysts was a key element of the study. As an example of the utility of the developed protocol, the direct precursor of the local anesthetic, tetracaine, was prepared under the developed conditions. All these facts render this protocol suitable for a green synthesis of high‐value products.

## Supporting Information

All data supporting these results are available in ESI: Optimization details, compounds characterization data and copies of NMR spectra. The authors have cited additional references within the Supporting Information.^[^ ^32^, ^33^, ^34^, ^35^, ^36^, ^37^, ^38^, ^39^, ^40^, ^41^, ^42^, ^43^, ^44^, ^45^, ^46^ ^]^

## Conflict of Interest

The authors declare no conflict of interest.

## Supporting information

Supplementary Material

## Data Availability

The data that support the findings of this study are available in the supplementary material of this article.
