# Supplementary material for: Cobalt‐Catalyzed Green Alkylations of Anilines with Tetrahydrofurans
Source: ChemSusChem. 2025 Aug 25;18(19):e202402622. doi: 10.1002/cssc.202402622 (PMC12487734; doi:10.1002/cssc.202402622)

Cobalt-catalyzed Green Alkylations of Anilines with Tetrahydrofuranes

Alexandra I. Balalaeva,+ [a] Zechen Wu,+ [c] Evgeniya Podyacheva,+ [a, b] Oleg I. Afanasyev, [a] Rajenahally V. Jagadeesh, [c, d] * Matthias Beller, [c] * and Denis Chusov [a, b] *

+ These authors contributed equally

[a] A.I. Balalaeva, E. Podyacheva, O.I. Afanasyev, Dr. D. Chusov
A.N. Nesmeyanov Institute of Organoelement compounds of the Russian Academy of Sciences
Moscow 119991 (Russian Federation)
E-mail: denis.chusov@gmail.com

[b] E. Podyacheva, Dr. D. Chusov
National Research University Higher School of Economics
Moscow 101000 (Russian Federation)

[c] Z. Wu, Prof. Dr. R.V. Jagadeesh, Prof. Dr. M. Beller
Department of Applied Homogeneous Catalysis
Leibniz-Institut für Katalyse e. V.,
Albert-Einstein-Straße 29A, 18059 Rostock (Germany)
E-mail: *jagadeesh.rajenahally@catalysis.de*
matthias.beller@catalysis.de

[d] Prof. Dr. R.V. Jagadeesh
Nanotechnology Centre, Centre of Energy and Environmental Technologies, VŠB Technical University of Ostrava
Ostrava-Poruba (Czech Republic)

Supporting Information

Table of contents

1. General information ... 2
2. Experimental section ... 4
   2.1 Optimization of reaction conditions ... 4
   2.2 Control experiments ... 13
   2.3 Failed substrates ... 21
   2.4 Spectroscopic and analytical data ... 24
3. References ... 31
4. ^{1}H, ^{13}C, ^{19}F NMR and HRMS spectra of obtained compounds ... 32

1. General information

Unless otherwise stated, all reagents, were purchased from commercial suppliers and used without further purification. THF was obtained from MBRAUN Solvent Purification System MB-SPS-7, without stabilizer.

Isolation of products was performed using column chromatography (Macherey-Nagel, silica gel, particle size 0.04-0.063 mm), using preparative flash chromatograph InterChim PuriFlash; or using preparative thin-layer chromatography (Macherey-Nagel, silica gel N/UV254, particle size 0.002-0.020 mm). All details about particular chromatographic parameters are provided with the description of each compound.

^{1}H, ^{13}C{^{1}H}, and ^{19}F spectra were recorded in $CDCl_3$ on Bruker Avance 300, Bruker Avance 400, or Varian Inova 400 spectrometers. Chemical shifts are reported in parts per million relative to $CHCl_3$ (7.26 and 77.16 ppm for ^{1}H and ^{13}C{^{1}H} respectively). The following abbreviations were used to designate chemical shift multiplicities: s = singlet, d = doublet, dd = doublet of doublets, t = triplet, q = quartet, quint. = quintet, m = multiplet, br = broad, sept = septet; quint. = quintet, h = heptet coupling constants are given in Hertz (Hz).

High-resolution mass spectra were recorded on a LCMS-9030 device (Shimadzu, Japan) by electrospray ionization mass spectrometry (ESI-MS). Measurements were carried out in positive ion mode; samples were dissolved in acetonitrile and injected into the mass-spectrometer chamber from an HPLC system LC-40 Nexera (Shimadzu, Japan). The following parameters were used: capillary voltage 4.0 kV; mass scanning range: m/z 100–2000; external calibration with solution NaI in MeOH/H_2O; drying and heating gases (nitrogen) (each 10 L/min); nebulizing gas (nitrogen) (3 L/min); interface temperature: 300℃; flow rate acetonitrile/H_2O (95/5) 0.4 mL/min. Molecular ions in the spectra were analyzed and matched with the appropriately calculated *m*/*z* and isotopic

profiles in the LabSolutions v.5.114 program. Predicted mass-spectra were obtained using mMas software (for Windows version 5.5.0).[1]

Analytical gas chromatography (GC) was performed using a Chromatec Crystal 5000.2 gas chromatograph fitted with a flame ionization detector (helium was used as the carrier gas, 37 mL/min) and a MS detector. Chromatec CR-5MS (30 meters) capillary column was used.

GC settings for the yield determination using FID detector and CR-5MS column:

The injector temperature was 250°C, split ratio of 10:1 at the moment of injection, the FID temperature was 250°C. Column compartment temperature program: 100°C for 2 min, 100°C → 290°C at 30°C/min, 290°C for 3 min. Flow rate 1.5 mL/min, column CR-5MS. Retention times (t_R) and integrated ratios were obtained using Chromatec Analytic Software.

GC settings for the qualitative analysis using MS detector and CR-5MS column:

The injector temperature was 250°C, split ratio of 40:1 at the moment of injection. Column compartment temperature program: 60°C for 4 min, 60°C → 250°C at 30°C/min, 250°C for 12 min. Flow rate 1 mL/min. MSD parameters: ion source temperature 200°C, transfer line temperature 290°C.

Analysis of the gas mixtures was performed using Chromatec Crystal 5000.2 gas chromatograph fitted with the thermal conductivity detector. Argon was used as a career gas (15 ml/min flow rate). Column zeolite CaX, 0.25-0.5 mm, 2 m x 3 mm. Isotermic mode, t = 60°C. H_2 retention time is 1.42 min, CO retention time is 4.84 min. A gas mixture to be analyzed (from the gas cylinder or an autoclave) was diluted in argon to achieve a concentration in the range 1-5 % v/v, and this diluted gas mixture was injected into GC. Quantities of the gases were determined using external calibration.

Reactions with pressure were carried out in autoclaves made from stainless steel and titanium.

Syngas preparation: to a gas cylinder with ca. 25 bar of carbon monoxide ca. 25 bar of hydrogen were added (to achieve a total pressure of 50 bar). The cylinder was equilibrated at room temperature for 24 hours, and the resulting gas mixture was analyzed by GC. The gas was used if H_2:CO ratio was 1-1.3:1. The analysis was repeated periodically, if the ratio was outside the above-mentioned range, it was adjusted by addition of the corresponding gas followed by equilibration and GC analysis.

2. Experimental section

2.1 Optimization of reaction conditions

Catalysts preparation

Cobalt (II) pivalate was synthesized according to published procedure[2]:

19 g (0.23 mol) of sodium bicarbonate was dissolved in 160 mL of distilled water, heated on water bath to 50℃. 12 g (0.05 mol) of cobalt (II) chloride hexahydrate was dissolved in 50 mL of distilled water and then this solution was added dropwise to the preheated solution of sodium bicarbonate for 2 h at 50℃. After adding the cobalt (II) chloride, resulting solution was stirred at 50℃ for another 1 hour. The obtained purple precipitate was filtered on a Schott filter and washed with distilled water preheated to 70℃. The cobalt (II) carbonate was dried at a temperature of 60℃ resulting in 5.27 g of fine purple-pink precipitate.

Cobalt carbonate (4.0 g, 34 mmol) was treated with an excess of pivalic acid (20.0 g, 196 mmol) in the presence of water (3 mL) at 100℃ for 24 h, leading to dissolution of the carbonate salt. The solution was cooled to room temperature, MeCN (50 mL) was added and the mixture was stirred for short time. The solution was filtered and cooled to 5℃, giving pink crystals of **1**. The solution was then cooled to -18℃ for 2 days to give a second crop of **1**. The second crop was collected by filtration, washed with cold MeCN and dried in a slow flow of Ar. Complex **1** has a high solubility at room temperature in a wide range of organic solvents (from MeCN to pentane) giving a violet unstable solution. Complex **1** dissolves in H_2O to give a pink solution. Yield: 65.8%, 10.5 g.

1 = $[Co(OPiv)_2(PivOH)]*0.5H_2O$

Elemental analysis: Found, %: Co, 13.05; C, 50.24; H, 7.88. Calculated, %: Co, 12.42; C, 50.63; H, 8.29.

Compound **1** (3.0 g, 3.2 mmol) was heated to 180℃ under a flow of nitrogen for 15 h, during which time the sample changed color from pink to violet, and decomposed to give a microcrystalline solid **2**. Yield: 99.3%, 1.64g.

2 = $[Co(OPiv)_2]_n$

Elemental analysis: Found, %: Co, 22.60; C, 45.81; H, 7.05. Calculated, %: Co, 22.56; C, 45.99; H, 6.95.

Anhydrous cobalt (II) acetate was obtained by refluxing $Co(OAc)_2*4H_2O$ in acetic acid anhydride for 5 h. The resulting mixture was dried in vacuo.

Elemental analysis: Found, %: C, 26.8; H, 3.55. Calculated, %: C, 27.14; H, 3.42.

Cobalt (II) tetrahydrate was used from commercial source.

Elemental analysis: Found, %: Co, 24.10; C, 21.22; H, 5.47. Calculated, %: Co, 23.66; C, 19.29; H, 5.67.

Cobalt (III) acetylacetonate was synthesized according to published procedure[3]:

3.166 g of $CoCO_3*xH_2O$ (0.025 mol) were dissolved in 24 ml of acetylacetone at 90℃ in an oil bath. 36 ml of 10% H_2O_2 solution was added dropwise during an hour. Three-

neck flask was cooled down in an ice bath. Dark green crystals were filtered, precipitate was dried at 110℃ for 3 hours. The dried precipitate was dissolved in 50 ml of boiling benzene, 170 ml of hexane was added to the warm solution, the resulting mixture was cooled in an ice bath. The green precipitate was filtered using a Schott filter and dry on air. Yield: 83%, 7.41 g.

Elemental analysis: Found: C, 50.45%, H, 5.82%. Calculated, %: C, 50.57%, H, 5.94%.

General procedure 1.

$Co(OAc)_2$ (18–36 μmol, 5–10 mol%), amine (0.36 mmol, 100 mol%), magnetic stirrer were charged into a glass vial in 4 ml stainless steel autoclave. 1.4 ml THF (17.6 mmol, 49 equiv.) was added and autoclave was sealed, flushed six times with nitrogen, six times with hydrogen, three times with carbon monoxide, and then charged with 10 bar of carbon monoxide and 30 bar of hydrogen (to achieve a total pressure of 40 bar). The autoclave was placed into a preheated to 140-150℃ passive metal thermostat, stirring velocity corresponded 500 rpm. After 20 h of heating and stirring the autoclave was cooled to room temperature and depressurized. The reaction mixture was diluted with ethyl acetate and concentrated in vacuo. The crude product was purified by silica gel column chromatography with pentane/ethyl acetate to afford the corresponding product. The yield of the product was determined by weight.

General procedure 2.

Amine (0.36 mmol, 100 mol%), magnetic stirrer, 1 mL THF (12.1 mmol, 34 equiv.) were charged into a glass vial in 10 ml stainless steel autoclave. Aliquot of $Co(OPiv)_2$ (18 μmol, 5 mol%) (stock solution in THF, c=10 mg/mL), was added and autoclave was sealed, flushed three times with 5 bar of Syngas, and then charged with 50 bar of Syngas (CO/H_2 = 1/1). The autoclave was placed into a preheated to 160℃ oil bath, stirring velocity corresponded 380 rpm. After 20 h of heating and stirring the autoclave was cooled to room temperature and depressurized. The reaction mixture was transferred in 5 mL graduated vial with CH_2Cl_2. The reaction filtrated through celite layer, resulting solution was used for further analysis. The yield of the product was determined by GC-FID calibration or ^{1}H NMR.

Optimization for N-alkylation of 4-methoxyaniline with THF

Table S1. Catalyst loading.

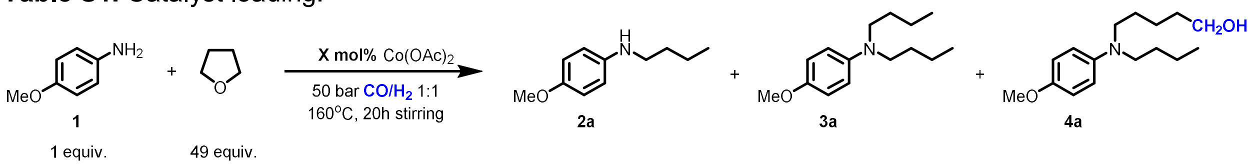

X mol%	Yield of **2a**, %	Yield of **3a**, %	Yield of **4a**, %
1	21	0	0
2	37	0	0
3	33	20	13
4	5	37	28
5*	0	50	30
10	0	51	35
15	0	56	29
20	0	53	34

3.6–72 μmol of $Co(OAc)_2$, 0.36 mmol of 4-methoxyaniline, 17.6 mmol of THF, 160°C, 50 bar of Syngas, 20 h, stirring velocity is 380 rpm.
The average yields for 2 experiments are presented. Yields were determined by GC-FID calibration.
*Average yields for 17 experiments.

Table S2. Temperature variation.

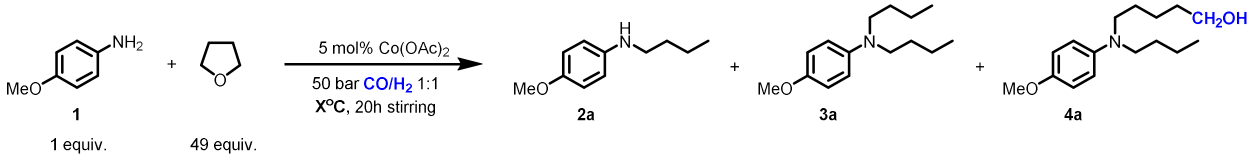

T, °C	Yield of **2a**, %	Yield of **3a**, %	Yield of **4a**, %
180	28	39	25
160*	0	50	30
150	60	15	20
140	53	0	0
120	0	0	0

18 μmol of $Co(OAc)_2$, 0.36 mmol of 4-methoxyaniline, 17.6 mmol of THF, 120–180°C, 50 bar of Syngas, 20 h, stirring velocity is 380 rpm.
The average yields for 2 experiments are presented. Yields were determined by ^{1}H NMR.
*Average yields for 17 experiments.

Table S3. Pressure variation.

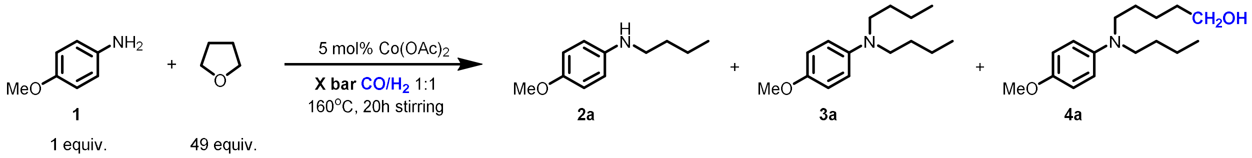

p, bar	Yield of **2a**, %	Yield of **3a**, %	Yield of **4a**, %
70	1	37	34
60	2	44	33
50*	0	50	30
40	0	65	29
30	1	73	24
20	11	0	0
10	4	0	0

18 μmol of $Co(OAc)_2$, 0.36 mmol of 4-methoxyaniline, 17.6 mmol of THF, 160°C, 10-70 bar of Syngas, 20 h, stirring velocity is 380 rpm.

The average yields for 2 experiments are presented. Yields were determined by GC-FID calibration.
*Average yields for 17 experiments.

Table S4. Time variation.

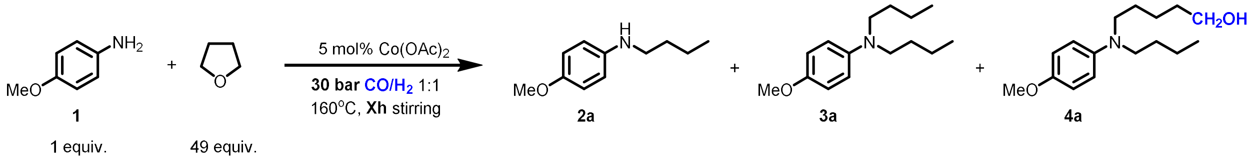

X h	Yield of **2a**, %	Yield of **3a**, %	Yield of **4a**, %
3	38	traces	0
20*	traces	76	24
72	traces	78	18

18 µmol of $Co(OAc)_2$, 0.36 mmol of 4-methoxyaniline, 3.6–21.6 mmol of THF, 160°C,
30 bar of Syngas, X h, stirring velocity is 380 rpm.
The average yields for 2 experiments are presented. Yields were determined by GC-FID calibration.
*Average yields for 3 experiments.

Table S5. Ratio between 4-methoxyaniline and THF influence.

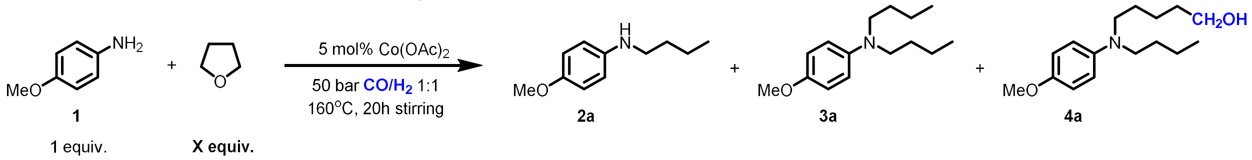

X equiv.	Yield of **2a**, %	Yield of **3a**, %	Yield of **4a**, %
60	0	60	34
49*	0	50	30
30	43	9	0
20	39	2	0
10	20	0	0

18 µmol of $Co(OAc)_2$, 0.36 mmol of 4-methoxyaniline, 3.6–21.6 mmol of THF, 160°C,
50 bar of Syngas, 20 h, stirring velocity is 380 rpm.
The average yields for 2 experiments are presented. Yields were determined by GC-FID calibration.
*Average yields for 17 experiments.

Table S6. Additives influence. [Co] = $Co(OAc)_2$

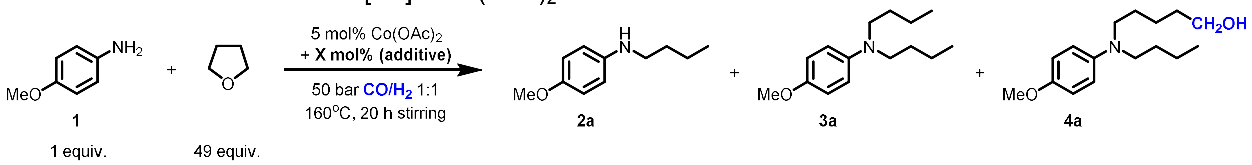

Additive	X mol%	Yield of **2a**, %	Yield of **3a**, %	Yield of **4a**, %
-*	-	0	50	30
$Rh_2(OAc)_4$	0.1	0	49	29
	1	17	46	14
H_2O	150	0	48	32
	1000	51	15	13
	2000	21	0	0
Mol. sieve (3Å)**		37	3	0
toluene	2000	38	19	16
	4000	53	10	10
LiCl	3	35	1	0
Ph_3P	5	30	1	0

18 µmol of $Co(OAc)_2$, 0.36 mmol of 4-methoxyaniline, 17.6 mmol of THF, 160°C,
50 bar of Syngas, 20 h, stirring velocity is 380 rpm.

The average yields for 2 experiments are presented. Yields were determined by GC-FID calibration.
*Average yields for 17 experiments.
**3.8 g (mol. sieve) per 1 g (4-methoxyaniline).

Table S7. Additives influence. [Co] = Co(acac)$_3$.

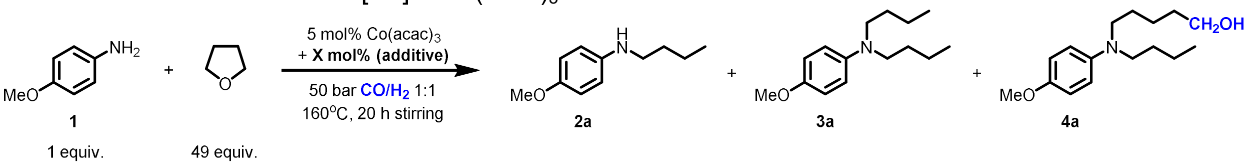

Additive	X mol%	Yield of **2a**, %	Yield of **3a**, %	Yield of **4a**, %
-	-	-	55	29
LiCl	0.5	11	34	30
	1	14	28	22
	2	34	4	17
	5	26	0	0
	10	9	0	0
LiF	5	21	24	22
Ph_3P	0.5	9	30	25
	1	13	25	20
	2	38	8	8
TEBAC	5	0	0	0
TBAF*3H_2O	5	4	0	0
Ru(acac)$_3$	1	0	53	26

18 μmol of Co(acac)$_3$, 0.36 mmol of 4-methoxyaniline, 17.6 mmol of THF, 160°C, 50 bar of Syngas, 20 h, stirring velocity is 380 rpm.
The average yields for 2 experiments are presented. Yields were determined by GC-FID calibration.

Table S8. Catalyst screening. [Ru].

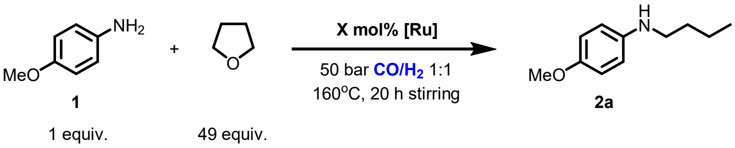

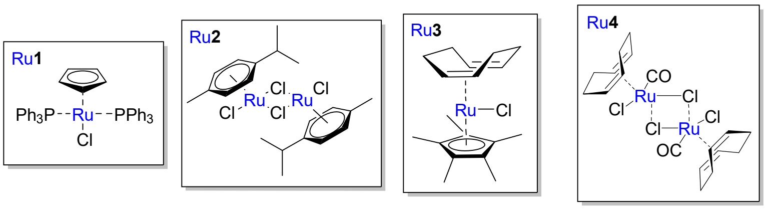

Catalyst	X mol%	Yield of **2a**, %
Ru(acac)$_3$	3	43
$RuCl_3$	3	0
Ru**1**	3	0
Ru**2**	7	0
Ru**3**	5	0
Ru**4**	3	0

10-27 μmol of [Ru], 0.36 mmol of 4-methoxyaniline, 17.6 mmol of THF, 160°C, 50 bar of Syngas, 20 h, stirring velocity is 380 rpm.
The average yields for 2 experiments are presented. Yields were determined by GC-FID calibration.

Table S9. Syngas composition variation.

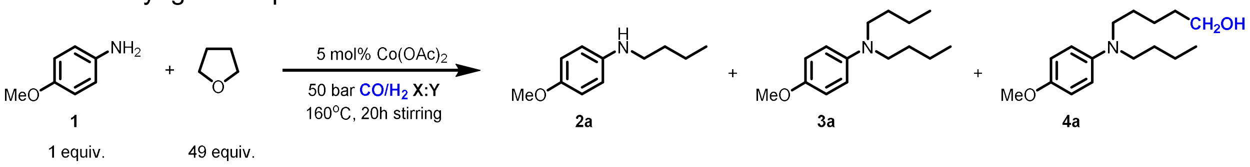

CO/H_2	Yield of **2a**, %	Yield of **3a**, %	Yield of **4a**, %
49/1	35	0	0
3/1	40	28	15
1/1*	0	50	30
1/2	3	55	41
1/4	20	0	0
1/49	0	0	0

18 µmol of $Co(OAc)_2$, 0.36 mmol of 4-methoxyaniline, 17.6 mmol of THF, 160°C,
50 bar of Syngas (CO/H_2 from 49/1 to 1/49), 20 h, stirring velocity is 380 rpm.
The average yields for 2 experiments are presented. Yields were determined by GC-FID calibration.
*Average yields for 17 experiments.

Table S10. Catalyst screening. [Co], 50 bar.

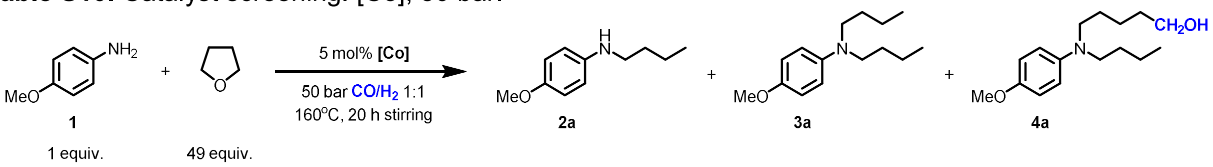

Catalyst	Yield of **2a**, %	Yield of **3a**, %	Yield of **4a**, %
$Co(OAc)_2$*	0	50	30
$Co_2(CO)_8$	34	31	0
$Co(acac)_2$	36	7	0
$Co(acac)_3$	0	55	29
$[Co(OPiv)_2(PivOH)]*0.5H_2O$	0	56	34
$Co(OPiv)_2$	0	57	36

18 µmol of [Co], 0.36 mmol of 4-methoxyaniline, 17.6 mmol of THF, 160°C,
50 bar of Syngas, 20 h, stirring velocity is 380 rpm.
The average yields for 2 experiments are presented. Yields were determined by GC-FID calibration.
*Average yields for 17 experiments.

Table S11. Catalyst screening. [Co], 30 bar.

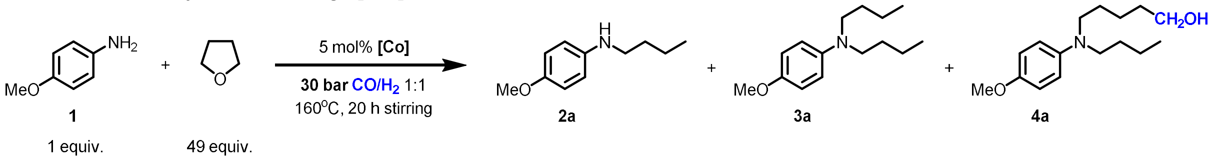

Catalyst	Yield of **2a**, %	Yield of **3a**, %	Yield of **4a**, %
$Co(OAc)_2$	2	72	26
$Co(OTf)_2$	48	11	1
$[Co(OPiv)_2(PivOH)]*0.5H_2O$	7	67	24
$Co(OPiv)_2$	1	76	19

18 µmol of [Co], 0.36 mmol of 4-methoxyaniline, 17.6 mmol of THF, 160°C,
30 bar of Syngas, 20 h, stirring velocity is 380 rpm.
The average yields for 2 experiments are presented. Yields were determined by GC-FID calibration.

Table S12. Optimization for anilines mono-alkylation.

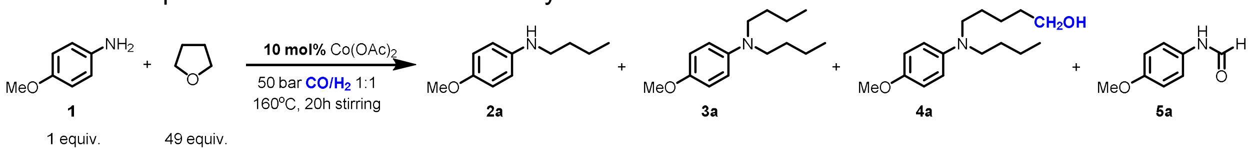

Deviation from the standard conditions	Conversion of **1**, %	Yield of **2a**, %	Yield of **3a**, %	Yield of **4a**, %	Yield of **5a**, %
none*	99	10	56	23	6
[Co] = $Co(NO_3)_2$*$6H_2O$	21	0	0	0	0
[Co] = $Co(acac)_2$	70	51	trace	trace	14
[Co] = $Co(OTf)_2$	18	trace	trace	trace	trace
[Co] = $CoCl_2$*$6H_2O$	23	trace	trace	trace	trace
[Co] = $Co(BF_4)_2$*$6H_2O$	80	57	trace	trace	14
150℃	99	71	trace	trace	22
150℃ and CO/H_2 1:3, 40 bar	99	81	trace	trace	trace

18-36 µmol of [Co], 0.36 mmol of 4-methoxyaniline, 17.6 mmol of THF, 160°C, 40-50 bar of Syngas (CO/H_2 1/1, 1/3), 20 h, stirring velocity is 500 rpm. The average yields for 3 experiments are presented. Isolated yields.
*Average yields for 5 experiments.

Optimization for N-alkylation with 2-Me-THF

Table S13. Influence of catalyst loading and temperature. [Co] = $Co(OAc)_2$.

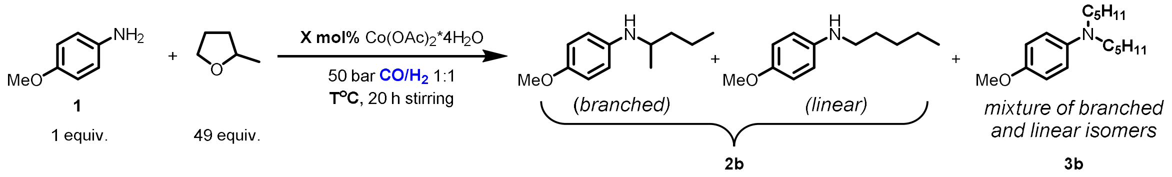

T, °C	X mol%	Yield of **2b**, %	*branched/linear*	Yield of **3b**, %
160	10	52	85/15	0
	20	21	99/1	7
180	10	56	93/7	4

36-72 µmol of $Co(OAc)_2$*$4H_2O$, 0.36 mmol of 4-methoxyaniline, 17.6 mmol of 2-Me-THF, 160-180°C, 50 bar of Syngas, 20 h, stirring velocity is 380 rpm.
The average yields for 2 experiments are presented. Yields were determined by GC-FID calibration.

Table S14. Catalyst screening. [Co].

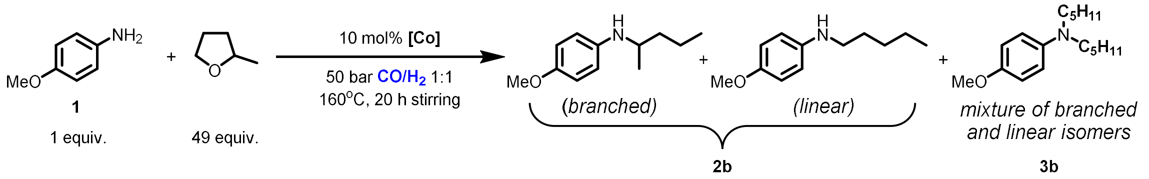

Catalyst	Yield of **2b**, %	*branched/linear*	Yield of **3b**, %
$Co(OAc)_2$*$4H_2O$	52	85/15	0
$Co(OPiv)_2$	55	82/18	traces
$Co(OTf)_2$	55	76/24	2
$Co(BF_4)_2$*$6H_2O$	48	71/29	5
Na_2(Co-EDTA)	0	-	0

36 µmol of [Co], 0.36 mmol of 4-methoxyaniline, 17.6 mmol of 2-Me-THF, 160°C, 50 bar of Syngas, 20 h, stirring velocity is 380 rpm.
The average yields for 2 experiments are presented. Yields were determined by GC-FID calibration.

Table S15. Influence of catalyst loading and time. [Co] = $Co(OPiv)_2$.

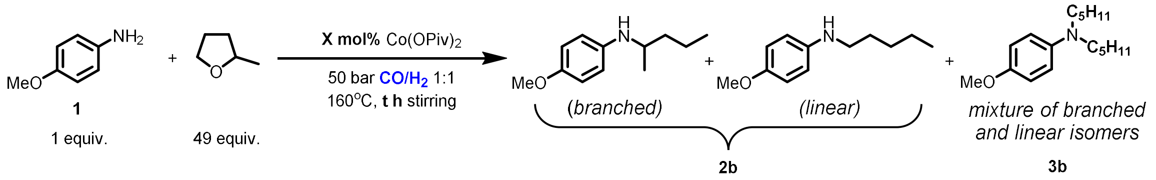

X mol%	t, h	Yield of **2b**, %	*branched/linear*	Yield of **3b**, %
2	44	56	73/27	traces
5	20	53*	75/25	traces
	44	48	85/15	4
10	20	55	82/18	traces

7.2-36 µmol of [Co], 0.36 mmol of 4-methoxyaniline, 17.6 mmol of 2-Me-THF, 160°C, 50 bar of Syngas, 20-44 h, stirring velocity is 380 rpm.
The average yields for 2 experiments are presented. Yields were determined by GC-FID calibration.
*Average yields for 3 experiments.

Table S16. Temperature variation. [Co] = $Co(OPiv)_2$.

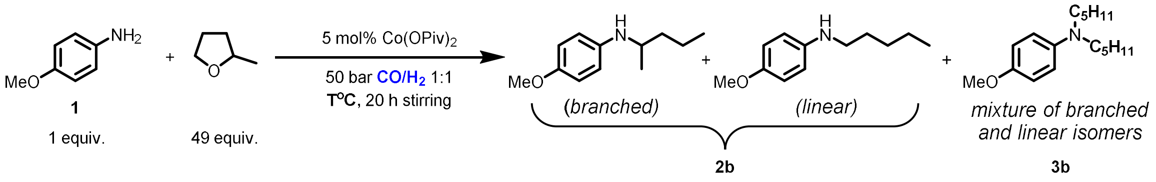

T, °C	Yield of **2b**, %	*branched/linear*	Yield of **3b**, %
140	12	99/1	0
160	53*	74/26	traces
180	73**	73/27	5

18 µmol of [Co], 0.36 mmol of 4-methoxyaniline, 17.6 mmol of 2-Me-THF, 140-180°C, 50 bar of Syngas, 20 h, stirring velocity is 380 rpm.
The average yields for 2 experiments are presented. Yields were determined by GC-FID calibration.
*Average yields for 3 experiments.
**Average yields for 4 experiments.

Table S17. Influence of 2-Me-THF loading. [Co] = $Co(OPiv)_2$.

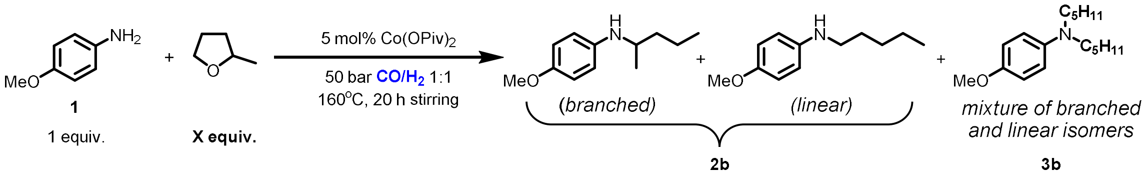

X equiv.*	Yield of **2b**, %	*branched/linear*	Yield of **3b**, %
20	43	74/26	traces
49	53**	74/26	traces
60	60	73/27	traces
80	52	71/29	traces

18 µmol of [Co], 0.36 mmol of 4-methoxyaniline, 17.6 mmol of 2-Me-THF, 140-180°C, 50 bar of Syngas, 20 h, stirring velocity is 380 rpm.
The average yields for 2 experiments are presented. Yields were determined by GC-FID calibration.
*While varying the equivalents of 2-Me-THF, the ratio of gas excess to **1** remained constant.
**Average yields for 3 experiments.

2.2 Control experiments

Intermediates in reaction conditions

N-(but-3-en-1-yl)-4-methoxyaniline was synthesized according to published procedure.[4]

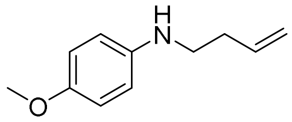

To a solution of 4-methoxyaniline (4 g, 5 equiv., 33.1 mmol) and 4-bromo-1-butene (672 µL, 1 equiv., 6.62 mmol) in EtOH (13 mL) was added NaI (99.3 mg, 0.1 equiv., 0.662 mmol). The mixture was stirred to reflux for 4 h, then the solvent was removed in vacuo. CH_2Cl_2 (20 mL) followed by KOH (1 M, 20 mL) were added. The two-phase mixture was separated, and the organic phase was washed with water (2 × 20 mL) and brine (2 × 20 mL), dried over Na_2SO_4, filtered, and evaporated in vacuo. Target product was isolated as brown oil (0.94 g, 80% yield) after purification by column chromatography (hexane/EtOAc 10/1).

^1H NMR (400 MHz, $CDCl_3$) δ 6.79 (d, J = 8.6 Hz, 2H), 6.60 (d, J = 8.6 Hz, 2H), 5.91 – 5.75 (m, 1H), 5.13 (t, J = 13.6 Hz, 2H), 3.75 (s, 3H), 3.41 (s, 1H), 3.14 (t, J = 6.5 Hz, 2H), 2.38 (d, J = 6.5 Hz, 2H).

NMR data are in agreement with literature data.[4]

4-((4-methoxyphenyl)amino)butan-1-ol was synthesized according to published procedure.[5,6]

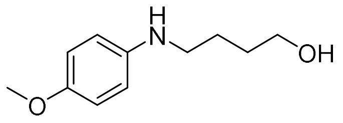

Succinic anhydride (1.02 g, 0.01 mol) was dissolved in a minimum volume of tetrahydrofuran. The volume was brought to 40 mL with ether. To this solution was added dropwise 4-methoxyaniline (1.254 g, 0.01 mol) in 100 mL of ether. The mixture was stirred overnight. Solvent was removed on a rotary evaporator and a pale-yellow powder was obtained. Recrystallization to colorless needles of 4-((4-methoxyphenyl)amino)-4-oxobutanoic acid (0.69 g, 55% yield) proceeded over several days using a 50:50 mixture of THF and hexanes left open to the air.

BH_3*SMe_2 (1.95 mL, 15 M, 18.9 mmol) was added dropwise to a solution of crude 4-((4-methoxyphenyl)amino)-4-oxobutanoic acid (690 mg, 3.1 mmol) in THF (12 mL). Stirring was continued for 2 h, the solution was then refluxed for 12 h and cooled to room temperature. A solution of NaOH (3.74 g) in water (22 mL) was added and the mixture was stirred for 1 h and extracted with EtOAc. The combined organic extracts were washed with brine, dried with Na_2SO_4 and evaporated. Flash chromatography of the residue using EtOAc, gave target product (260 mg, 43%) as a pale-yellow oil.

^{1}H NMR (300 MHz, $CDCl_3$) 6.79 (d, J = 8.6 Hz, 2H), 6.60 (d, J = 8.6 Hz, 2H), 3.75 (s, 3H), 3.68 (t, J = 5.5 Hz, 2H), 3.15 (s, 2H), 2.83 – 2.66 (m, 2H), 1.76 – 1.62 (m, 4H).

NMR data are in agreement with literature data.[6]

5-(butyl(4-methoxyphenyl)amino)pentan-1-ol

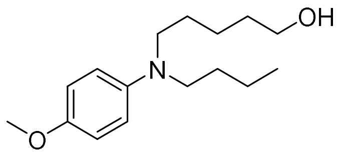

Target product was synthesized according to **General procedure 2**.

MPLC chromatography: $CHCl_3$/MeOH 30/1 (R_f = 0.25). Colorless oil.

^{1}H NMR (400 MHz, $CDCl_3$) δ 6.82 (d, J = 8.9 Hz, 2H), 6.65 (d, J = 8.6 Hz, 2H), 3.75 (s, 3H), 3.64 (t, J = 6.4 Hz, 2H), 3.27 – 3.06 (m, 4H), 1.68 – 1.45 (m, 7H), 1.44 – 1.21 (m, 4H), 0.93 (t, J = 7.3 Hz, 3H).

^{13}C{^{1}H} NMR (101 MHz, $CDCl_3$) δ 115.0, 114.9, 114.6, 63.0, 56.0, 52.0, 32.8, 29.6, 27.3, 23.5, 20.6, 14.2.

^{1}H NMR (400 MHz, DMSO-d_6) δ 6.76 (d, J = 8.1 Hz, 2H), 6.59 (d, J = 8.1 Hz, 2H), 4.44 – 4.31 (m, 1H), 3.64 (s, 3H), 3.44 – 3.28 (m, 2H), 3.26 – 3.05 (m, 4H), 1.54 – 1.35 (m, 6H), 1.35 – 1.20 (m, 4H), 0.89 (t, J = 6.7 Hz, 3H).

^{13}C{^{1}H} NMR (101 MHz, DMSO-d_6) δ 150.4, 142.7, 114.7, 113.9, 60.7, 55.3, 55.0, 51.0, 50.7, 32.5, 29.0, 26.7, 23.1, 19.8, 14.0.

General procedure. $Co(OAc)_2$ (18 μmol, 5 mol%), **intermediate** (0.36 mmol, 100 mol%), magnetic stirrer were charged into a glass vial in 10 ml stainless steel autoclave. 1.4 ml THF (17.6 mmol, 49 equiv.) was added and autoclave was sealed, flushed three times with 5 bar of Syngas (CO/H_2 = 1/1), and then charged with 50 bar of Syngas. The autoclave was placed into a preheated to 160℃ oil bath, stirring velocity corresponded 380 rpm. After 20 h of heating and stirring the autoclave was cooled to room temperature and depressurized. The reaction mixture was transferred in 5 mL graduated vial with CH_2Cl_2. The reaction mixture was centrifuged for 30 min, resulting solution was used for further analysis. Results presented according to GC-FID and GC-MS analyses.

Table S18. N,N-dibutyl-4-methoxyaniline in reaction conditions.

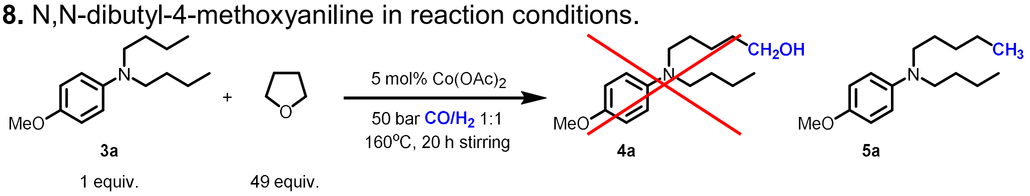

	S(**3a**)/S(**5a**)
Before the reaction	16
After the reaction	12

18 μmol of $Co(OAc)_2$, 0.36 mmol of N,N-dibutyl-4-methoxyaniline, 17.6 mmol of THF, 160°C, 50 bar of Syngas, 20 h, stirring velocity is 380 rpm.
Products areas ratios from GC-FID analysis are presented.

N-butyl-4-methoxy-N-pentylaniline (6% by area ratio) presented in the starting compound. No alcohol formation was recorded after the reaction, at the same time a slight increase in the fraction of impurity N-pentylaniline was observed with low conversion of the initial compound. From the ^{1}H NMR spectrum it is impossible to say unambiguously whether a mixture of isomeric pentyls is formed (according to the chemistry, obviously, it should be), because the doublet and intense triplet signals of the excess of the initial bis-alkylated aniline are superimposed. One peak (N-butyl-4-methoxy-N-pentylaniline) is observed on the chromatogram (GC-FID cipher bai-cat63, April). In fact, it seems like these results are within the margin of error. What can be said - the formation of alcohol is not recorded, so it is not formed from the dehydrogenation-hydroformylation reaction of N,N-dibutyl-4-methoxyaniline (which, in general, is obvious from the regioselectivity of the formed alcohol).

Table S19. 5-(butyl(4-methoxyphenyl)amino)pentan-1-ol in reaction conditions.

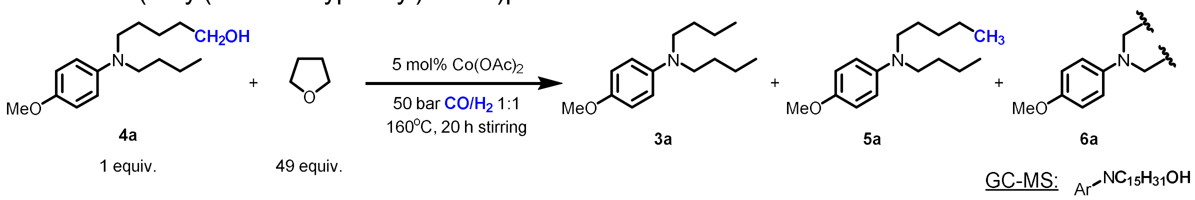

	S(**4a**)/S(**3a**)	S(**4a**)/S(**5a**)	S(**4a**)/S(**6a**)
Before the reaction	302/1	118/1	-
After the reaction	9/1	33/1	15/1

18 μmol of $Co(OAc)_2$, 0.36 mmol of 5-(butyl(4-methoxyphenyl)amino)pentan-1-ol, 17.6 mmol of THF, 160°C, 50 bar of Syngas, 20 h, stirring velocity is 380 rpm.
Products areas ratios from GC-FID analysis are presented.

Table S20. 4-((4-methoxyphenyl)amino)butan-1-ol in reaction conditions.

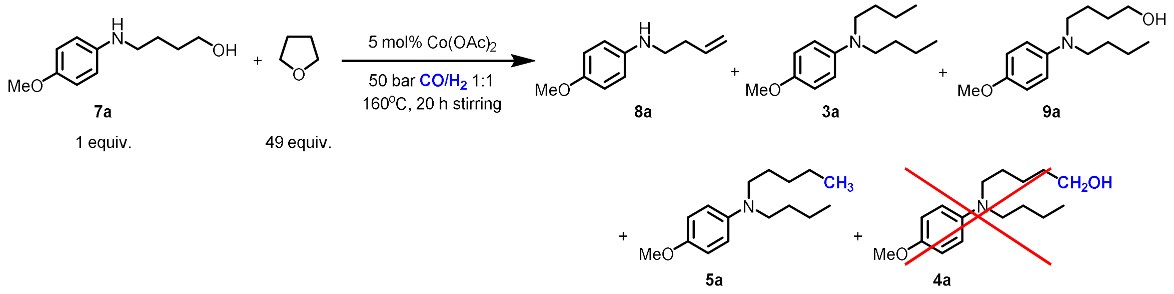

	S(**8a**)	S(**3a**)	S(**9a**)	S(**5a**)
Before the reaction	-	-	-	-
After the reaction	528	367	496	96

18 µmol of $Co(OAc)_2$, 0.36 mmol of 4-((4-methoxyphenyl)amino)butan-1-ol, 17.6 mmol of THF, 160°C, 50 bar of Syngas, 20 h, stirring velocity is 380 rpm.
Products areas ratios from GC-FID analysis are presented.

Full conversion of the initial alcohol.

Table S21. N-(but-3-en-1-yl)-4-methoxyaniline in reaction conditions.

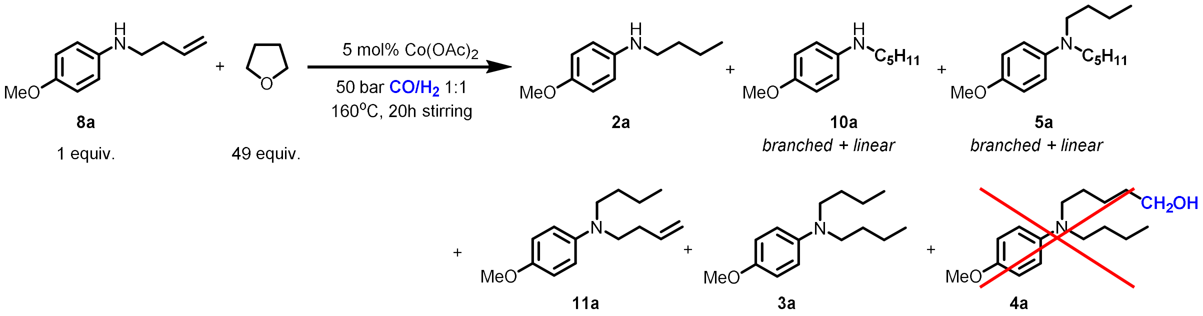

	S(**2a**)	S(**10a**)	S(**5a**)	S(**11a**)	S(**3a**)
Before the reaction	-	-	-	-	-
After the reaction	86	66	28	25	36

18 µmol of $Co(OAc)_2$, 0.36 mmol of N-(but-3-en-1-yl)-4-methoxyaniline, 17.6 mmol of THF, 160°C, 50 bar of Syngas, 20 h, stirring velocity is 380 rpm.
Products areas ratios from GC-FID analysis are presented.

Full conversion of the initial olefin.

Table S22. Alkylation of 4-methoxyaniline with n-butanol instead of THF.

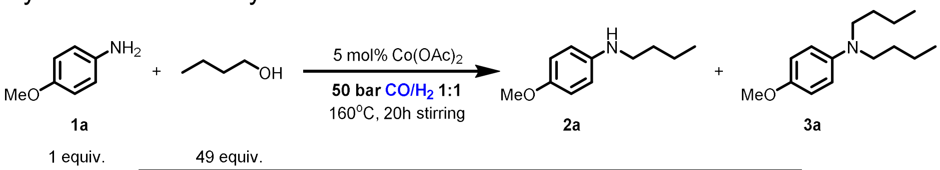

Yield of **1a**, %	Yield of **2a**, %	Yield of **3a**, %
> 85%	traces	0

18 µmol of $Co(OAc)_2$, 0.36 mmol of 4-methoxyaniline, 17.6 mmol of n-BuOH, 160°C, 50 bar of Syngas (CO/H_2 1/1), 20 h, stirring velocity is 380 rpm.
The average yields for 2 experiments are presented. Yields were determined by GC-FID calibration.

Table S23. Alkylation of 4-methoxyaniline with but-3-en-1-ol instead of THF.

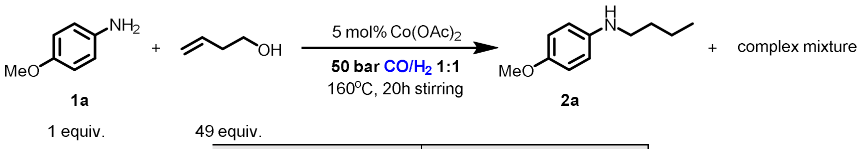

Yield of **1a**, %	Yield of **2a**, %
13%	traces

18 µmol of $Co(OAc)_2$, 0.36 mmol of 4-methoxyaniline, 17.6 mmol of but-3-en-ol, 160°C, 50 bar of Syngas (CO/H_2 1/1), 20 h.

Additional byproducts detection

Table S24. Influence of ratio H_2 presence in reaction system (free volume variation).

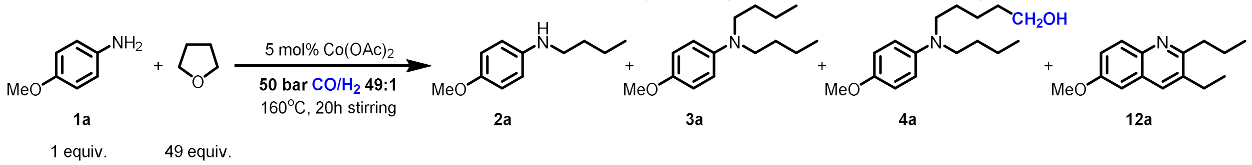

$n(H_2)/n(\mathbf{1a})$	Yield of **2a**, %	Yield of **3a**, %	Yield of **4a**, %	Yield of **12a**, %
0.3	32	0.6	0	10
0.6	38	2	0	6
1	34	1	0	4
2	32	traces	0	traces
16*	0	50	30	0

18 µmol of $Co(OAc)_2$, 0.36 mmol of 4-methoxyaniline, 17.6 mmol of THF, 160°C, 50 bar of Syngas (CO/H_2 49/1), 20 h, stirring velocity is 380 rpm.
The average yields for 2 experiments are presented. Yields were determined by GC-FID calibration.
*Average yields for 17 experiments, CO/H_2 1/1.

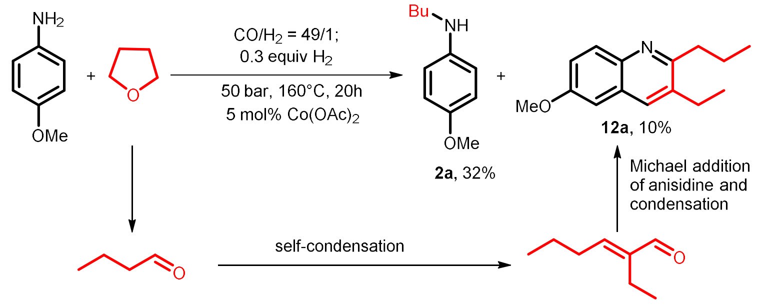

Scheme S1. Possible way for the formation of quinoline byproduct.

Butanal was detected during following reaction procedure.

Pure commercial butanal was analyzed by GC-MS, the peak at 1.88 min with $[M]^+$ = 72 and the fragment $[M-15]^+$ was identified as target peak.

4.4 mg of 4-methoxyaniline (36 µmol, 100 mol%), magnetic stirrer, 1 or 1.43 mL THF (12.1 mmol, 340 equiv. or 17.6 mmol, 490 equiv.) were charged into a glass vial in 10 ml stainless steel autoclave. Aliquot of $Co(OPiv)_2$ (18 µmol, 50 mol%) (stock solution in THF, c=10 mg/mL) or $Co(OAc)_2$ (18 µmol, 50 mol%), was added and autoclave was sealed, flushed three times with 5 bar of Syngas, and then charged with 50 bar of Syngas (CO/H_2 = 1/1). The autoclave was placed into a preheated to 160℃ oil bath, stirring velocity corresponded 380 rpm. After 4 h of heating and stirring the autoclave was cooled to room temperature and depressurized. The reaction mixture was transferred in 5 mL graduated vial with CH_2Cl_2. An aliquot (80 µL) was diluted to 1 mL with DMSO, resulting solution was used for further GC-MS analysis.

The amine loading was lowered to prevent butanal reaction with amine excess to increase butanal detection probability.

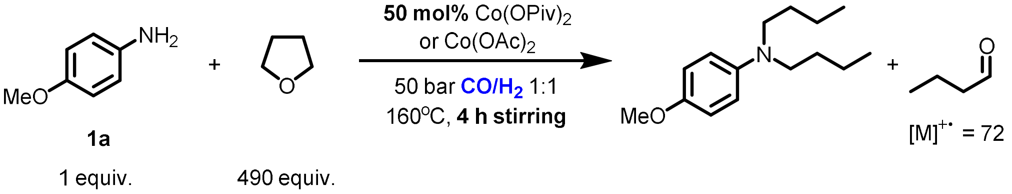

18 µmol of $Co(OPiv)_2$ or $Co(OAc)_2$, 36 µmol of 4-methoxyaniline, 17.6 mmol of THF, 160°C, 50 bar of Syngas (CO/H_2 1/1), 20 h, stirring velocity is 380 rpm.

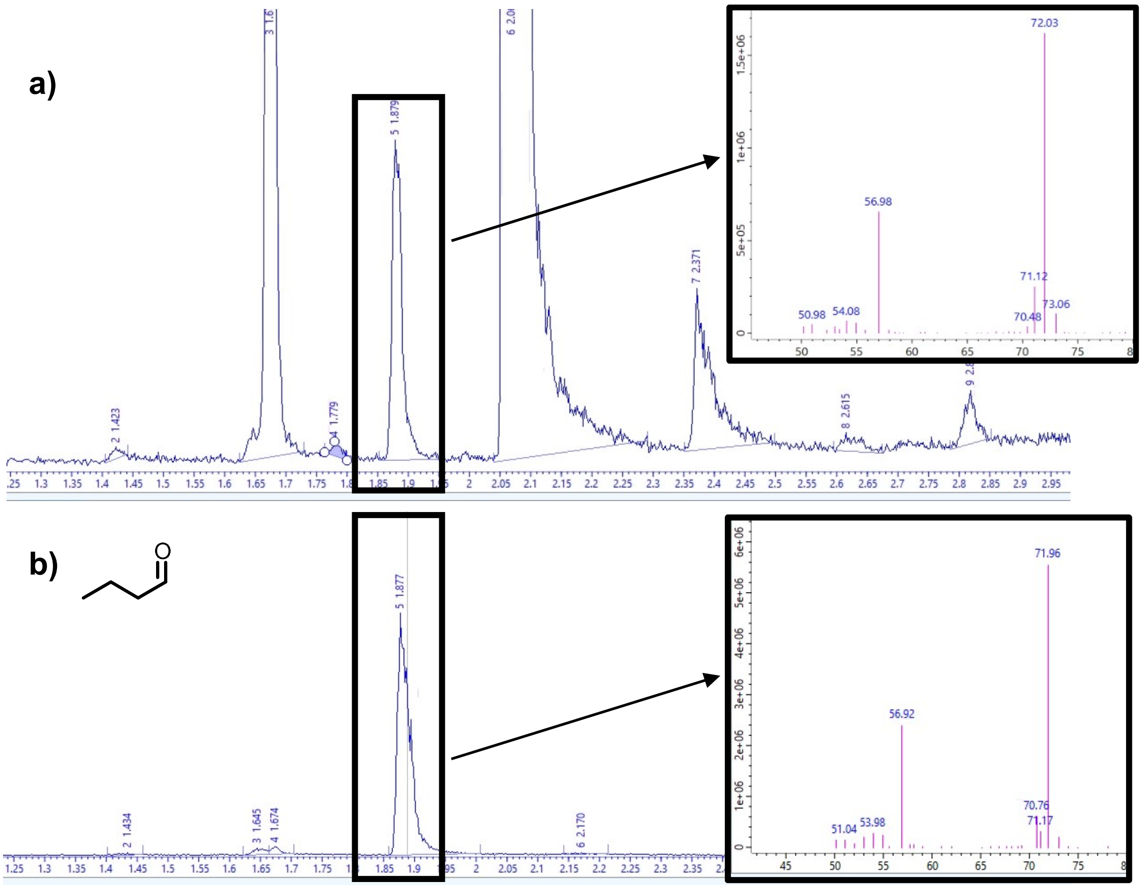

Figure S1. a) chromatogram of reaction mixture containing the peak at 1.88 min of target butanal with corresponding mass spectrum.
b) chromatogram of pure butanal (peak at 1.88 min) with corresponding mass spectrum.

The following components of the reaction mixture were detected in the chromatogram region of interest.

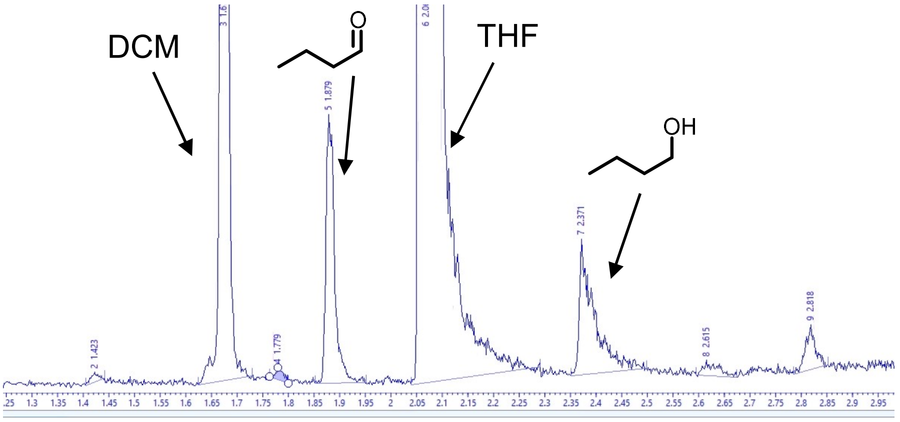

Figure S2. Detection of aliphatic by-products in reaction mixture.

An experiment was carried out without introducing amine into the reaction system, resulting in a complex mixture of aliphatic products.

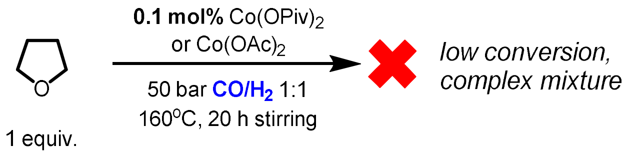

18 µmol of $Co(OPiv)_2$ or $Co(OAc)_2$, 17.6 mmol of THF, 160°C,
50 bar of Syngas (CO/H_2 1/1), 20 h, stirring velocity is 380 rpm.

Scheme S2. Experiment without an aromatic amine in the reaction system.

Alkylation of 4-methoxyaniline with d_8-THF

Reaction between 4-methoxyaniline and d_8-THF was carried out according to General procedure 1. The results of ^1H NMR and GC-MS analysis are consistent with the structure **2a'** illustrating that the process is likely to proceed via THF-ring opening and formation of the corresponding aldehyde. Then, reductive amination occurs with 4-methoxyaniline furnishing the product.

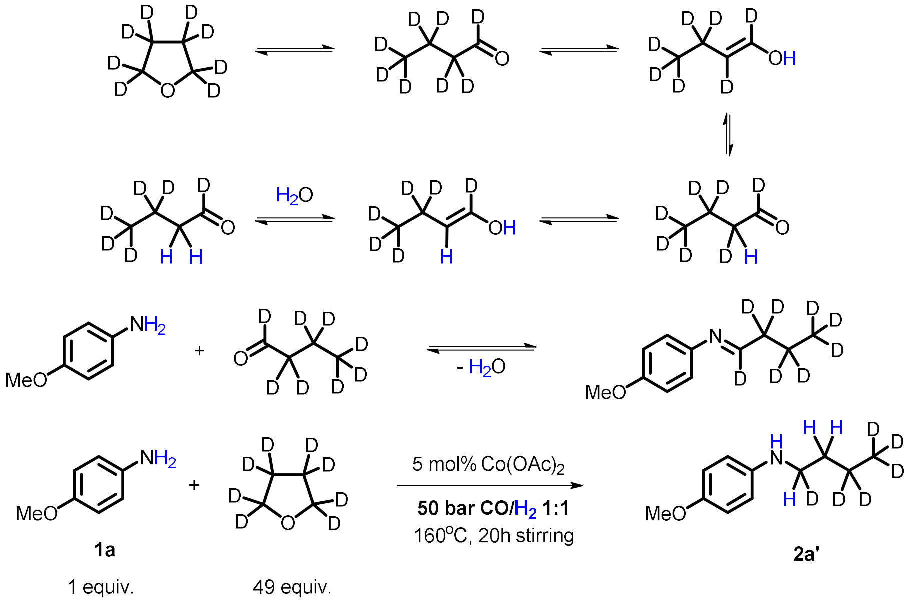

Scheme S3. Control experiment: alkylation of 4-methoxyaniline with d_8-THF.

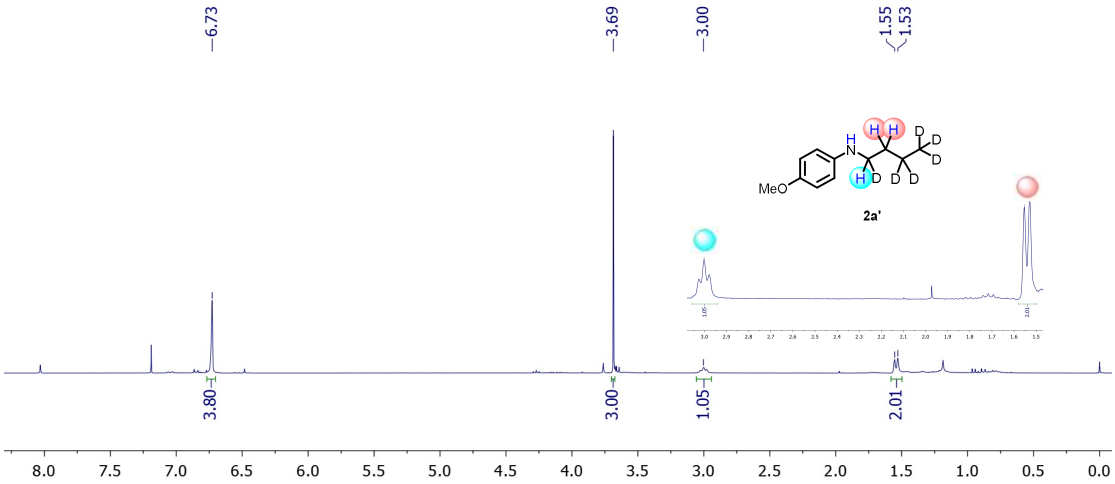

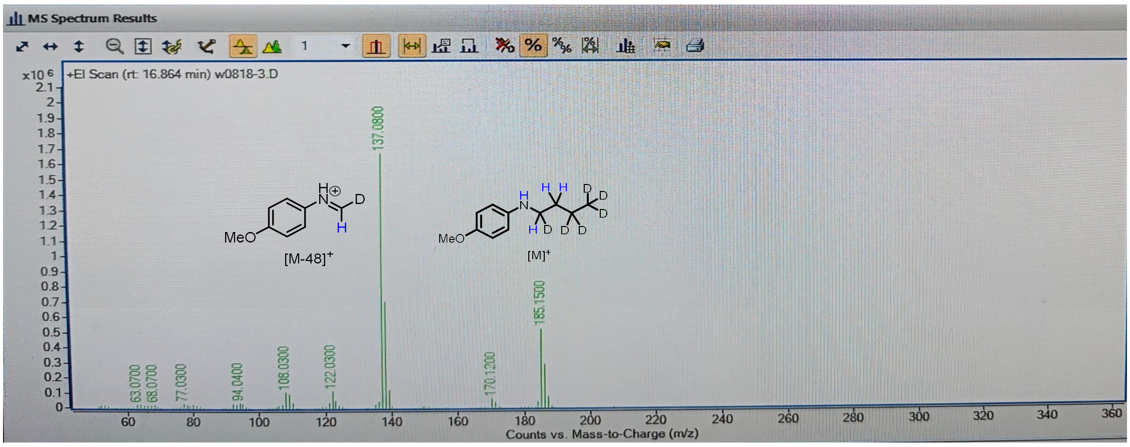

Figure S3. ^{1}H NMR spectrum and GC-MS chromatogram of 4-methoxyaniline alkylation with d_8-THF reaction mixture.

Influence of mercury addition on alkylation process[7]

44.3 mg of 4-methoxyaniline (0.36 mmol, 100 mol%), magnetic stirrer, 1 mL THF (12.1 mmol, 34 equiv) were charged into a glass vial in 10 mL stainless steel autoclave. Aliquot of $Co(OPiv)_2$ (18 μmol, 5 mol%) (stock solution in THF, c=10 mg/mL), was added and autoclave was sealed, charged with 50 bar of Syngas (CO/H_2 = 1/1). The autoclave was placed into a preheated to 160℃ oil bath, stirring velocity corresponded 380 rpm. After 5 h of heating and stirring the autoclave was cooled to room temperature, depressurized. Autoclave was opened, 9 μL Hg (0.61 mmol, 1.7 equiv.) was added, then autoclave was sealed, charged with 50 bar of Syngas (CO/H_2 = 1/1). The autoclave was placed into a preheated to 160℃ oil bath, stirring velocity corresponded 380 rpm. After 4 h of heating and stirring the autoclave was cooled to room temperature, depressurized. The reaction mixture was transferred in 5 mL graduated vial with CH_2Cl_2.

Reference experiment included depressurizing and autoclave opening to be sure that all reaction conditions were symmetrical except the mercury addition.

Table S25. Influence of mercury addition.

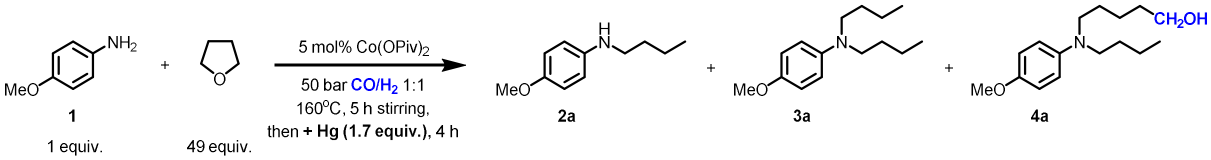

Reaction time, h	Hg addition	Yield of **2a**, %	Yield of **3a**, %	Yield of **4a**, %
5 + 4	none*	22	26	13
	+	58	traces	8
5**	none	35	5	4

18 μmol of $Co(OPiv)_2$, 0.36 mmol of 4-methoxyaniline, 17.6 mmol of THF, 160°C, 50 bar of Syngas (CO/H_2 1/1), 5 h, then 0.61 mmol of Hg, 160°C, 50 bar of Syngas (CO/H_2 1/1), 4 h, stirring velocity is 380 rpm.
Yields were determined by GC-FID calibration.
*Average yields for 2 experiments.
**Average yields for 2 experiments without additional heating time.

2.3 Unsuccessful substrates

Bridging bonds destruction, complex mixture of alkylated anilines

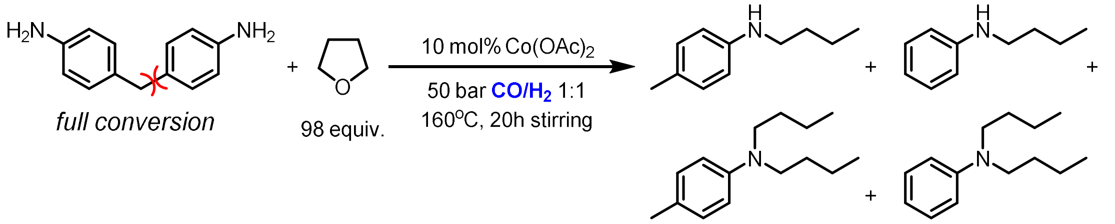

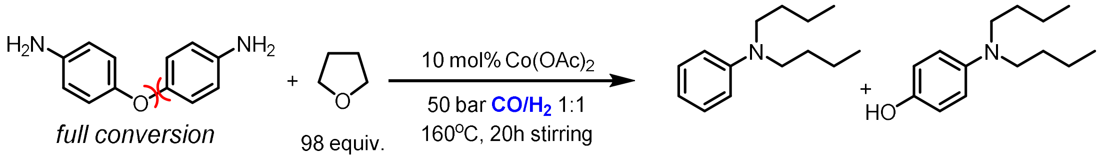

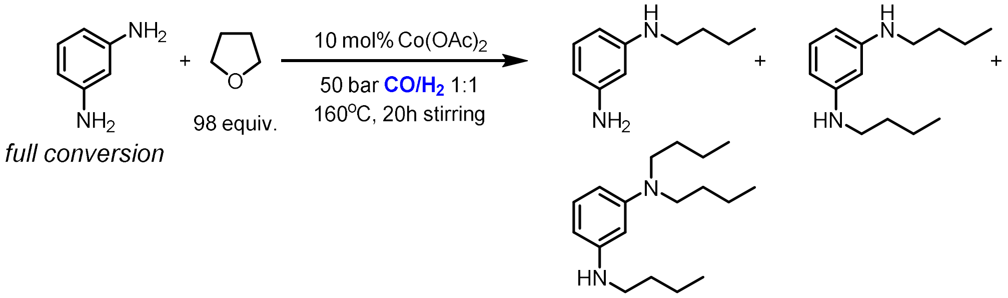

a mixure of products impossible to isolate individually due to easy oxidation

Functional group reduction

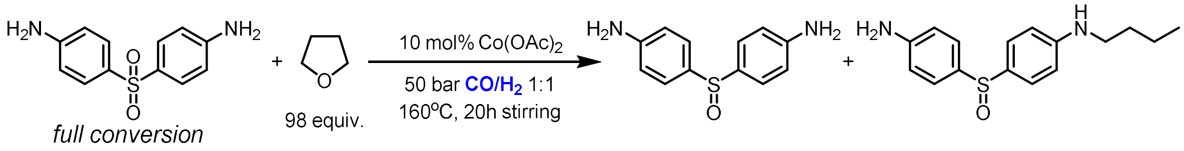

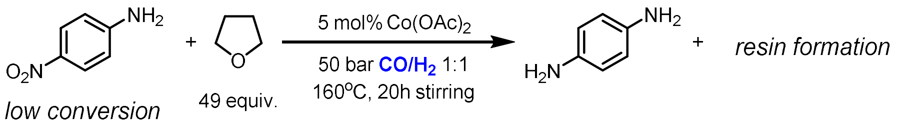

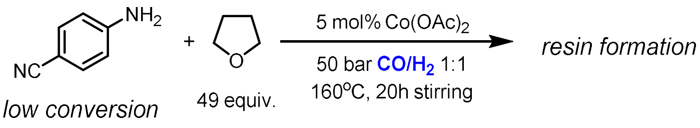

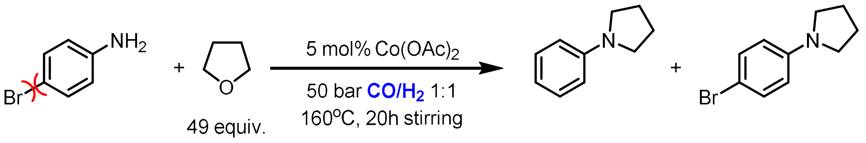

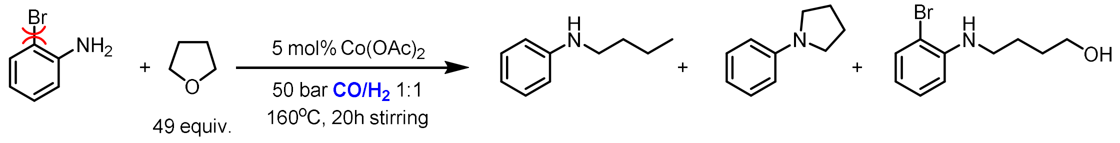

Ethers as alkylating agents: limitations

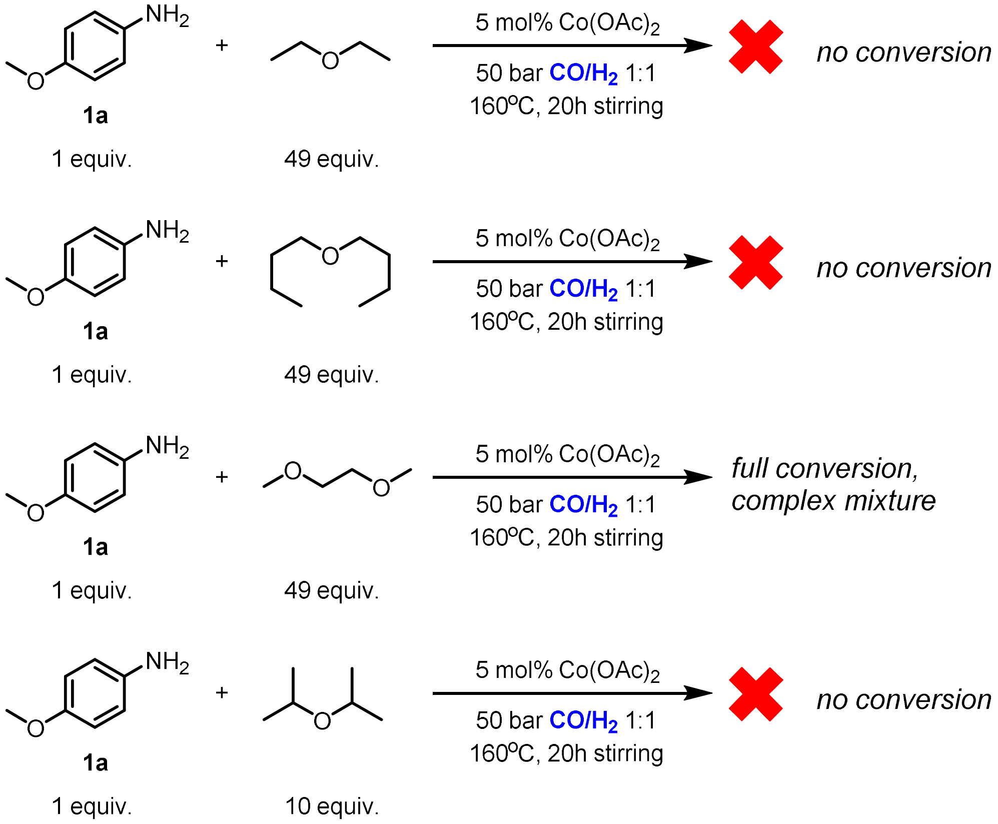

18 μmol of $Co(OAc)_2$, 0.36 mmol of 4-methoxyaniline, 3.6 or 17.6 mmol of ether, 160°C,
50 bar of Syngas (CO/H_2 1/1), 20 h, stirring velocity is 380 rpm.
The average yields for 2 experiments are presented.
Conversion and main products were determined by GC-FID calibration and GC-MS data analysis.

Cyclic ethers as alkylating agents: limitations

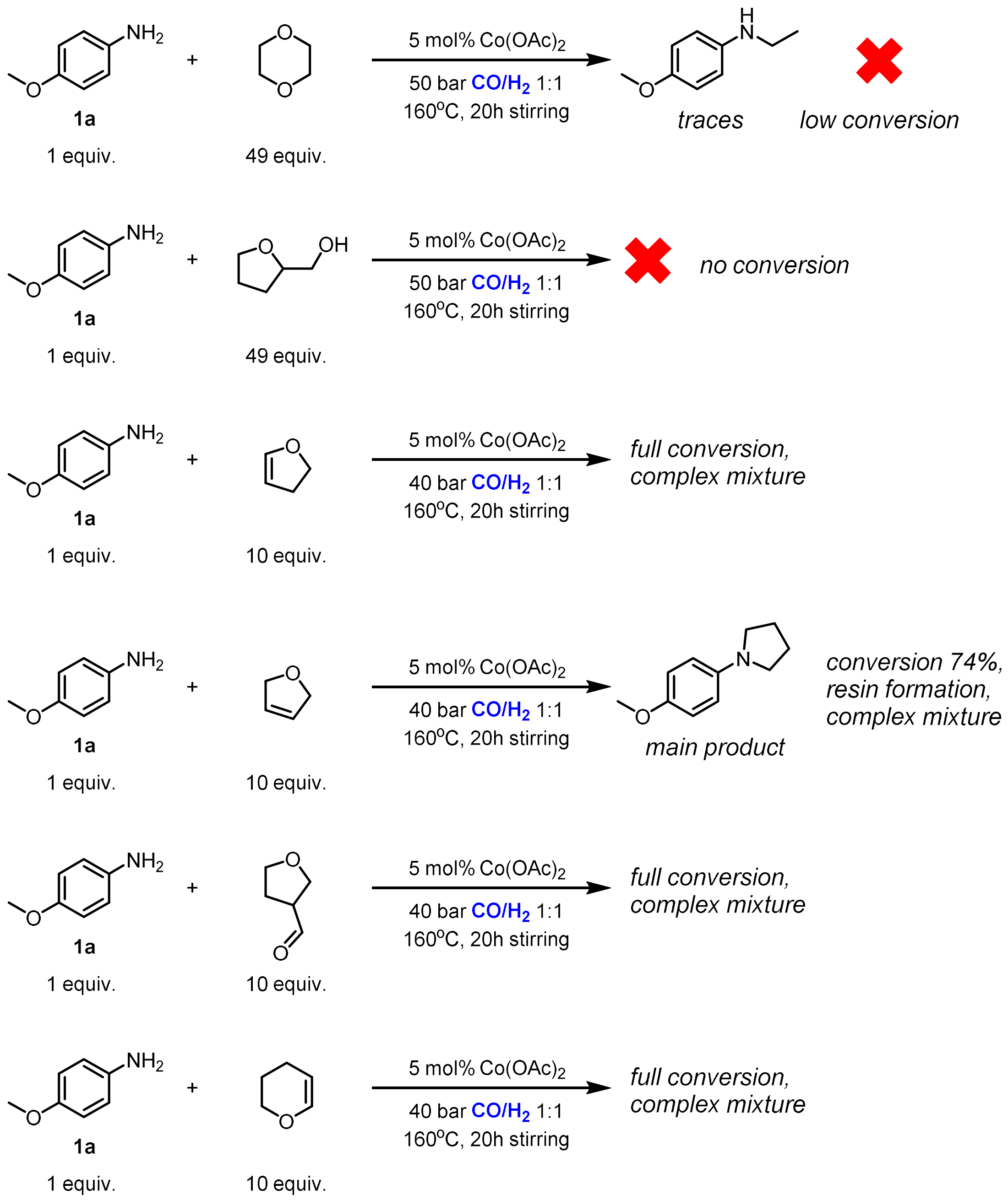

18 μmol of $Co(OAc)_2$, 0.36 mmol of 4-methoxyaniline, 3.6 or 17.6 mmol of ether, 160°C, 50 or 40 bar of Syngas (CO/H_2 1/1), 20 h, stirring velocity is 380 rpm.
The average yields for 2 experiments are presented.
Conversion and main products were determined by GC-FID calibration and GC-MS data analysis.

2.4 Spectroscopic and analytical data

N-butyl-4-methoxyaniline (2a)

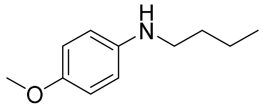

Product was synthesized according to **General procedure 1**.

Conditions: 10 mol% $Co(OAc)_2$, 150°C.

Yellow oil, 52 mg, 81% yield, (eluent: petroleum ether/ethyl acetate = 20:1).

^{1}H NMR (300 MHz, $CDCl_3$) δ 6.82 – 6.76 (m, 2H), 6.63 – 6.58 (m, 2H), 3.75 (s, 3H), 3.45 (br s, 1H), 3.07 (t, *J* = 7.1 Hz, 2H), 1.65 – 1.55 (m, 2H), 1.49 – 1.37 (m, 2H), 0.96 (t, *J* = 7.3 Hz, 3H).

^{13}C{^{1}H} NMR (75 MHz, $CDCl_3$) δ 152.3, 142.7, 115.0, 114.4, 56.0, 45.0, 31.8, 20.4, 14.1.

N-butyl-3-methoxyaniline (2b)

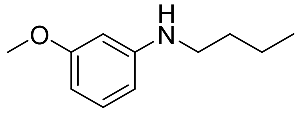

Product was synthesized according to **General procedure 1**.

Conditions: 10 mol% $Co(OAc)_2$, 150°C.

Colorless oil, 36 mg, 56% yield, (eluent: petroleum ether/ethyl acetate = 20:1).

^{1}H NMR (400 MHz, $CDCl_3$) δ 7.09 (t, *J* = 8.1 Hz, 1H), 6.29 – 6.23 (m, 2H), 6.19 – 6.18 (m, 1H), 3.79 (s, 3H), 3.72 (br s, 1H), 3.11 (t, *J* = 7.2 Hz, 2H), 1.65 – 1.57 (m, 2H), 1.49 – 1.39 (m, 2H), 0.97 (t, *J* = 7.4 Hz, 3H).

^{13}C{^{1}H} NMR (101 MHz, $CDCl_3$) δ 161.0, 150.0, 130.0, 106.1, 102.3, 98.7, 55.2, 43.8, 31.7, 20.4, 14.0.

NMR data are in agreement with literature data.[8]

N-butyl-2-methoxyaniline (2c)

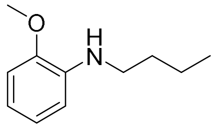

Product was synthesized according to **General procedure 1**.

Conditions: 10 mol% $Co(OAc)_2$, 150°C.

Yellow oil, 50 mg, 78% yield, (eluent: petroleum ether/ethyl acetate = 20:1).

^{1}H NMR (300 MHz, $CDCl_3$) δ 6.91 – 6.85 (m, 1H), 6.79 – 6.75 (m, 1H), 6.68 – 6.60 (m, 2H), 3.85(s, 3H), 3.13 (t, J = 7.1 Hz, 2H), 1.70 – 1.59 (m, 2H), 1.52 – 1.39 (m, 2H), 0.97 (t, J = 7.3 Hz, 3H).

^{13}C{^{1}H} NMR (75 MHz, $CDCl_3$) δ 146.9, 138.7, 121.4, 116.2, 109.8, 109.5, 55.5, 43.5, 31.8, 20.5, 14.1.

NMR data are in agreement with literature data.[8]

***N*-butyl-4-methylaniline (2d)**

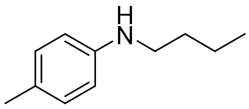

Product was synthesized according to **General procedure 1**.

Conditions: 10 mol% $Co(OAc)_2$, 140°C.

Yellow oil, 48 mg, 82% yield, (eluent: petroleum ether/ethyl acetate = 40:1).

^{1}H NMR (300 MHz, $CDCl_3$) δ 7.02 – 6.97 (m, 2H), 6.60 – 6.55 (m, 2H), 3.10 (t, *J* = 7.1 Hz, 2H), 2.25 (s, 3H), 1.66 – 1.56 (m, 2H), 1.49 – 1.37 (m, 2H), 0.96 (t, *J* = 7.3 Hz, 3H).

$^{13}C\{^1H\}$ NMR (75 MHz, $CDCl_3$) δ 146.0, 129.9, 126.9, 113.4, 44.5, 31.7, 20.5, 20.4, 14.1.

1-(4-(butylamino)phenyl)ethan-1-one (2e)

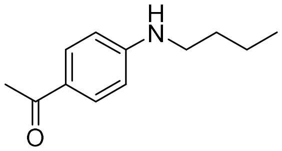

Product was synthesized according to **General procedure 1**.

Conditions: 10 mol% $Co(OAc)_2$, 140°C.

White solid, 32 mg, 47% yield, (eluent: petroleum ether/ethyl acetate = 10:1).

^{1}H NMR (300 MHz, $CDCl_3$) δ 7.84 – 7.79 (m, 2H), 6.56 – 6.52 (m, 2H), 4.20 (br s, 1H), 3.18 (t, *J* = 7.1 Hz, 2H), 2.49 (s, 3H), 1.67 – 1.57 (m, 2H), 1.49 – 1.37 (m, 2H), 0.96 (t, *J* = 7.3 Hz, 3H).

$^{13}C\{^1H\}$ NMR (75 MHz, $CDCl_3$) δ 196.4, 152.5, 131.0, 126.6, 111.4, 43.1, 31.5, 26.1, 20.3, 14.0.

NMR data are in agreement with literature data.[9]

Methyl 4-(butylamino)benzoate (2f)

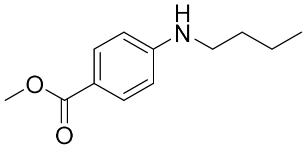

Product was synthesized according to **General procedure 1**.

Conditions: 10 mol% $Co(OAc)_2$, 150°C.

White solid, 48 mg, 65% yield, (eluent: petroleum ether/ethyl acetate = 10:1).

^{1}H NMR (300 MHz, $CDCl_3$) δ 7.88 – 7.83 (m, 2H), 6.59 – 6.51 (m, 2H), 3.84 (s, 3H), 3.16 (t, J = 7.1 Hz, 2H), 1.66 – 1.55 (m, 2H), 1.49 – 1.35 (m, 2H), 0.96 (t, J = 7.3 Hz, 3H).

$^{13}C\{^1H\}$ NMR (75 MHz, $CDCl_3$) δ 167.5, 152.1, 131.7, 118.3, 111.6, 51.6, 43.3, 31.5, 20.3, 14.0.

NMR data are in agreement with literature data.[10]

***N*-butyl-4-((trifluoromethyl)thio)aniline (2g)**

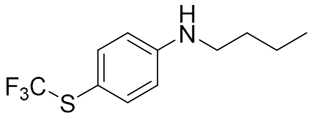

Product was synthesized according to **General procedure 1**.

Conditions: 10 mol% $Co(OAc)_2$, 150°C.

Yellow oil, 60 mg, 67% yield, (eluent: petroleum ether/ethyl acetate = 40:1).

^{1}H NMR (300 MHz, $CDCl_3$) δ 7.44 – 7.40 (m, 2H), 6.60 – 6.55 (m, 2H), 4.16 (br s, 1H), 3.13 (t, *J* = 7.1 Hz, 2H), 1.67 – 1.57 (m, 2H), 1.50 – 1.37 (m, 2H), 0.97 (t, *J* = 7.3 Hz, 3H).

^{13}C{^{1}H} NMR (75 MHz, $CDCl_3$) δ 149.9, 138.3, 129.9 (q, C-F, $^1J_{C\text{-}F}$ = 5.6 Hz), 113.7, 110.3, 43.9, 31.3, 20.3, 14.0.

^{19}F NMR (282 MHz, $CDCl_3$) δ: -44.6.

HRMS (ESI) of $C_{11}H_{14}F_3NS$, *m/z*: calcd for $[M+H]^+$ 250.0872, found: 250.0878.

***N*-butyl-2-(trifluoromethyl)aniline (2i)**

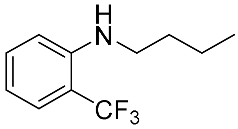

Product was synthesized according to **General procedure 1**.

Conditions: 10 mol% $Co(OAc)_2$, 140°C.

Yellow oil, 44 mg, 56% yield, (eluent: petroleum ether/ethyl acetate = 40:1).

^{1}H NMR (300 MHz, $CDCl_3$) δ 7.45 – 7.41 (m, 1H), 7.40 – 7.33 (m, 1H), 6.76 – 6.68 (m, 2H), 3.18 (t, J = 7.1 Hz, 2H), 1.71 – 1.61 (m, 2H), 1.51 – 1.38 (m, 2H), 0.98 (t, J = 7.3 Hz, 3H).

^{13}C{^{1}H} NMR (75 MHz, $CDCl_3$) δ 145.8, 133.2, 126.7 (q, C-F, $^3J_{C\text{-}F}$ = 5.6 Hz), 125.7 (q, C-F, $^1J_{C\text{-}F}$ = 271.0 Hz), 115.9, 112.1, 43.6, 31.3, 20.3, 14.0.

^{19}F NMR (282 MHz, $CDCl_3$) δ: -62.4.

***N*-butylaniline (2j)**

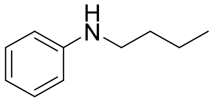

Product was synthesized according to **General procedure 1**.

Conditions: 10 mol% $Co(OAc)_2$, 140°C.

Colorless oil, 43 mg, 80% yield, (eluent: petroleum ether/ethyl acetate = 40:1).

^{1}H NMR (300 MHz, $CDCl_3$) δ 7.22 – 7.15 (m, 2H), 6.75 – 6.63 (m, 3H), 3.12 (t, *J* = 7.1 Hz, 2H), 1.67 – 1.57 (m, 2H), 1.49 – 1.37 (m, 2H), 0.96 (t, *J* = 7.3 Hz, 3H).

^{13}C{^{1}H} NMR (75 MHz, $CDCl_3$) δ 148.1, 129.4, 117.8, 113.3, 44.2, 31.6, 20.4, 14.0.

***N*-butylnaphthalen-1-amine (2k)**

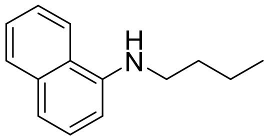

Product was synthesized according to **General procedure 1**.

Conditions: 5 mol% $Co(OAc)_2$, 150°C.

Colorless oil, 50 mg, 70% yield, (eluent: petroleum ether/ethyl acetate = 30:1).

^{1}H NMR (400 MHz, $CDCl_3$) δ 7.88 – 7.84 (m, 2H), 7.53 – 7.40 (m, 3H), 7.31 – 7.28 (m, 1H), 6.69 (d, *J* = 7.5 Hz, 1H)., 4.46 (br s, 1H), 3.33 (t, *J* = 7.1 Hz, 2H)., 1.86 – 1.78 (m, 2H), 1.63 – 1.54 (m, 2H), 1.10 – 1.05 (m, 3H).

^{13}**C NMR** (101 MHz, $CDCl_3$) δ 143.6, 134.4, 128.7, 126.7, 125.7, 124.7, 123.4, 119.9, 117.2, 104.4, 44.0, 31.6, 20.6, 14.1.

NMR data are in agreement with literature data.[11]

***N,N*-dibutyl-4-methoxyaniline (3a)**

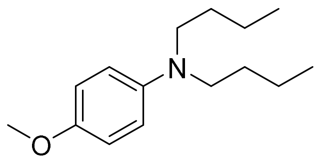

Product was synthesized according to **General procedure 2**.

Reaction conditions: 160°C, 30 bar of Syngas (CO/H_2 = 1/1), 20 h.

MPLC chromatography: hexane/EtOAc 30/1. Yellow oil.

Yield in reaction mixture: 72%. Isolated yield: 42 mg (50%).

^{1}H NMR (400 MHz, $CDCl_3$) δ 6.83 (d, *J* = 8.7 Hz, 2H), 6.67 (d, *J* = 8.7 Hz, 2H), 3.77 (s, 3H), 3.27 – 3.13 (m, 4H), 1.62 – 1.45 (m, 4H), 1.43 – 1.25 (m, 4H), 0.95 (t, *J* = 7.2 Hz, 6H).

^{13}C{^{1}H} NMR (101 MHz, $CDCl_3$) δ 151.1, 143.4, 114.9, 114.4, 56.0, 51.8, 29.6, 20.6, 14.2.

NMR data are in agreement with literature data.[12]

***N,N*-dibutyl-4-methylaniline (3d)**

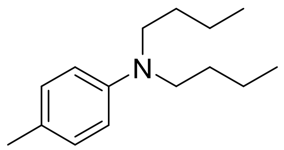

Product was synthesized according to **General procedure 2**.

Column chromatography: hexane/EtOAc 40/1, R_f = 0.36. Beige oil.

Yield in reaction mixture: 43%. Isolated yield: 32 mg (41%).

^{1}H NMR (400 MHz, $CDCl_3$) δ: 7.03 (d, *J* = 8.6 Hz, 2H), 6.59 (d, *J* = 8.6 Hz, 2H), 3.30 – 3.19 (m, 4H), 2.25 (s, 3H), 1.56 (tt appears as quint., *J* = 7.5 Hz, 4H), 1.35 (tq appears as h, *J* = 7.3 Hz, 4H), 0.96 (t, *J* = 7.3 Hz, 6H).

^{13}C{^{1}H} NMR (101 MHz, $CDCl_3$) δ 146.4, 129.8, 124.5, 112.4, 51.1, 29.6, 20.5, 20.3, 14.2.

NMR data are in agreement with literature data.[12]

***N,N*-dibutylaniline (3j)**

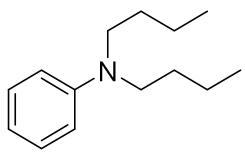

Product was synthesized according to **General procedure 2**.

Reaction conditions: 160°C, 30 bar of syngas (CO/H_2 = 1/1), 20 h.

Preparative TLC: hexane/MTBE/Et_3N 40/1,6/1, R_f = 0.52. Yellow oil.

Yield in reaction mixture: 83%. Isolated yield: 40.1 mg (53%). Purity is 93%. There is the impurity of branched N-butyl-N-octyl-aniline (7%) which has very similar R_f in chromatography.

^{1}H NMR (400 MHz, $CDCl_3$) δ: 7.24 (t, J = 8.0Hz, 2H), 6.69-6.61 (m, 3H), 3.32-3.28 (m, 4H), 1.61 (tt appears as quint., *J* = 8.0 Hz, 4H), 1.39 (tq appears as h, *J* = 7.4 Hz, 4H), 1.00 (t, J = 7.3 Hz, 6H).

^{13}C{^{1}H} NMR (101 MHz, $CDCl_3$) δ: 148.3, 129.3, 115.2, 111.8, 50.9, 29.5, 20.5, 14.2.

NMR data are in agreement with literature data.[12]

***N,N*-dibutyl-3-(trifluoromethyl)aniline (3l)**

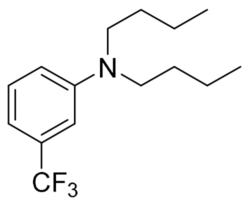

Product was synthesized according to **General procedure 2**.

Preparative TLC: hexane/CH_2Cl_2/Et_3N 40/4/1, R_f = 0.61. Beige oil.

Yield in reaction mixture: 60%. Isolated yield: 48 mg (49%).

^{1}H NMR (300 MHz, $CDCl_3$) δ: 7.29 – 7.23 (m, 1H), 6.85 – 6.74 (m, 3H), 3.31 – 3.26 (m, 4H), 1.62 – 1.52 (m, 4H), 1.42 – 1.30 (m, 4H), 0.96 (t, *J* = 7.3 Hz, 6H).

^{13}C{^{1}H} NMR (101 MHz, $CDCl_3$) δ 148.3, 131.6 (q, *J* = 31.3 Hz), 129.7, 124.8 (q, *J* = 272.4 Hz), 114.6, 111.5 (q, *J* = 3.9 Hz), 107.9 (q, *J* = 3.9 Hz), 50.9, 29.4, 20.4, 14.1.

^{19}F NMR (376 MHz, $CDCl_3$) δ: -62.8.

NMR data are in agreement with literature data.[12]

***N,N*-dibutyl-4-fluoroaniline (3m)**

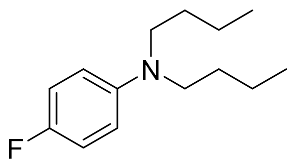

Product was synthesized according to **General procedure 2**.

Preparative TLC: hexane/EtOAc/Et_3N 40/2/1, R_f = 0.7. Yellow oil.

Yield in reaction mixture: 67%. Isolated yield: 32.6 mg (41%).

^{1}H NMR (400 MHz, $CDCl_3$) δ: 6.97 – 6.86 (m, 2H), 6.63 – 6.53 (m, 2H), 3.27 – 3.17 (m, 4H), 1.54 (tt appears as quint., *J* = 7.5 Hz, 4H), 1.34 (tq appears as h, *J* = 7.4 Hz, 4H), 0.95 (t, *J* = 7.3 Hz, 6H).

^{13}C{^{1}H} NMR (101 MHz, $CDCl_3$) δ: 154.9 (d, *J* = 233.7 Hz), 145.2, 115.6 (d, *J* = 21.8 Hz), 113.2 (d, *J* = 7.0 Hz), 51.5, 29.5, 20.5, 14.2.

^{19}F NMR (376 MHz, $CDCl_3$) δ: -130.8

NMR data are in agreement with literature data.[12]

***N,N*-dibutyl-2,6-dimethylaniline (3n)**

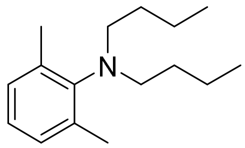

Product was synthesized according to **General procedure 2**.

Preparative TLC: hexane/EtOAc/Et_3N 40/4/1, R_f = 0.76. Beige oil.

Yield in reaction mixture: 53%. Isolated yield: 43 mg (51%).

^{1}H NMR (400 MHz, $CDCl_3$) δ: 7.01 – 6.93 (m, 3H), 3.01 – 2.97 (m, 4H), 2.29 (s, 6H), 1.41 (tt appears as quint., *J* = 7.4 Hz, 4H), 1.31 – 1.24 (m, 4H), 0.88 (t, *J* = 7.3 Hz, 6H).

^{13}C{^{1}H} NMR (101 MHz, $CDCl_3$) δ 148.8, 138.1, 128.8, 124.8, 54.1, 32.0, 20.7, 19.8, 14.3.

HRMS (ESI) of $C_{16}H_{27}N$, *m/z*: calcd for $[M+H]^+$ 234.2216, found: 234.2212.

***N,N*-dibutyl-2,6-diisopropylaniline (3o)**

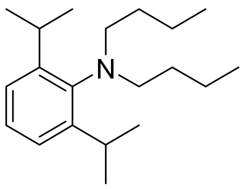

Product was synthesized according to **General procedure 2**.

Flash chromatography: hexane/EtOAc 50/1. Colorless oil.

Yield in reaction mixture: 54%. Isolated yield: 47.2 mg (46%).

^{1}H NMR (400 MHz, $CDCl_3$) δ: 7.24 – 7.06 (m, 3H), 3.53 (h, *J* = 6.9 Hz, 2H), 3.07 – 2.95 (m, 4H), 1.45 (m, 4H), 1.34 – 1.25 (m, 4H), 1.20 (d, *J* = 6.9 Hz, 12H), 0.90 (t, *J* = 7.3 Hz, 6H).

^{13}C{^{1}H} NMR (101 MHz, $CDCl_3$) δ: 149.5, 146.5, 126.1, 124.1, 56.4, 32.7, 27.9, 24.7, 20.7, 14.3.

HRMS (ESI) of $C_{20}H_{35}N$, *m/z*: calcd for $[M+H]^+$ 290.2842, found: 290.2842.

Ethyl 4-(dibutylamino)benzoate (3p)

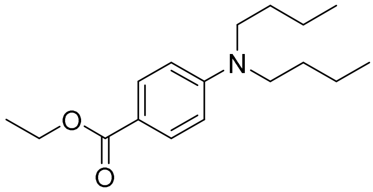

Product was synthesized according to **General procedure 2**.

Preparative TLC: hexane/CH_2Cl_2/Et_3N 30/10/1, R_f = 0.8. Orange oil.

Yield in reaction mixture: 56%. Isolated yield: 33.3 mg (42%).

^{1}H NMR (400 MHz, $CDCl_3$) δ: 7.87 (d, J = 8.7 Hz, 2H), 6.57 (d, J = 8.7 Hz, 2H), 4.31 (q, J = 7.0 Hz, 2H), 3.33 – 3.29 (m, 4H), 1.62 – 1.54 (m, 4H), 1.40 – 1.31 (m, 7H), 0.96 (t, J = 7.3 Hz, 6H).

^{13}C{^{1}H} NMR (101 MHz, $CDCl_3$) δ 167.1, 151.4, 131.5, 116.4, 110.3, 60.1, 50.8, 29.4, 20.4, 14.6, 14.1.

NMR data are in agreement with literature data.[13]

Mixture of 4-methoxy-N-(pentan-2-yl)aniline and 4-methoxy-*N*-pentyl-aniline

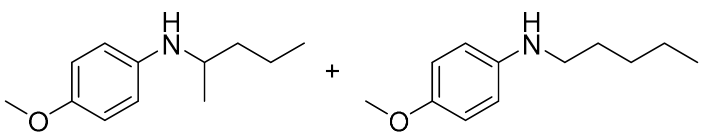

Products were synthesized according to **General procedure 2** (2-Me-THF as alkylating agent).

Preparative TLC: hexane/acetone/Et_3N 30/10/1, R_f = 0.62. Orange oil.

Yield in reaction mixture for the sum of products: 79% (branched/linear = 73/27). Isolated yield: 41.3 mg (59%, branched/linear = 83/17).

^{1}H NMR (400 MHz, $CDCl_3$) δ: 6.80-6.74 (m, 2H), 6.59-6.53 (m, 2H), 3.74 (s, 3H), 3.43-3.33 (m, 1H), 1.63-1.49 (m, 1H), 1.46-1.32 (m, 3H), 1.15 (d, J = 6.3 Hz, 3H), 0.92 (t, J = 7.0 Hz, 3H).

4-methoxy-*N*-(pentan-2-yl)aniline

$^{13}C\{^1H\}$ NMR (101 MHz, $CDCl_3$) δ 151.9, 142.0, 115.0, 114.8, 55.9, 49.4, 39.5, 20.9, 19.4, 14.3.

NMR data are in agreement with literature data.[14]

4-methoxy-*N*-pentylaniline

$^{13}C\{^1H\}$ NMR (101 MHz, $CDCl_3$) δ 152.0, 142.9, 114.96, 114.1, 55.8, 45.1, 29.5, 22.6, 14.2.

NMR data are in agreement with literature data.[15]

3. References

[1] M. Strohalm, D. Kavan, P. Novák, M. Volný, V. Havlíček, *Anal. Chem.* **2010**, *82*, 4648–4651.

[2] G. Aromí, A. S. Batsanov, P. Christian, M. Helliwell, A. Parkin, S. Parsons, A. A. Smith, G. A. Timco, R. E. P. Winpenny, *Chem. – A Eur. J.* **2003**, *9*, 5142–5161.

[3] B. E. Bryant, W. C. Fernelius, D. H. Busch, R. C. Stoufer, W. Stratton, in *Inorg. Synth.* (Ed.: Therald Moeller), **1957**, pp. 188–189.

[4] V. Durel, C. Lalli, T. Roisnel, P. van de Weghe, *J. Org. Chem.* **2016**, *81*, 849–859.

[5] R. Kluger, J. C. Hunt, *J. Am. Chem. Soc.* **1989**, *111*, 5921–5925.

[6] D. L. J. Clive, J. Peng, S. P. Fletcher, V. E. Ziffle, D. Wingert, *J. Org. Chem.* **2008**, *73*, 2330–2344.

[7] D. Gärtner, S. Sandl, A. Jacobi von Wangelin, *Catal. Sci. Technol.* **2020**, *10*, 3502–3514.

[8] K. Okano, H. Tokuyama, T. Fukuyama, *Org. Lett.* **2003**, *5*, 4987–4990.

[9] L. Huang, R. Yu, X. Zhu, Y. Wan, *Tetrahedron* **2013**, *69*, 8974–8977.

[10] Y. Pan, Z. Luo, J. Han, X. Xu, C. Chen, H. Zhao, L. Xu, Q. Fan, J. Xiao, *Adv. Synth. Catal.* **2019**, *361*, 2301–2308.

[11] W. Chen, K. Chen, W. Chen, M. Liu, H. Wu, *ACS Catal.* **2019**, *9*, 8110–8115.

[12] T. J. Barker, E. R. Jarvo, *J. Am. Chem. Soc.* **2009**, *131*, 15598–15599.

[13] S. Urgaonkar, J.-H. Xu, J. G. Verkade, *J. Org. Chem.* **2003**, *68*, 8416–8423.

[14] C. Qin, T. Shen, C. Tang, N. Jiao, *Angew. Chemie Int. Ed.* **2012**, *51*, 6971–6975.

[15] D. Jiang, H. Fu, Y. Jiang, Y. Zhao, *J. Org. Chem.* **2007**, *72*, 672–674.

4. ^{1}H, ^{13}C, ^{19}F NMR and HRMS spectra of obtained compounds

^{1}H NMR of N-butyl-4-methoxyaniline (300 MHz, $CDCl_3$) (2a)

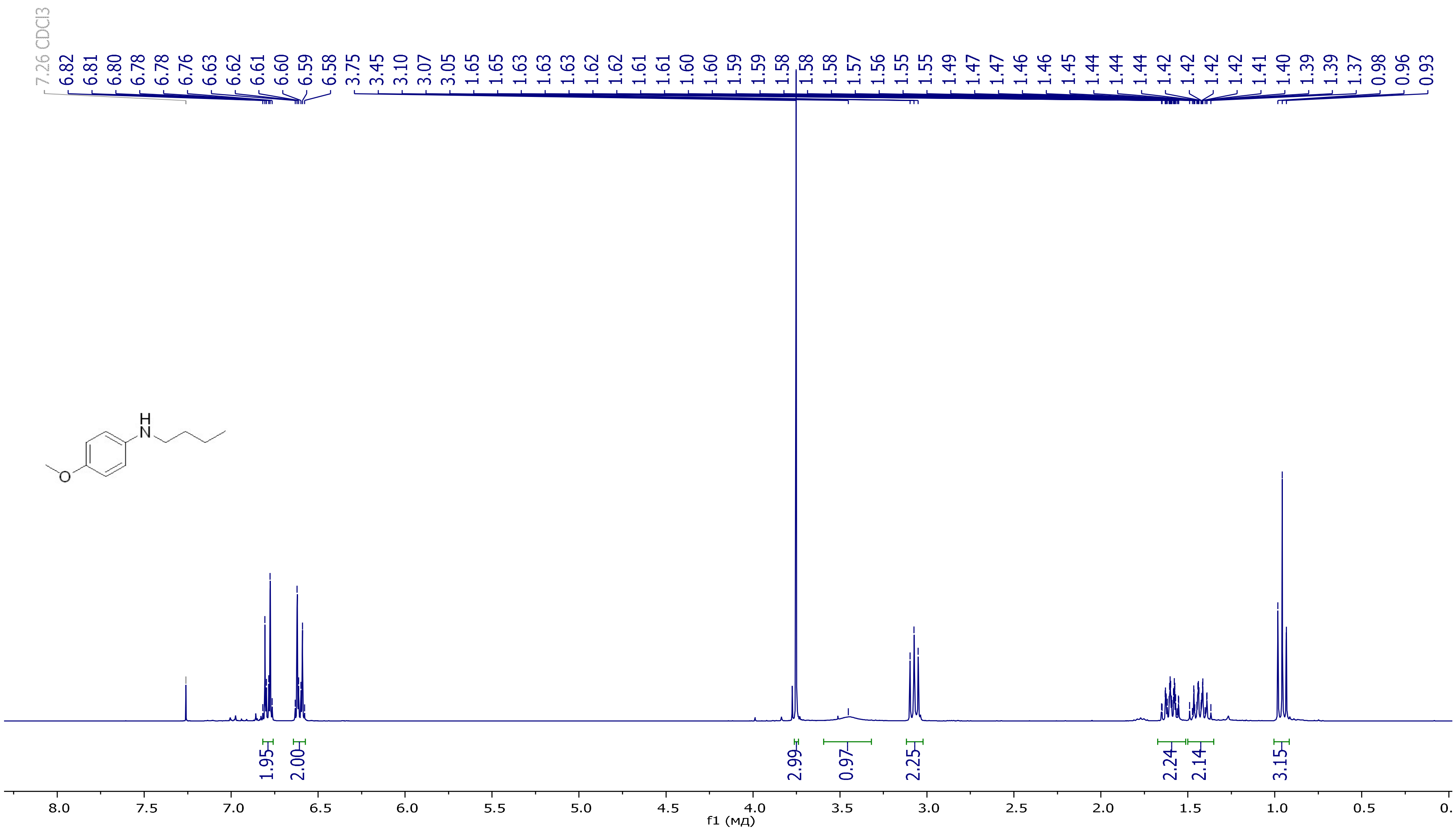

$^{13}C\{^1H\}$ NMR of N-butyl-4-methoxyaniline (75 MHz, $CDCl_3$) (2a)

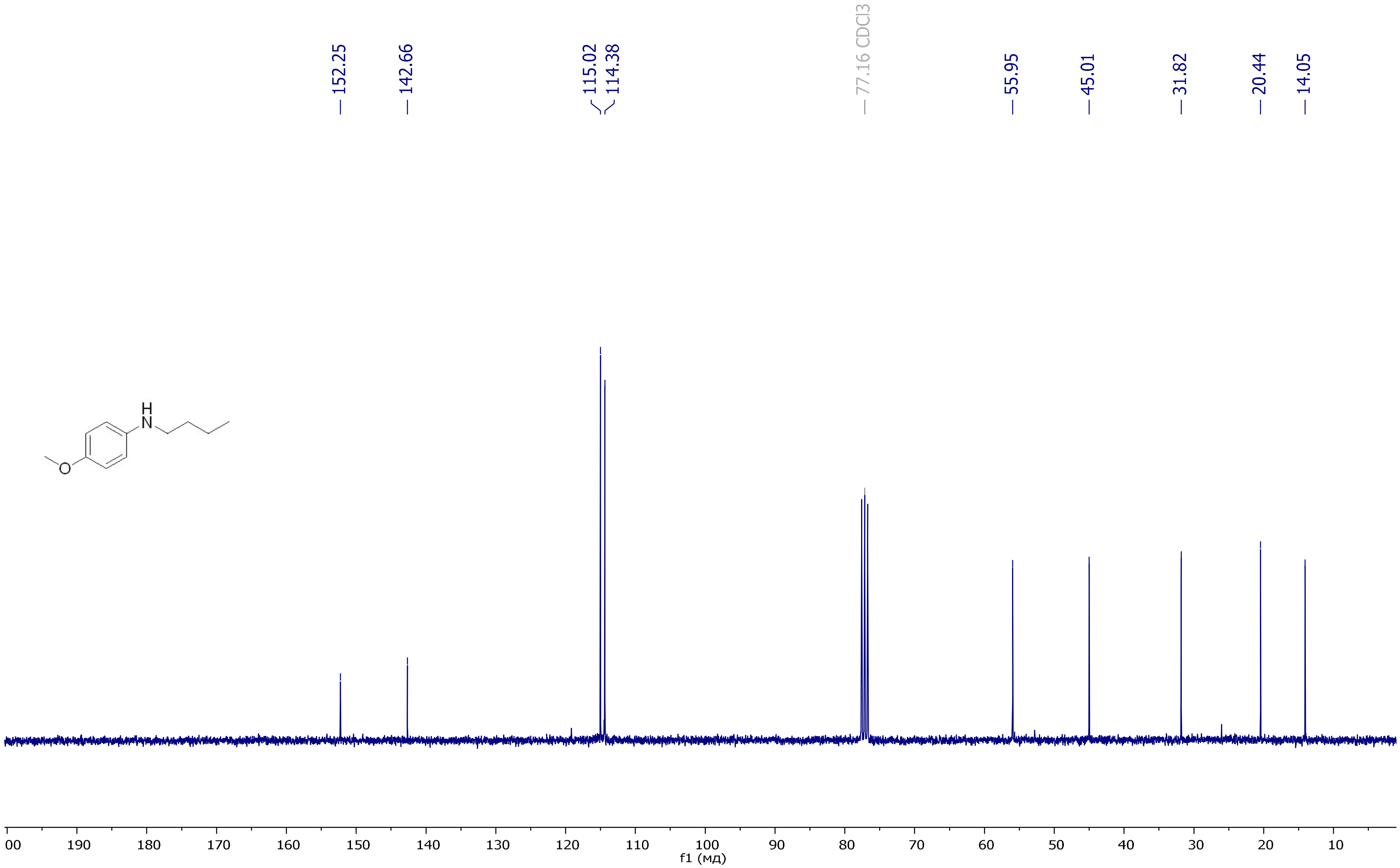

^{1}H NMR of N-butyl-3-methoxyaniline (400 MHz, $CDCl_3$) (2b)

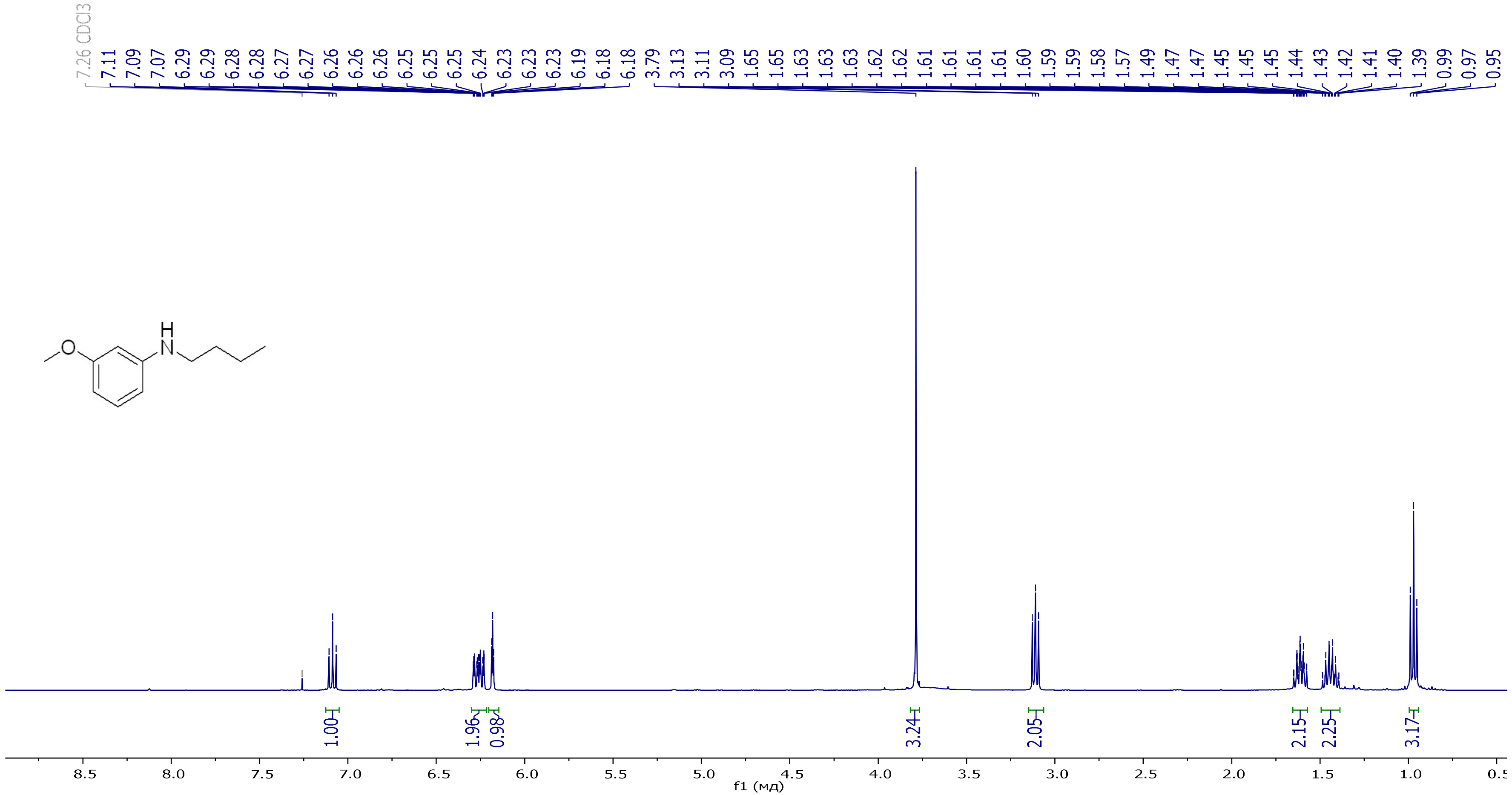

$^{13}C\{^{1}H\}$ NMR of N-butyl-3-methoxyaniline (101 MHz, $CDCl_3$) (2b)

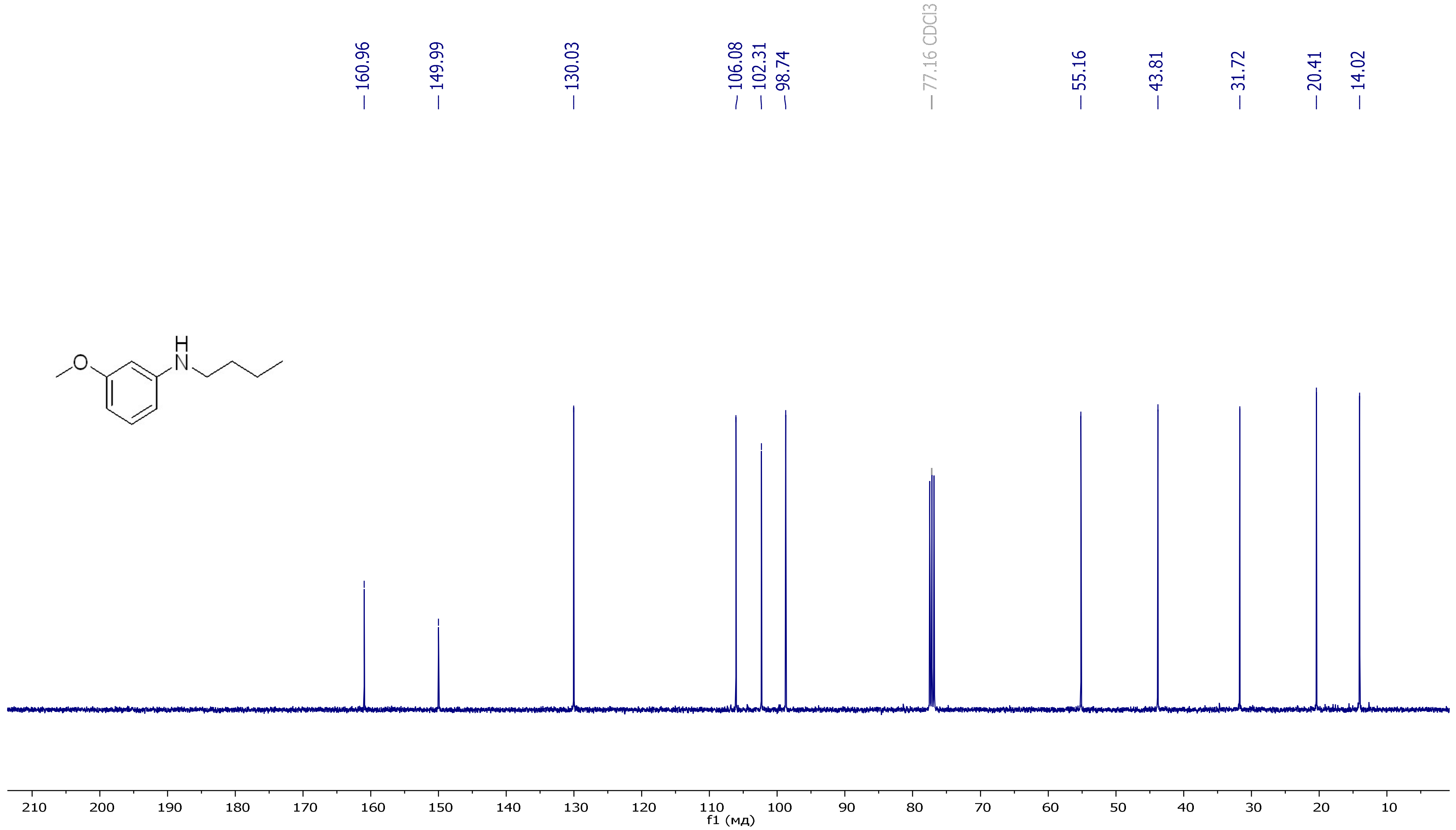

^{1}H NMR of N-butyl-2-methoxyaniline (300 MHz, $CDCl_3$) (2c)

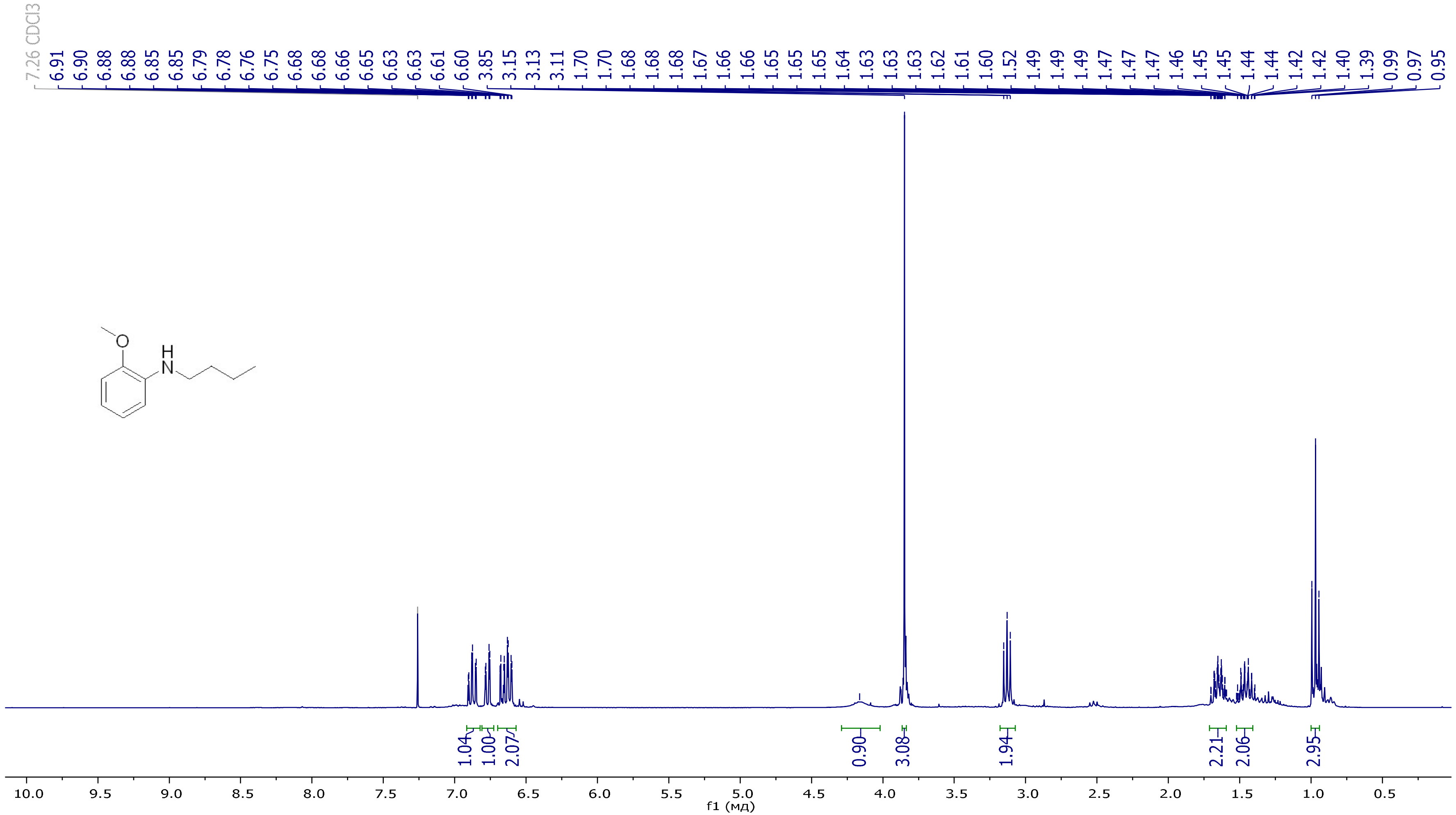

$^{13}C\{^1H\}$ NMR of N-butyl-2-methoxyaniline (75 MHz, $CDCl_3$) (2c)

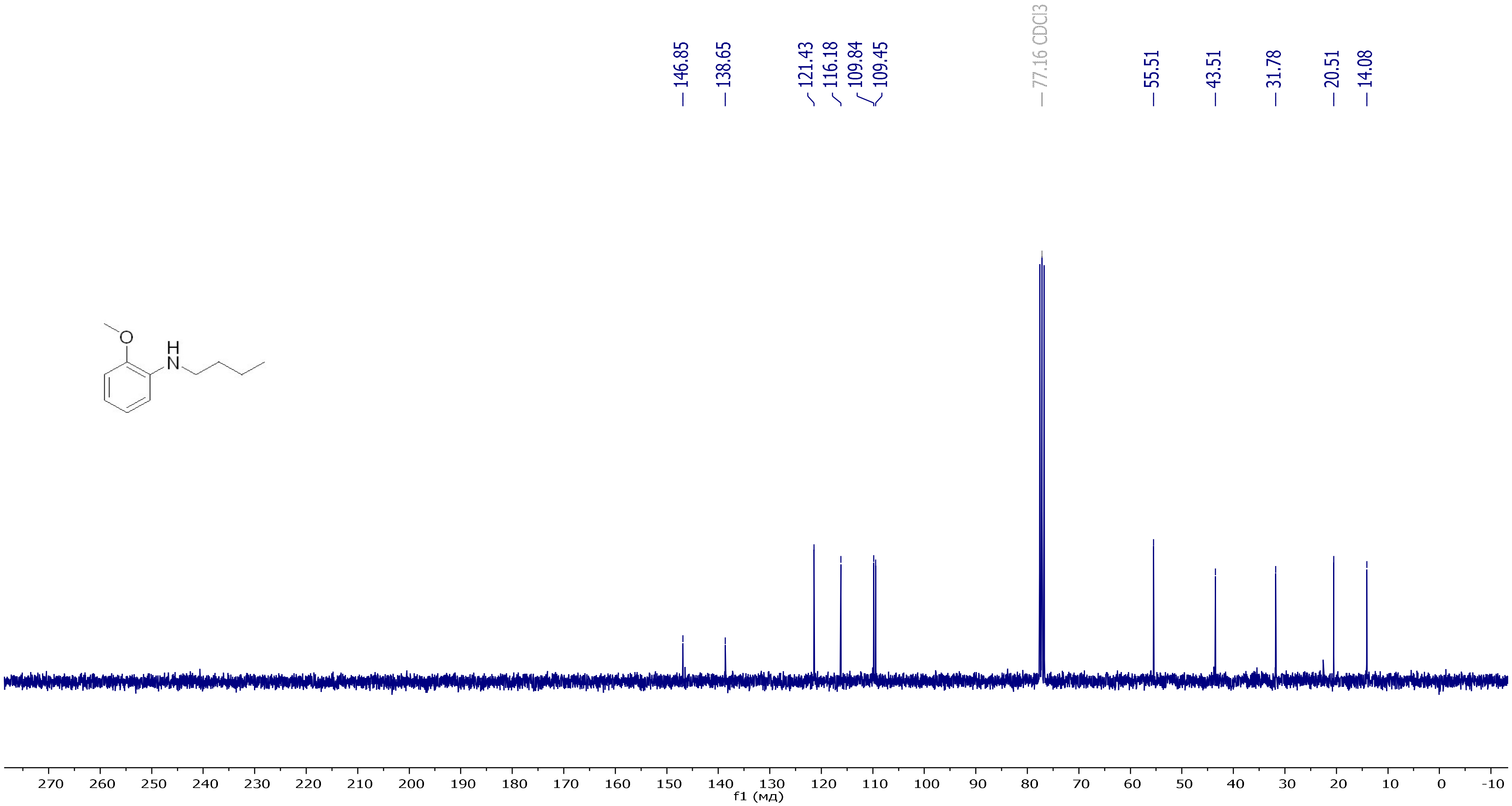

^{1}H NMR of N-butyl-4-methylaniline (300 MHz, $CDCl_3$) (2d)

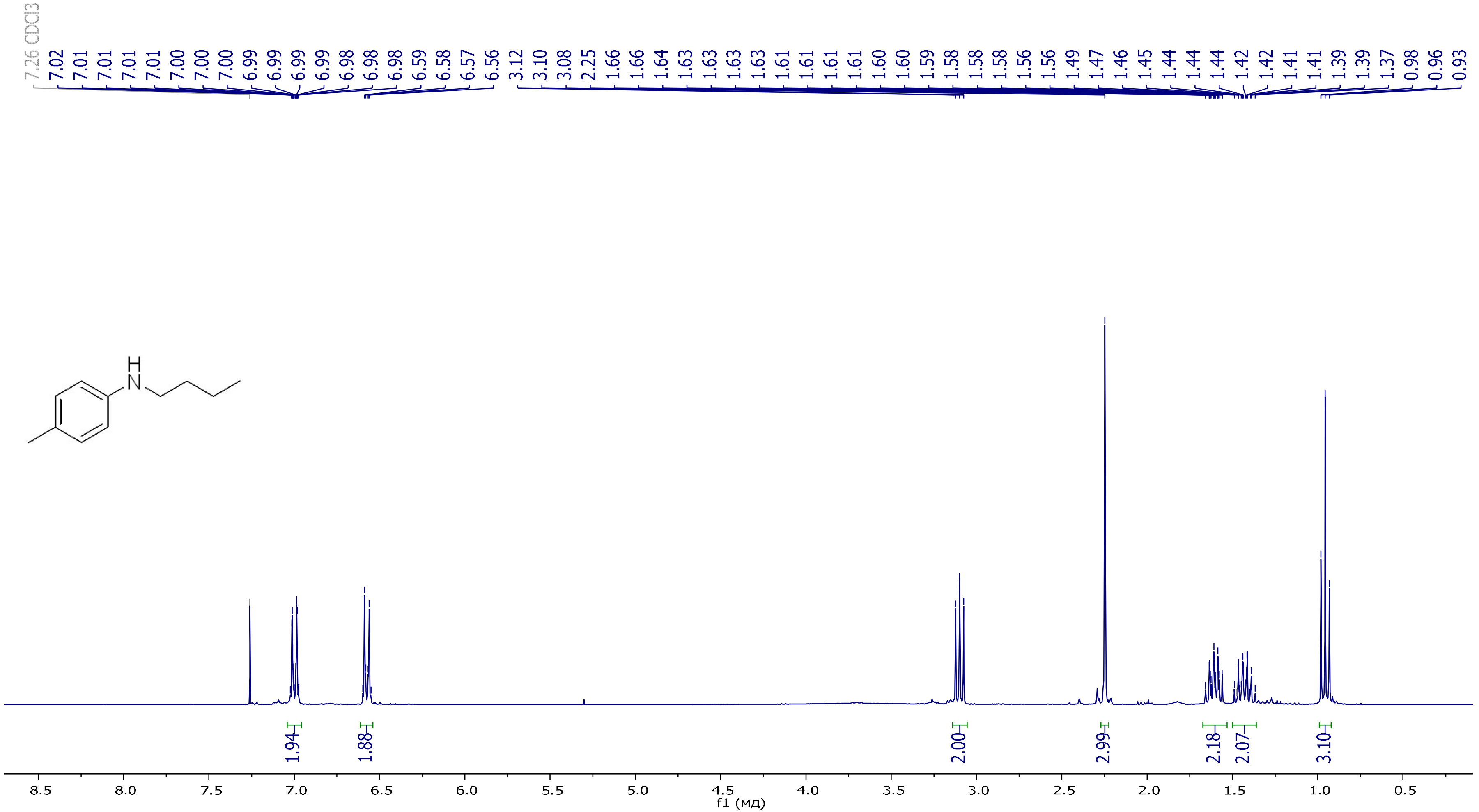

$^{13}C\{^1H\}$ NMR of N-butyl-4-methylaniline (75 MHz, $CDCl_3$) (2d)

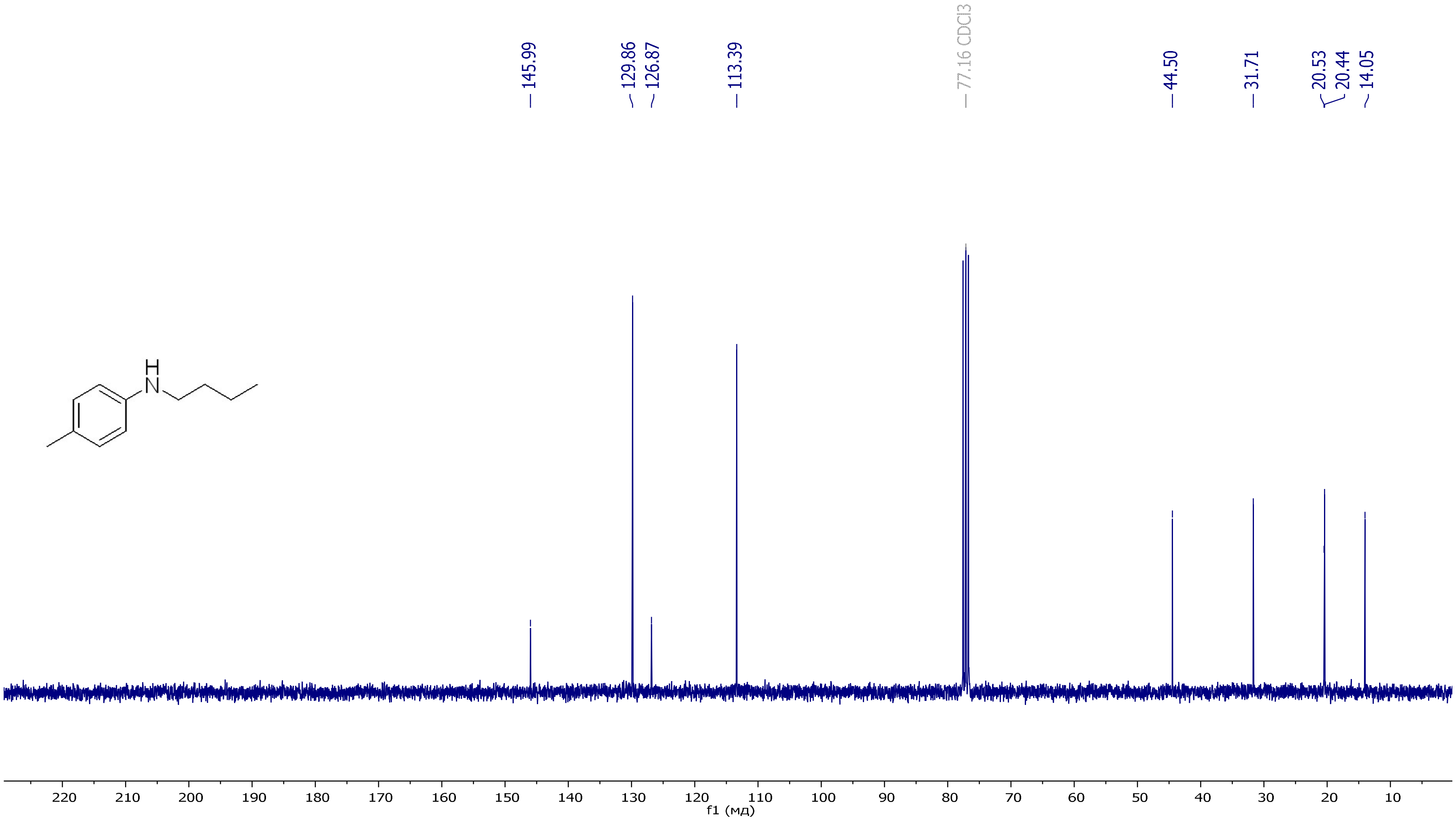

^{1}H NMR of 1-(4-(butylamino)phenyl)ethan-1-one (300 MHz, $CDCl_3$) (2e)

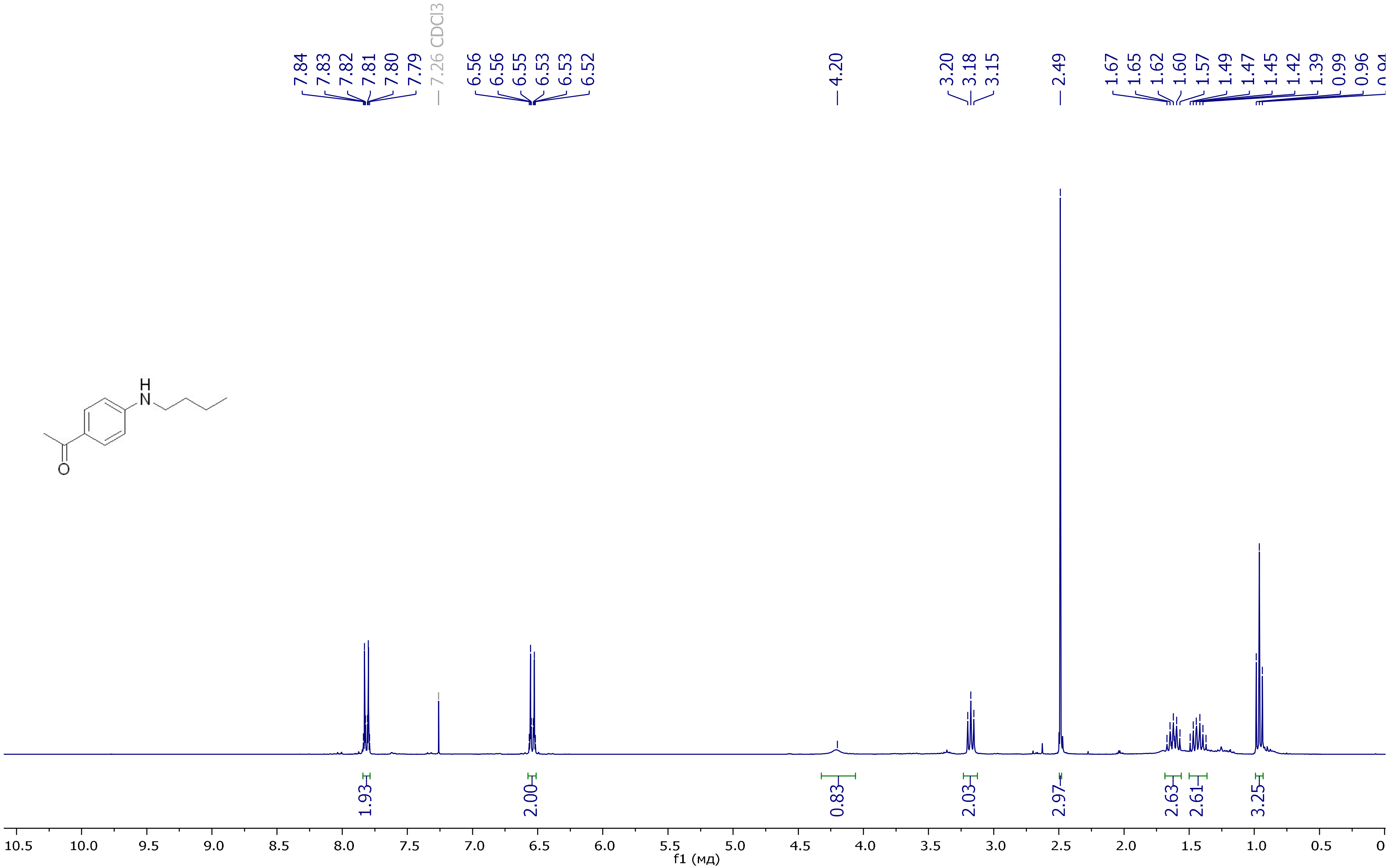

$^{13}C\{^{1}H\}$ NMR of 1-(4-(butylamino)phenyl)ethan-1-one (75 MHz, $CDCl_3$) (2e)

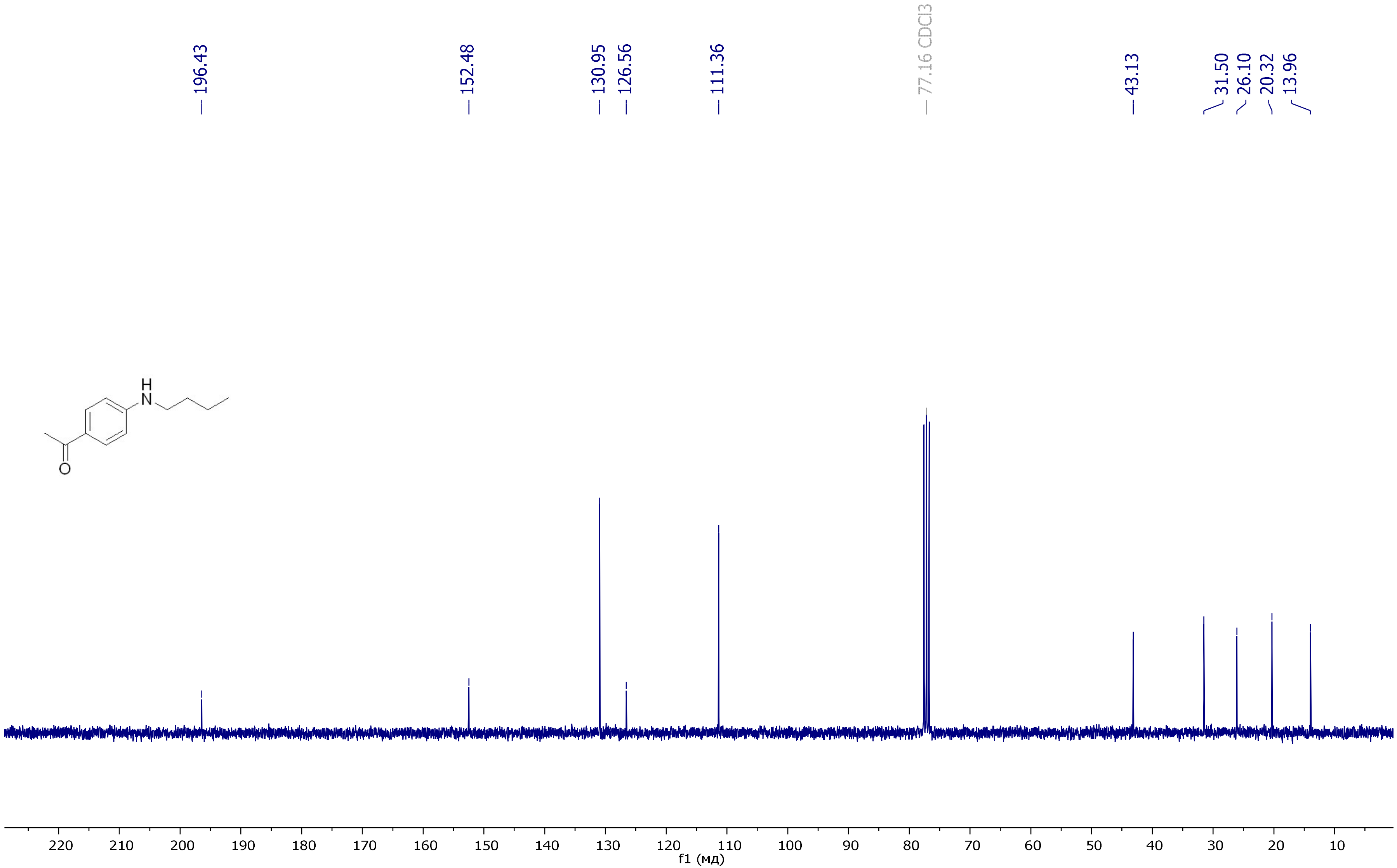

^{1}H NMR of methyl 4-(butylamino)benzoate (300 MHz, $CDCl_3$) (2f)

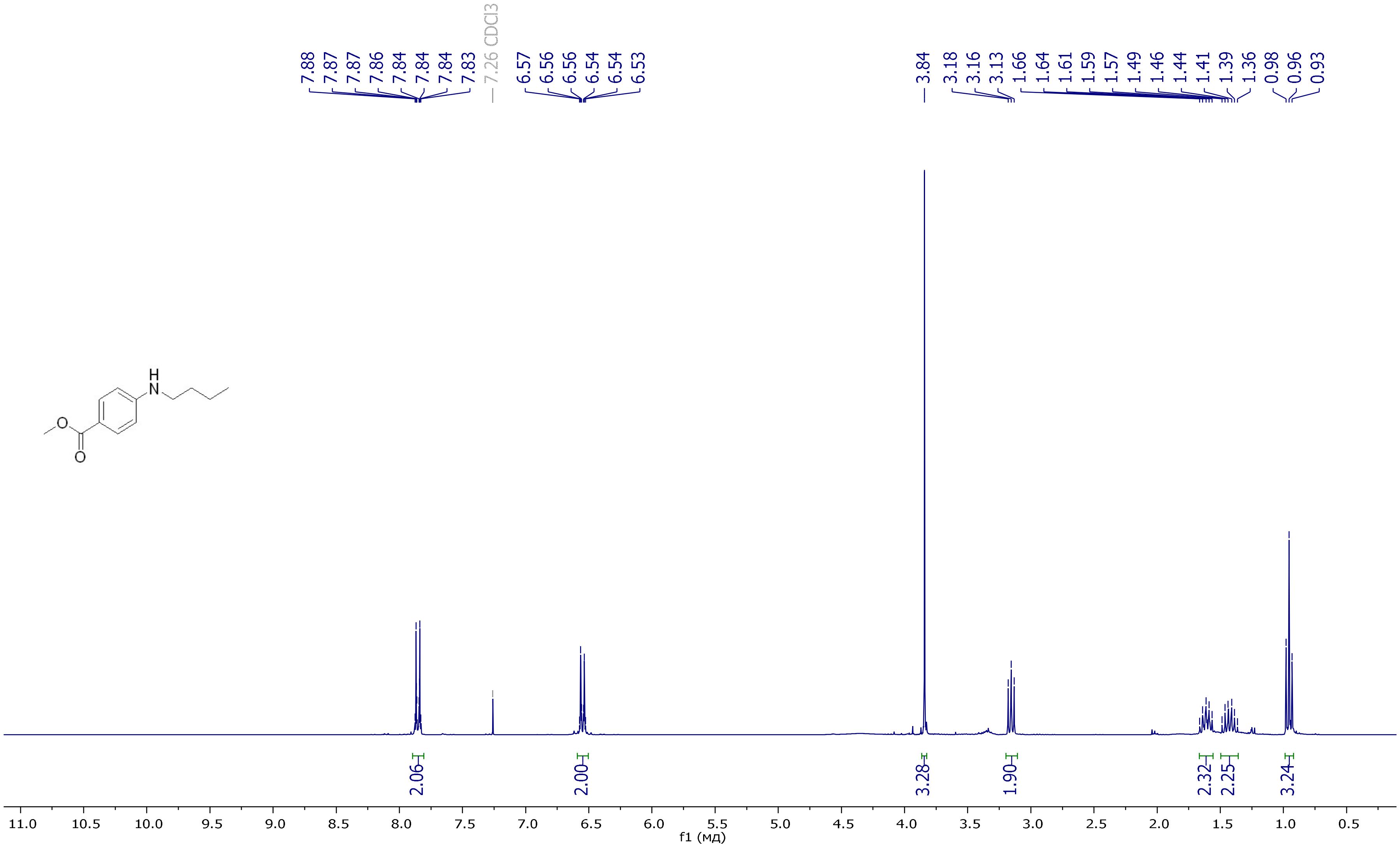

$^{13}C\{^{1}H\}$ NMR of methyl 4-(butylamino)benzoate (75 MHz, $CDCl_3$) (2f)

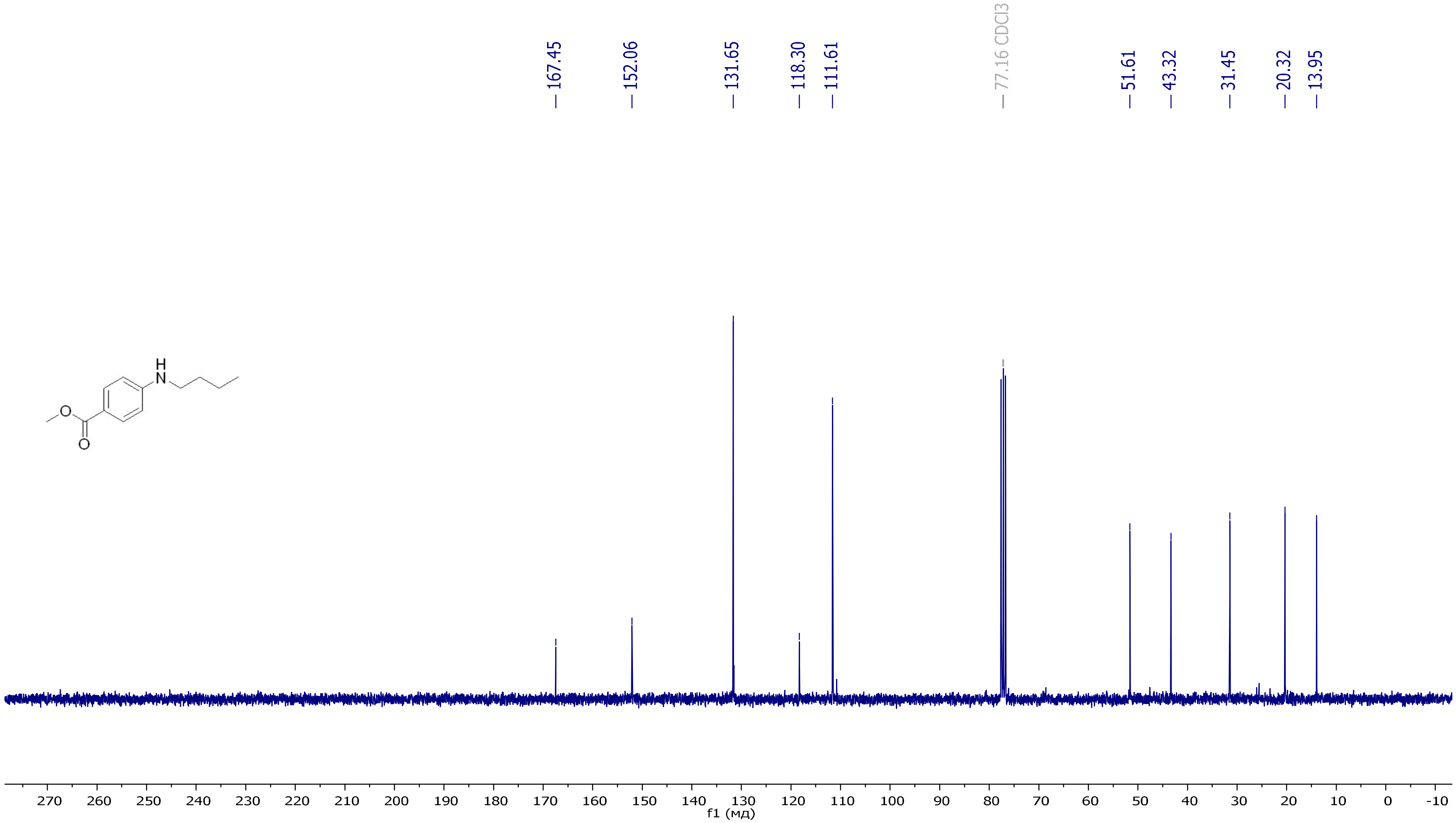

^{1}H NMR of N-butyl-4-((trifluoromethyl)thio)aniline (300 MHz, $CDCl_3$) (2g)

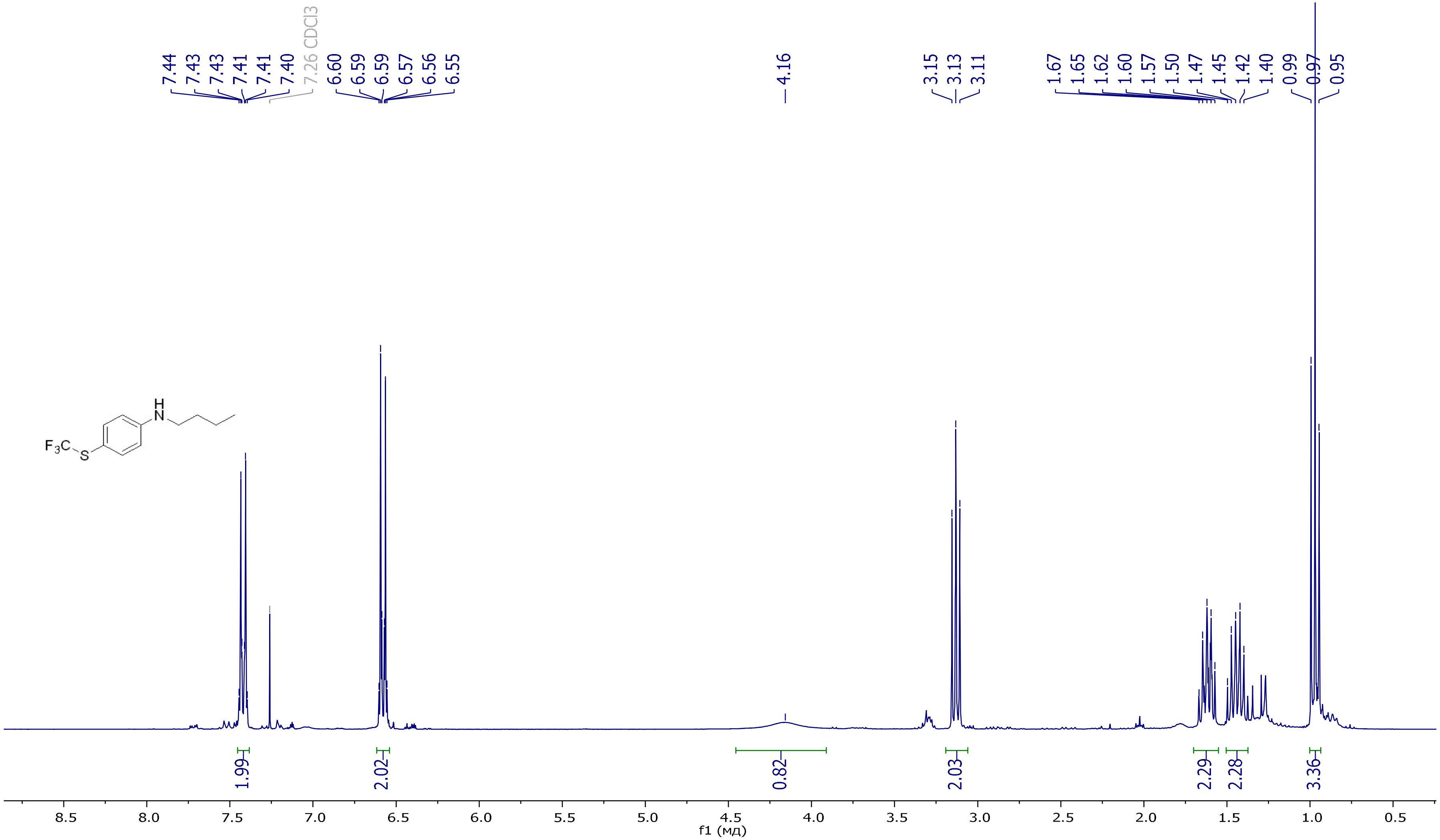

$^{13}C\{^{1}H\}$ NMR of N-butyl-4-((trifluoromethyl)thio)aniline (75 MHz, $CDCl_3$) (2g)

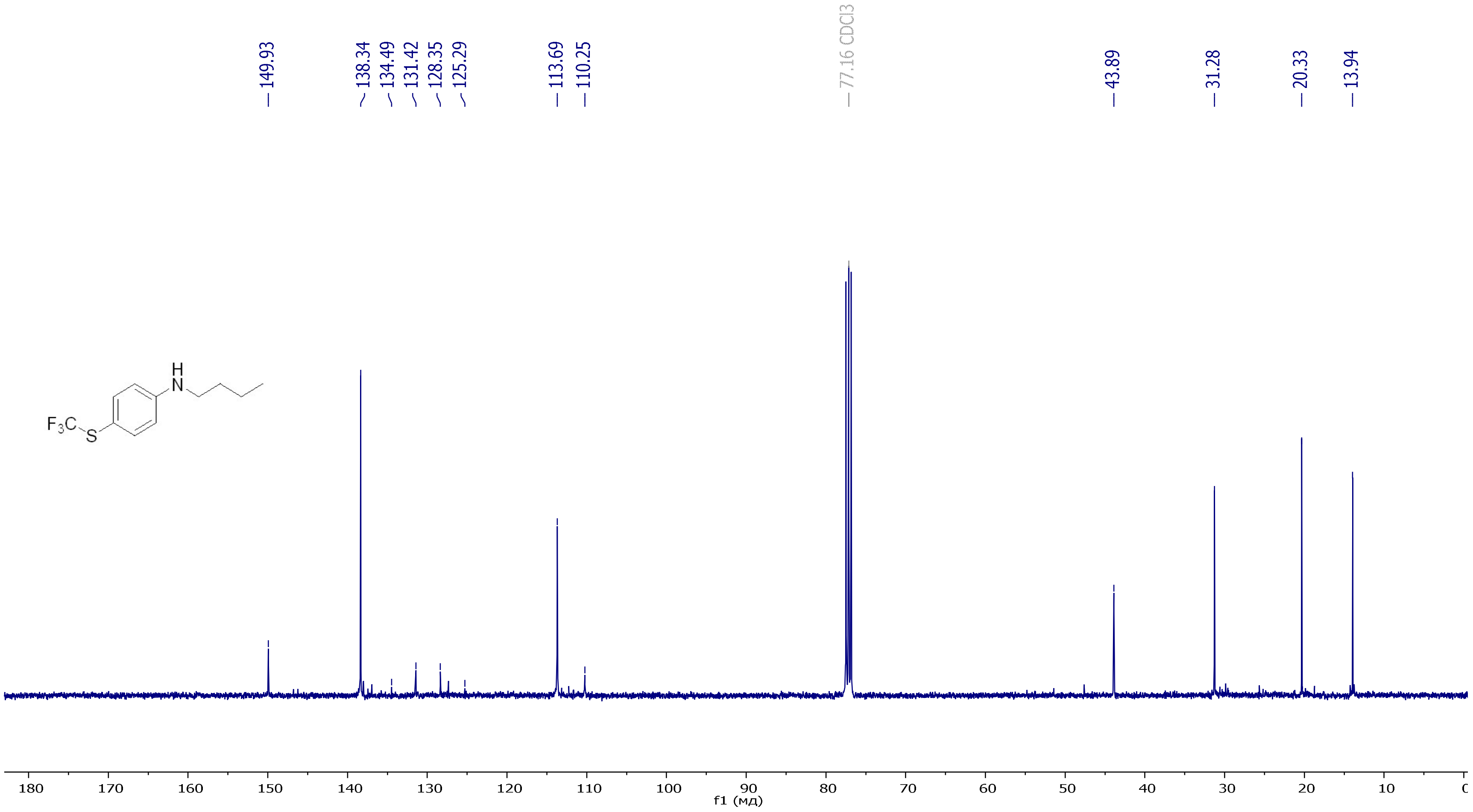

^{19}F NMR N-butyl-4-((trifluoromethyl)thio)aniline (282 MHz, $CDCl_3$) (2g)

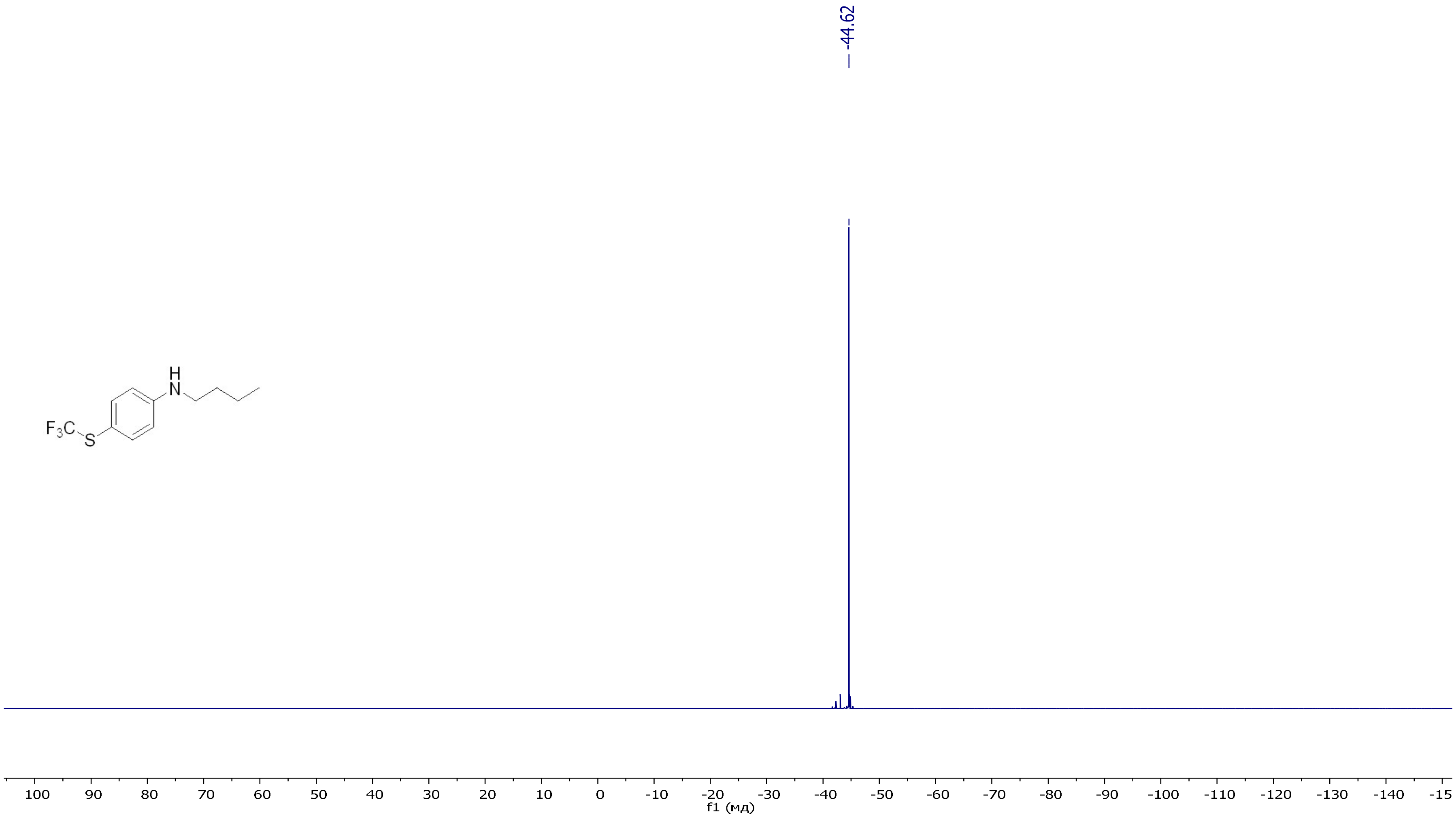

^{1}H NMR of N-butyl-2-(trifluoromethyl)aniline (300 MHz, $CDCl_3$) (2i)

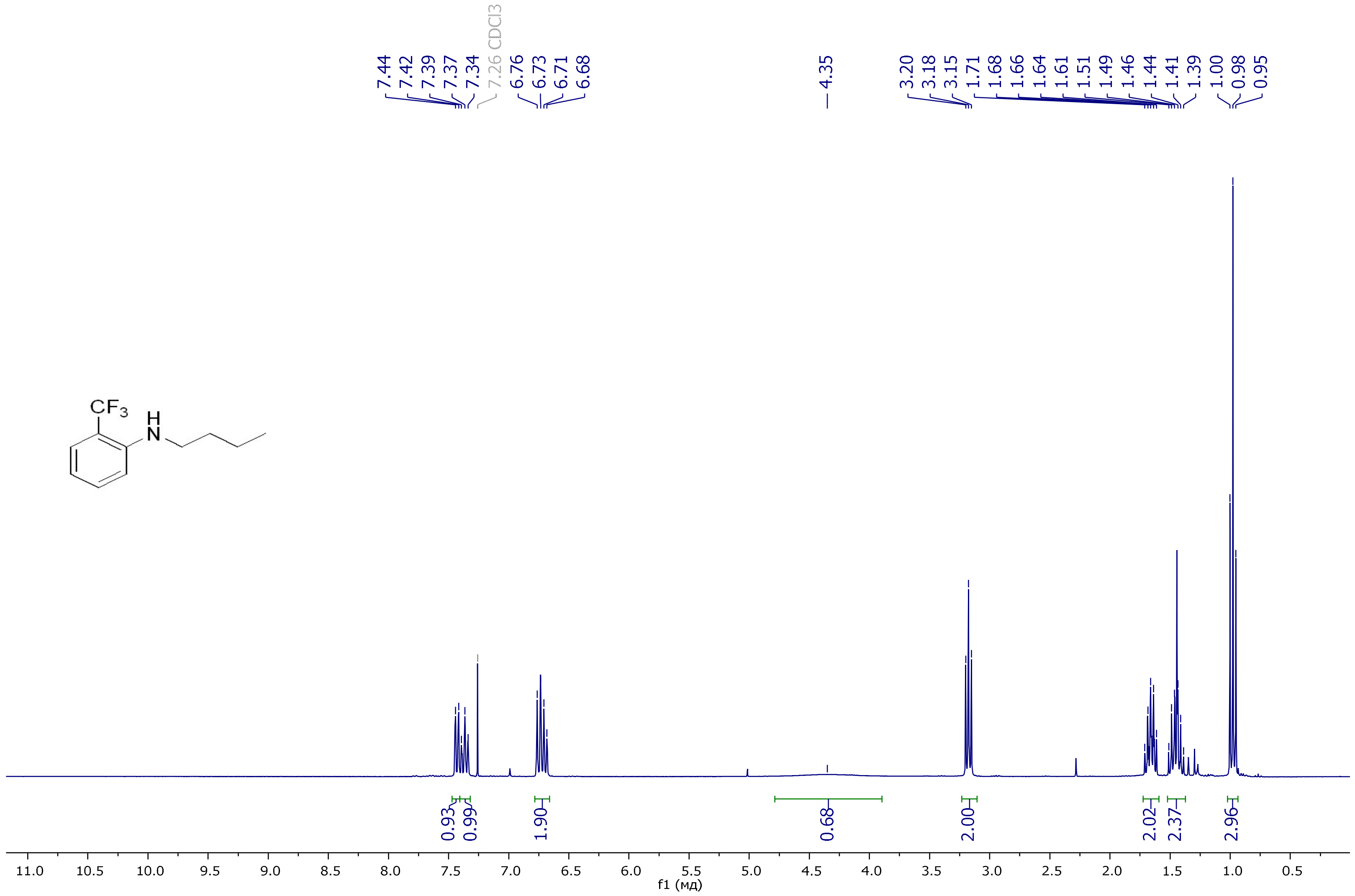

$^{13}C\{^{1}H\}$ NMR of N-butyl-2-(trifluoromethyl)aniline (75 MHz, $CDCl_3$) (2i)

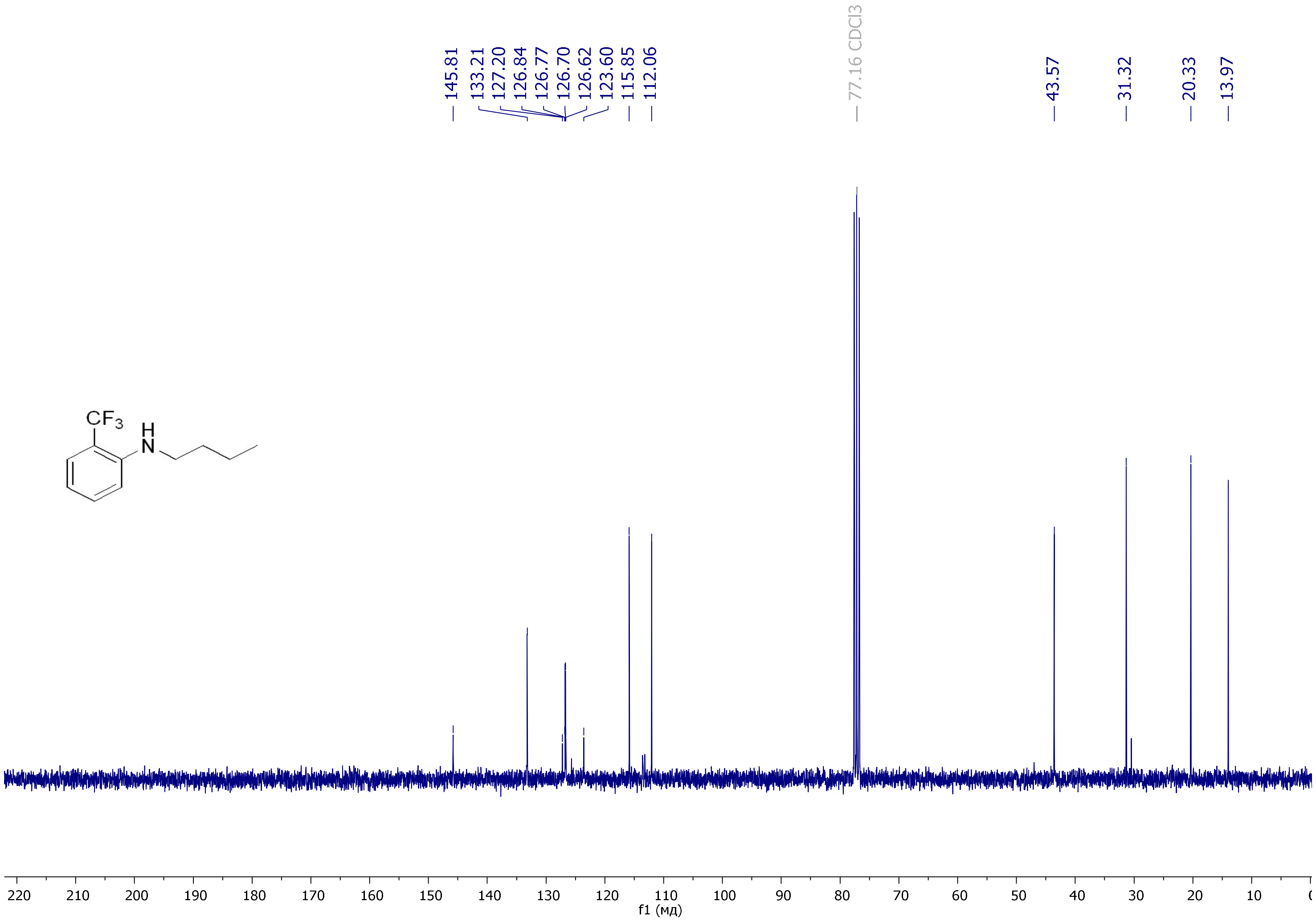

^{19}F NMR N-butyl-2-(trifluoromethyl)aniline (282 MHz, $CDCl_3$) (2i)

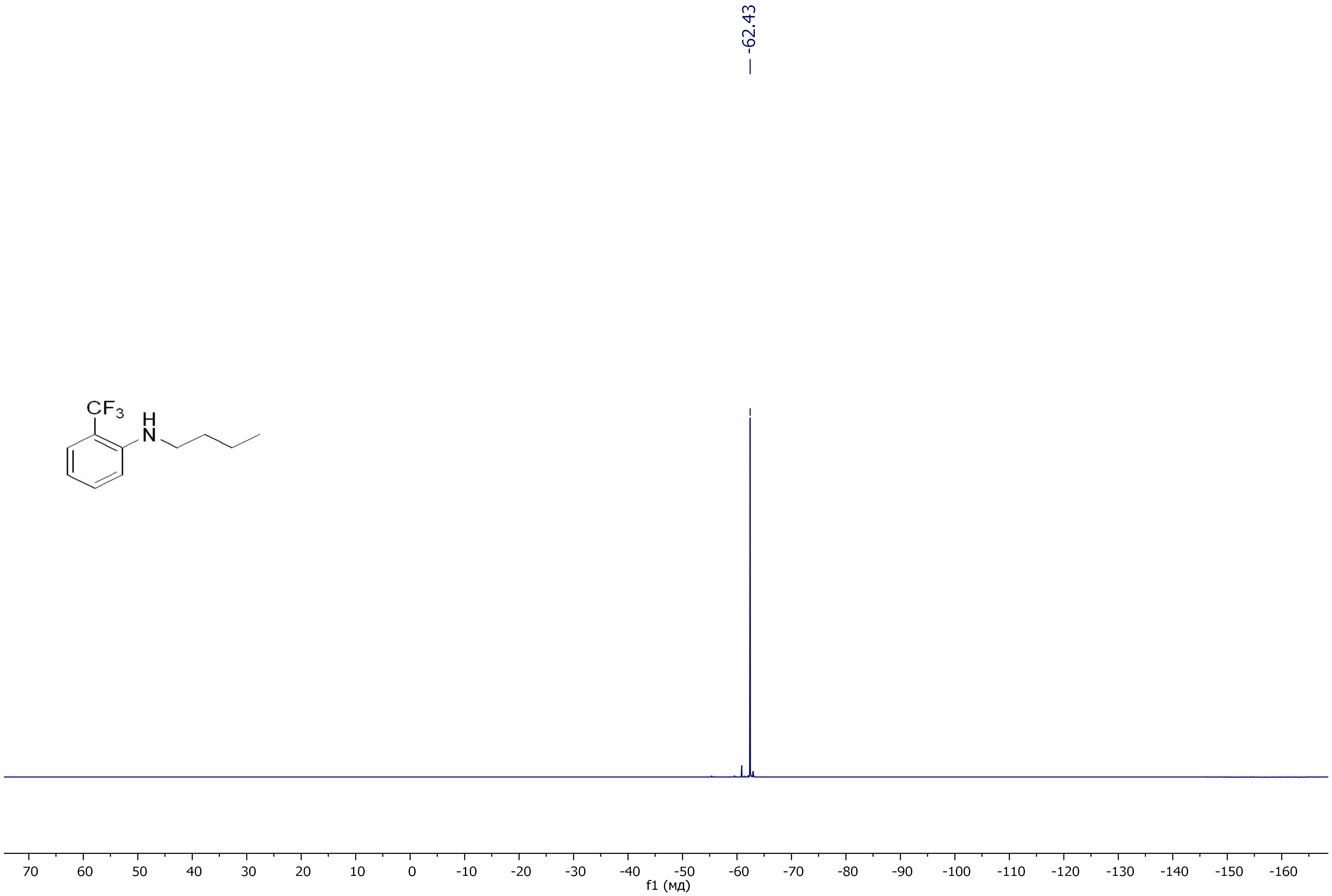

^{1}H NMR of N-butylaniline (300 MHz, CDCl$_3$) (2j)

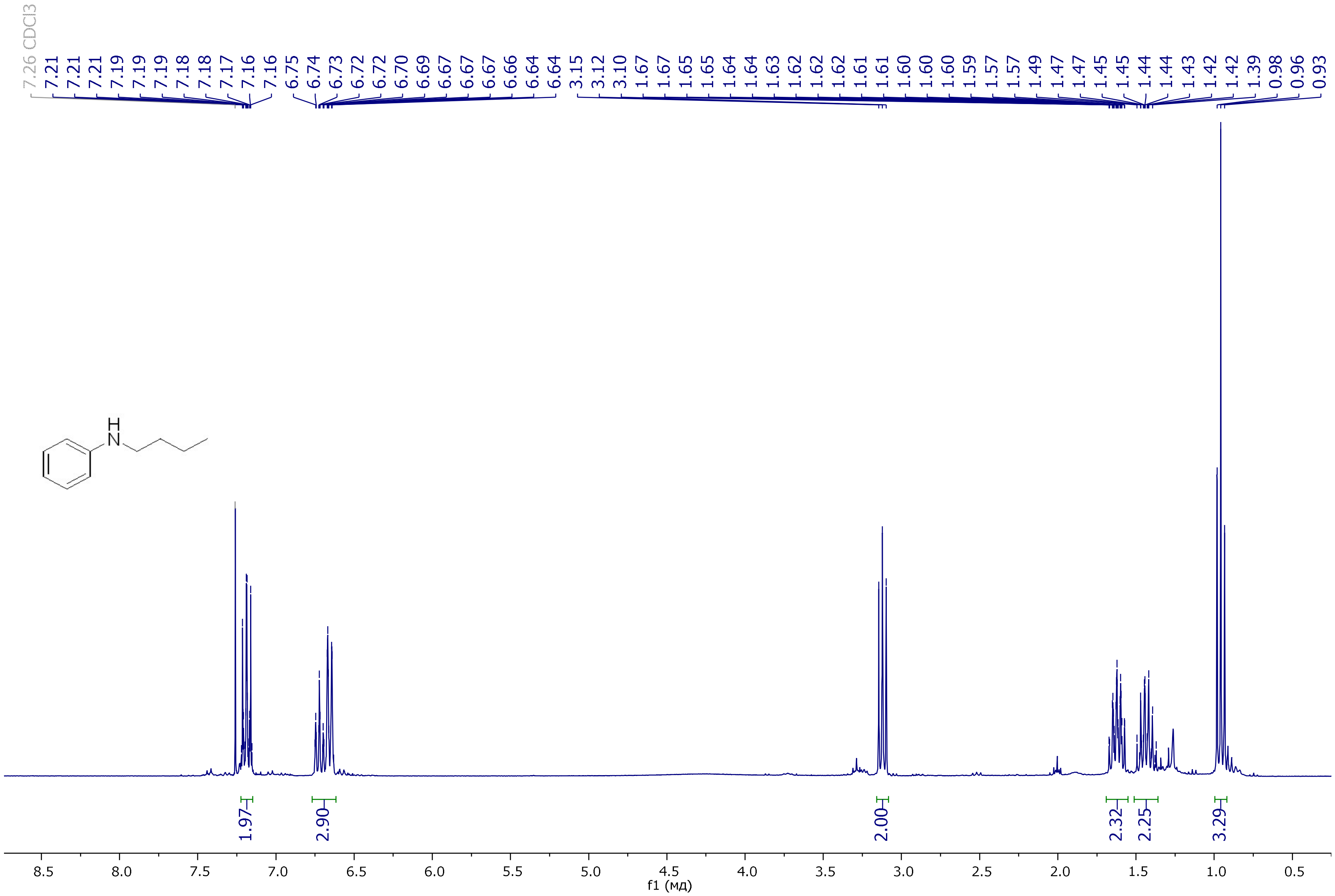

$^{13}C\{^1H\}$ NMR of N-butylaniline (75 MHz, $CDCl_3$) (2j)

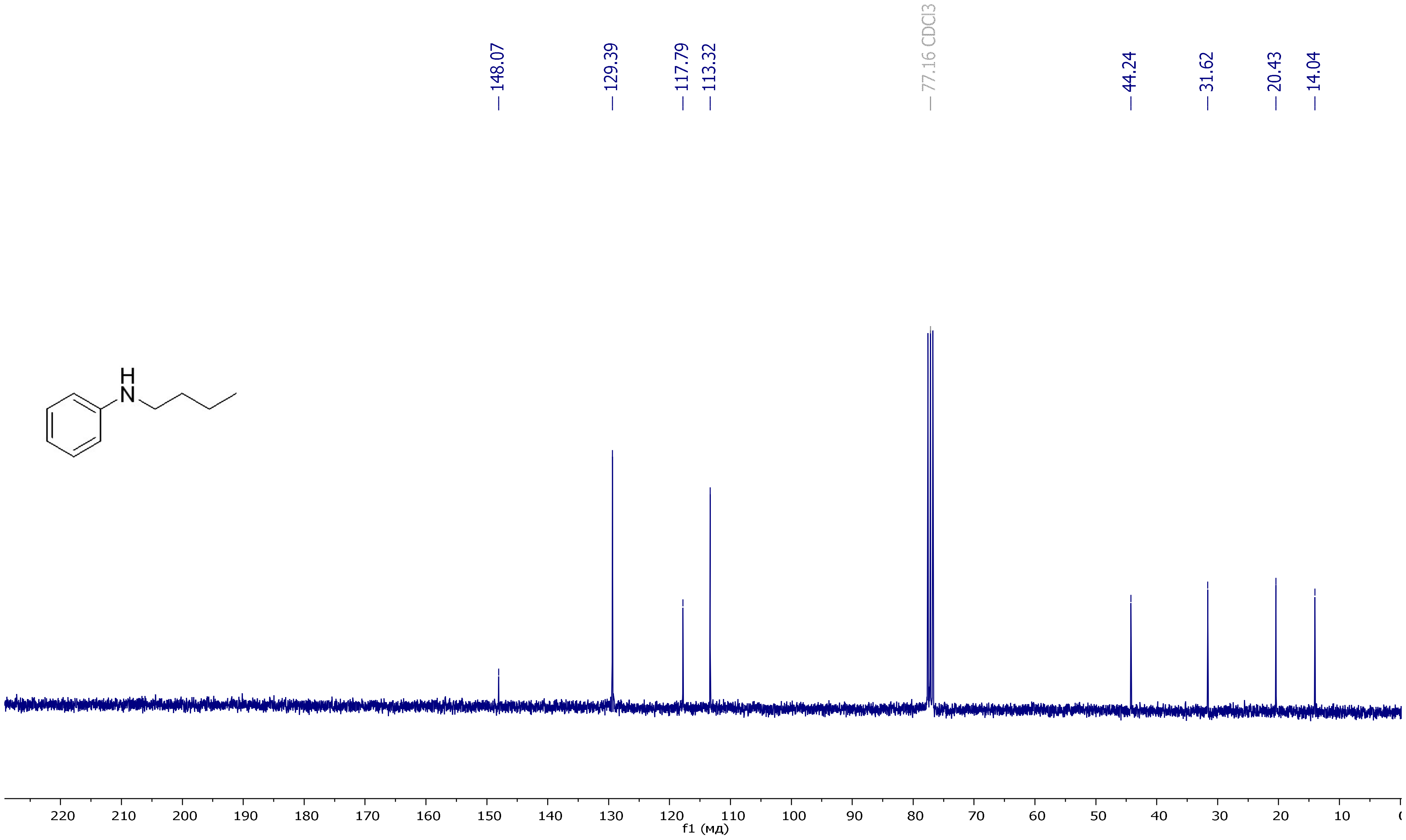

^{1}H NMR of N-butylnaphthalen-1-amine (400 MHz, $CDCl_3$) (2k)

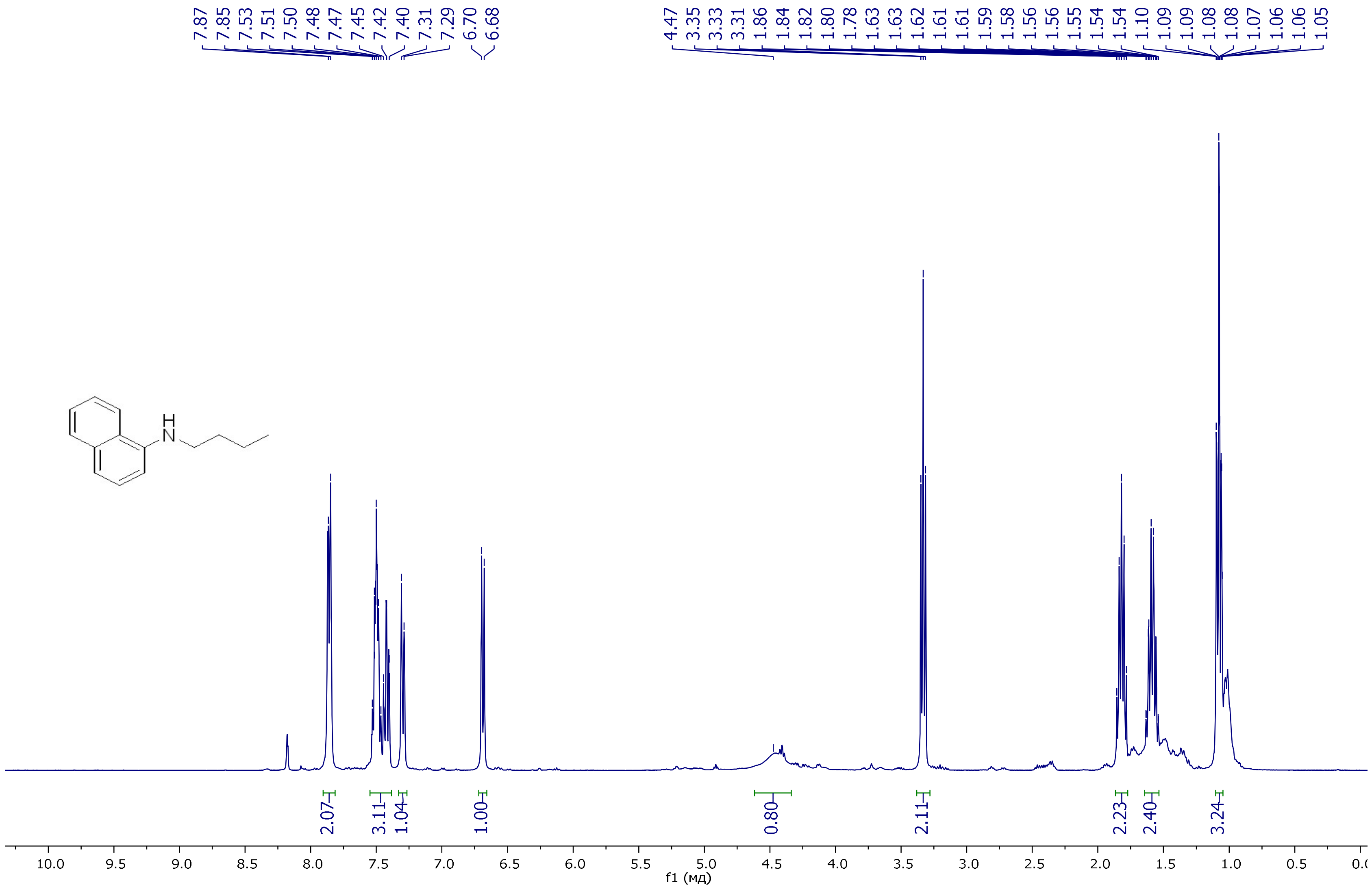

$^{13}C\{^1H\}$ NMR of N-butylnaphthalen-1-amine (101 MHz, $CDCl_3$) (2k)

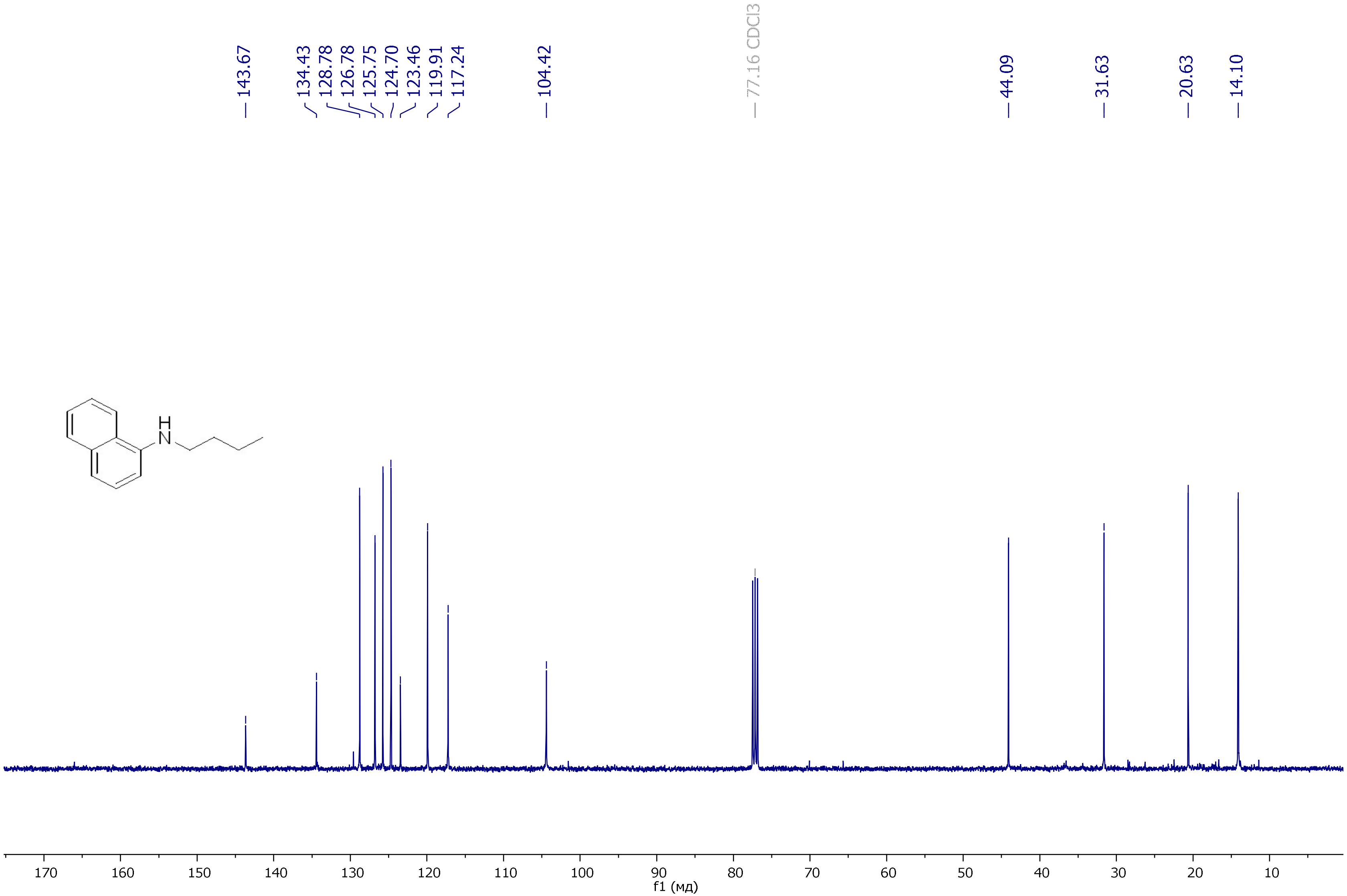

^{1}H NMR of N,N-dibutyl-4-methoxyaniline (400 MHz, $CDCl_3$) (3a)

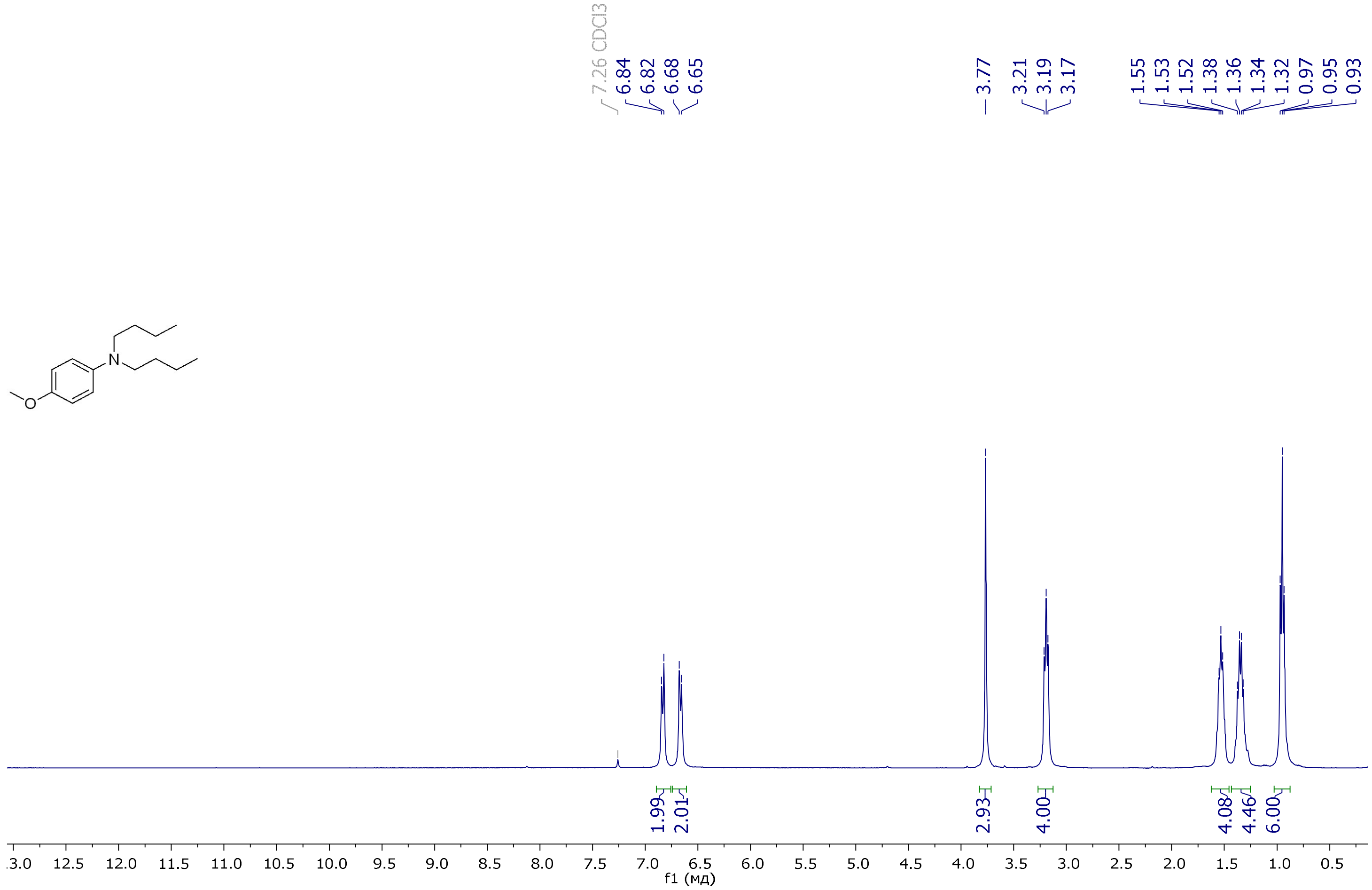

$^{13}C\{^{1}H\}$ NMR of N,N-dibutyl-4-methoxyaniline (101 MHz, $CDCl_3$) (3a)

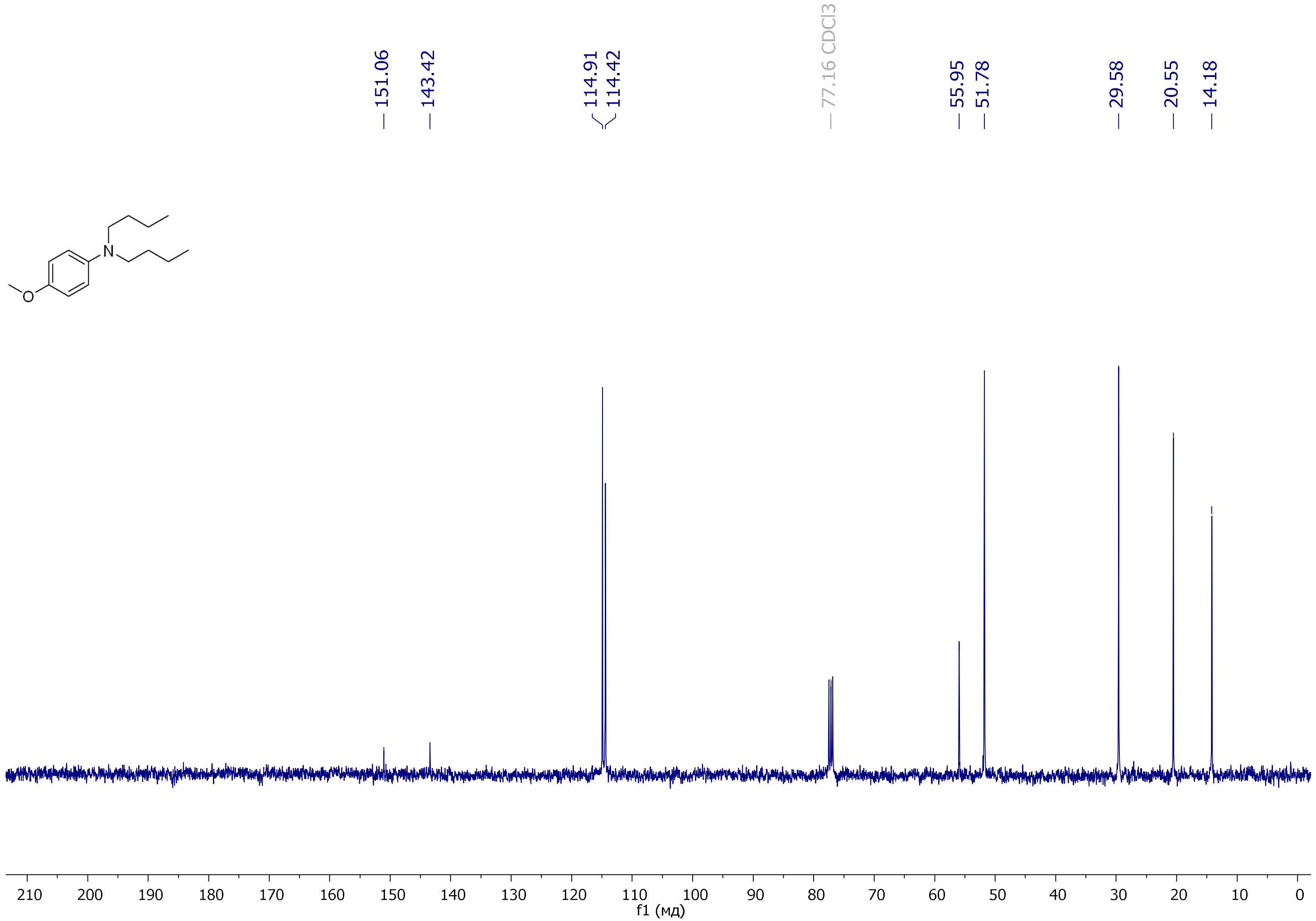

^{1}H NMR of N,N-dibutyl-4-methylaniline (400 MHz, $CDCl_3$) (3d)

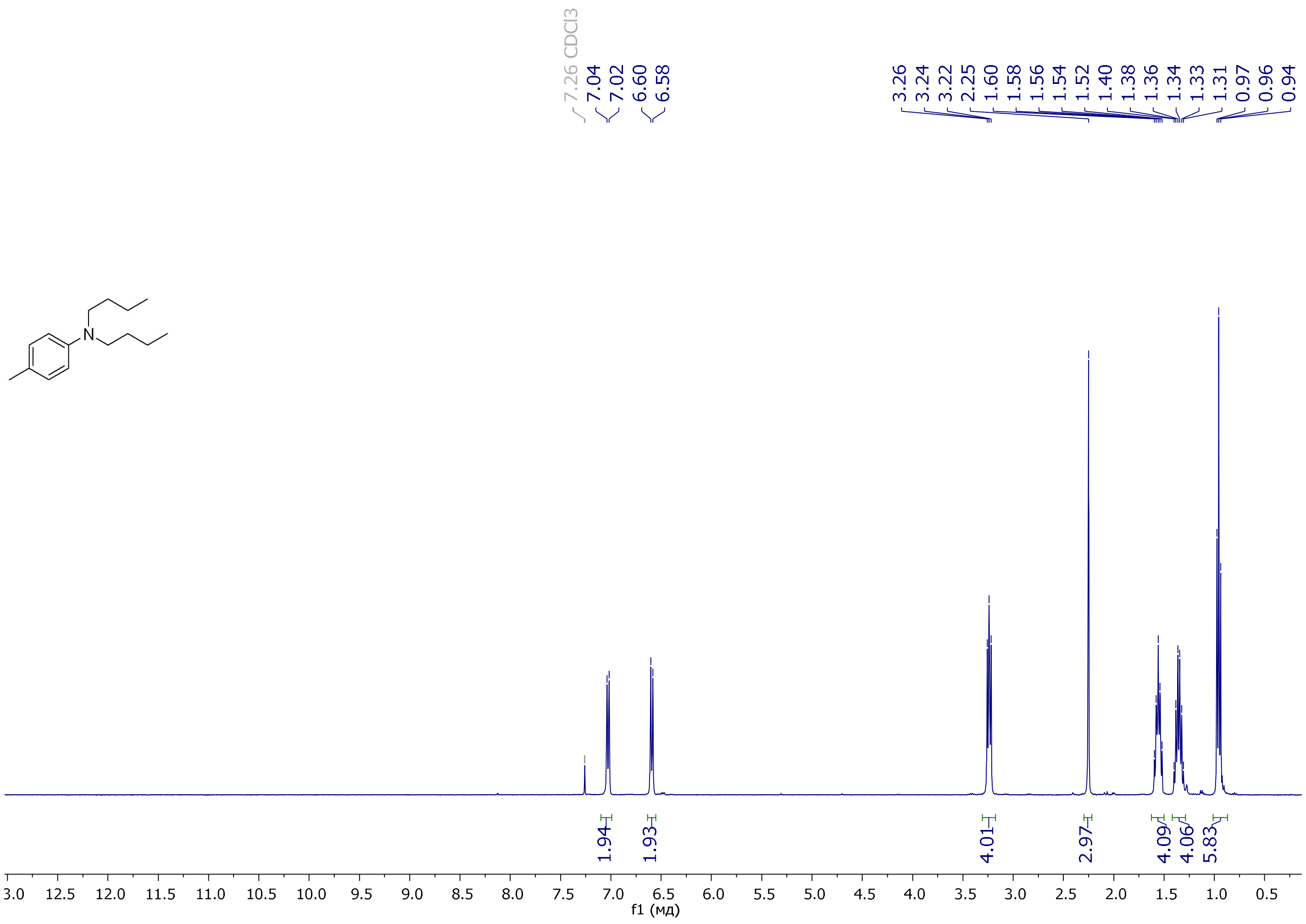

$^{13}C\{^{1}H\}$ NMR of N,N-dibutyl-4-methylaniline (101 MHz, $CDCl_3$) (3d)

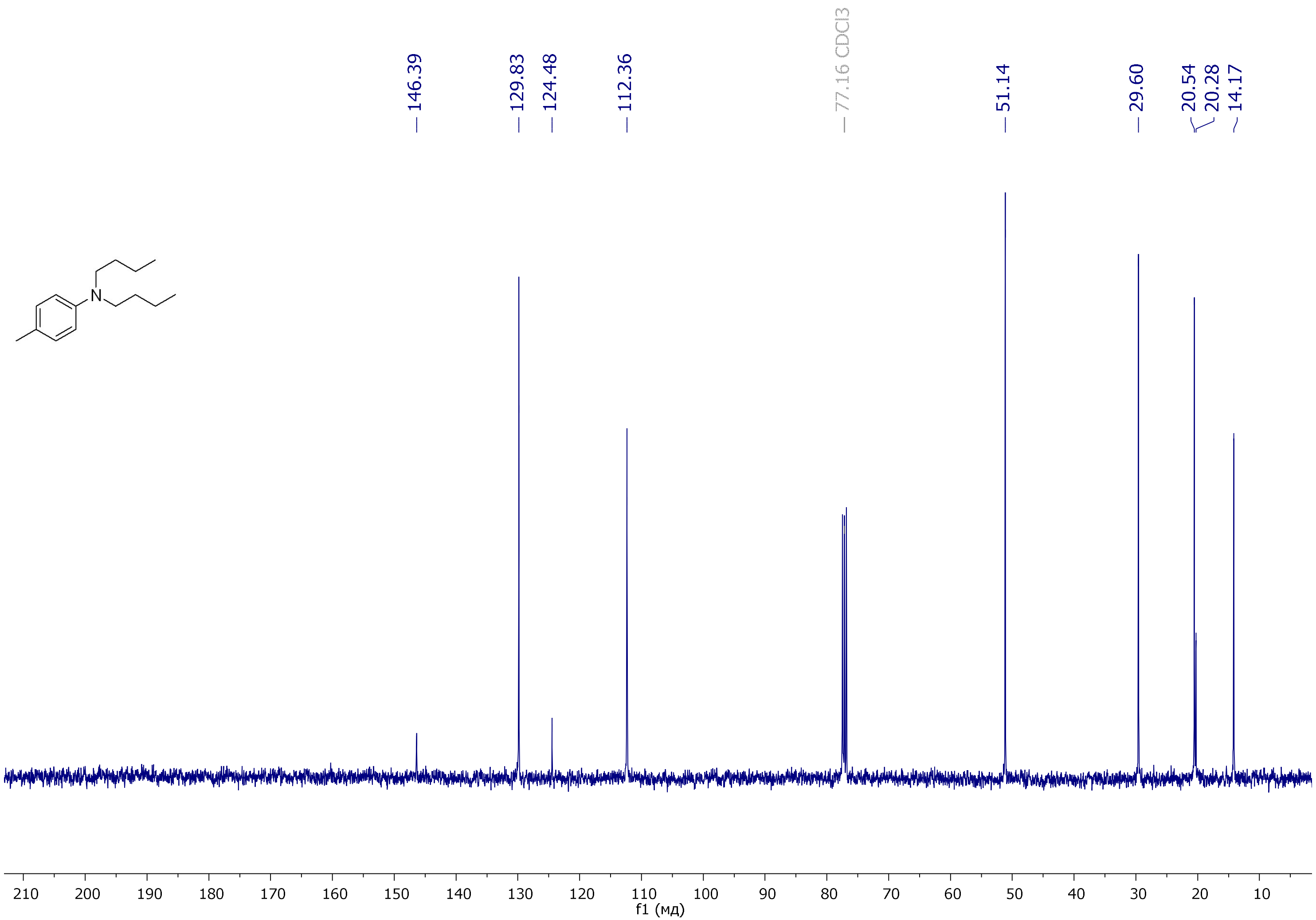

^{1}H NMR of N,N-dibutylaniline (400 MHz, $CDCl_3$) (3j)

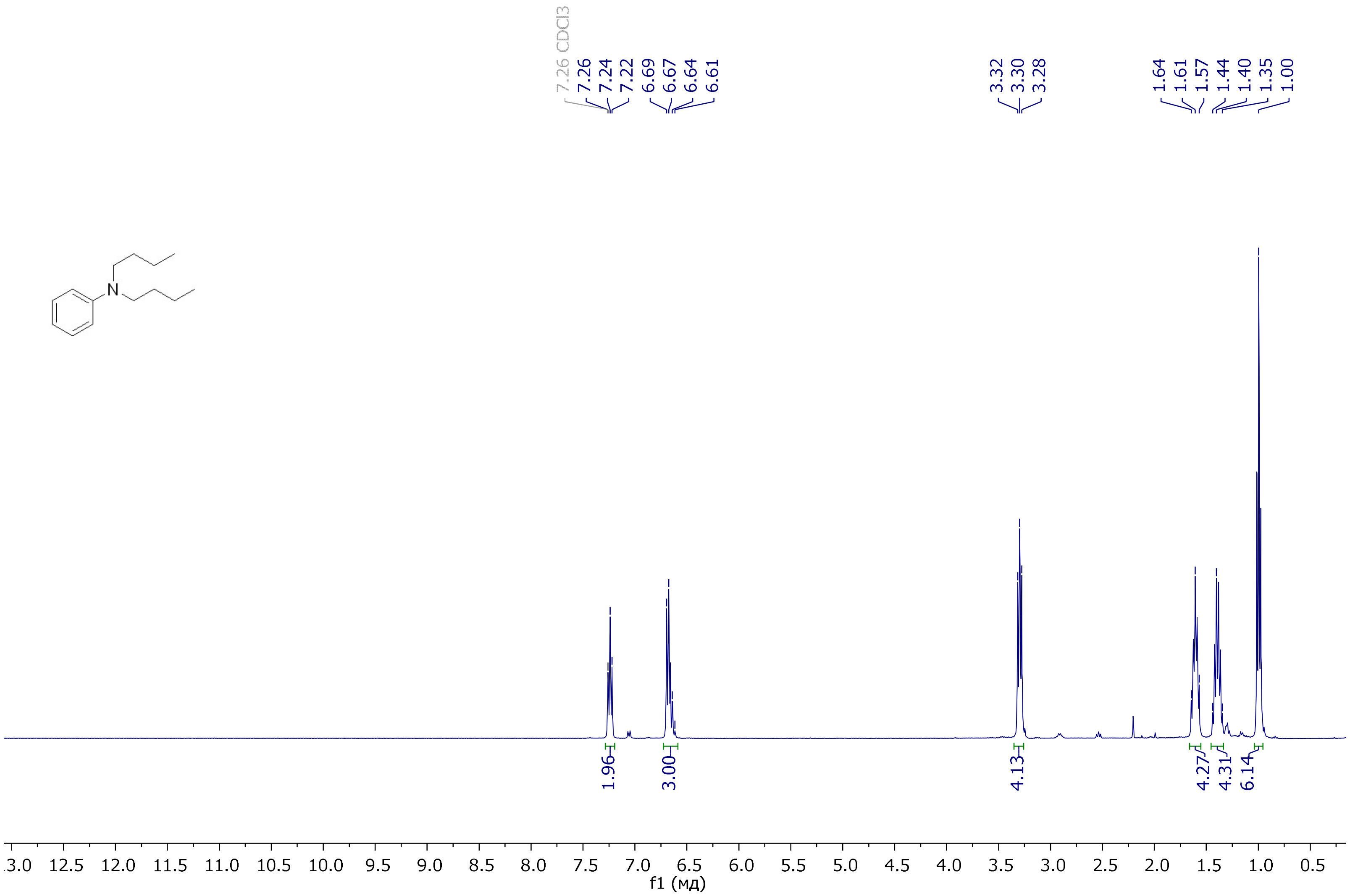

$^{13}C\{^{1}H\}$ NMR of N,N-dibutylaniline (101 MHz, $CDCl_3$) (3j)

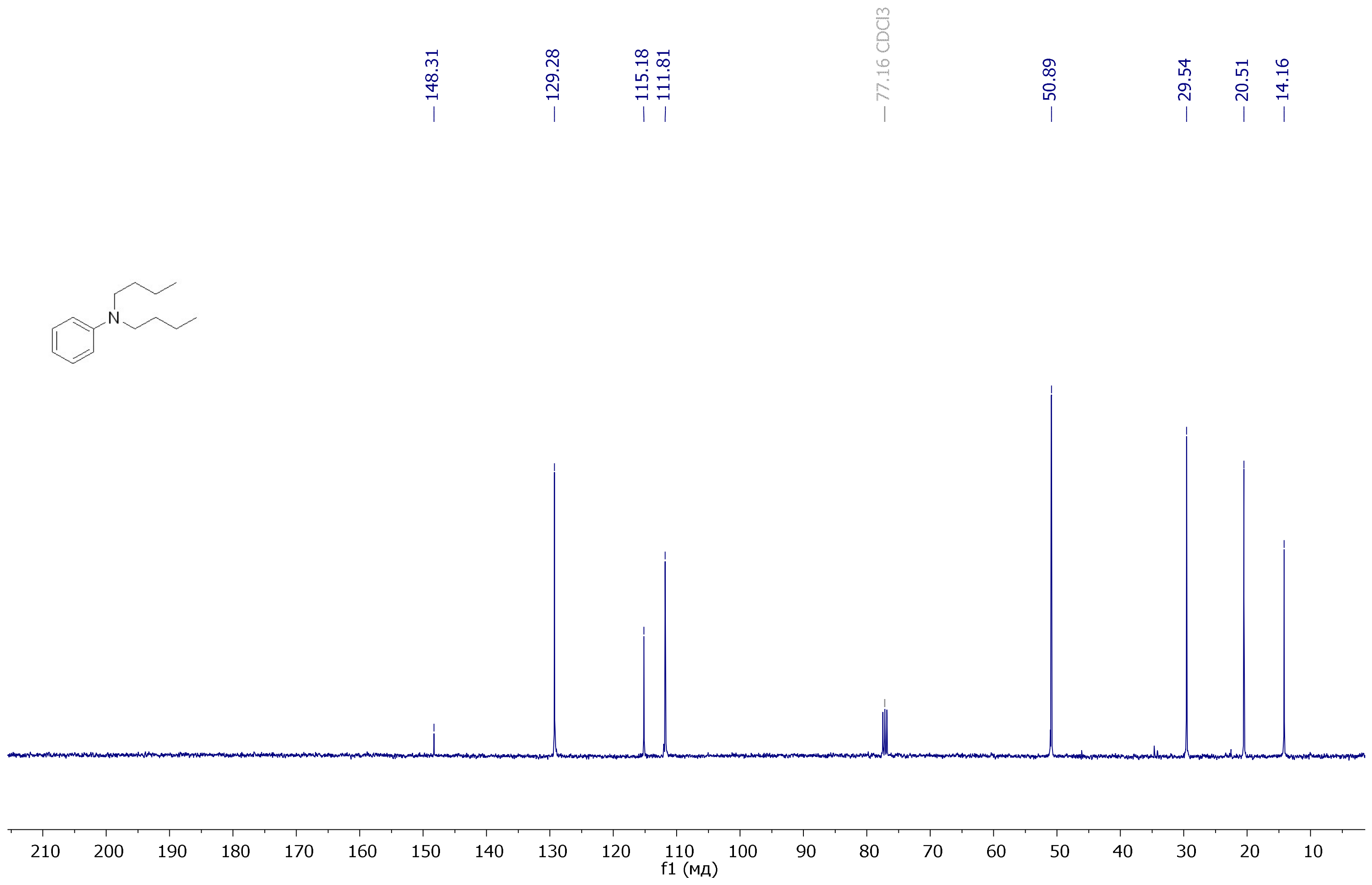

^{1}H NMR of N,N-dibutyl-3-(trifluoromethyl)aniline (300 MHz, $CDCl_3$) (3l)

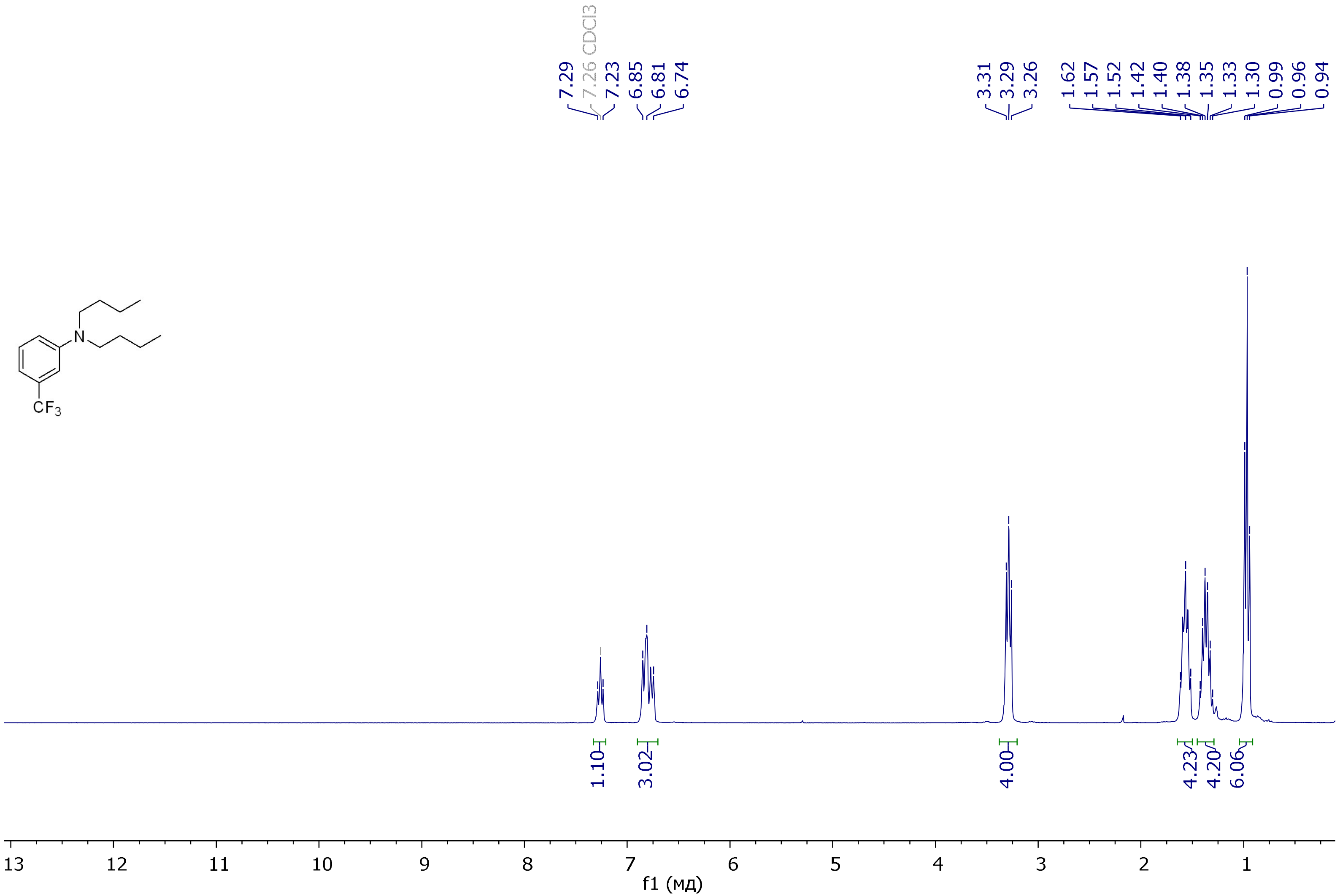

$^{13}C\{^{1}H\}$ NMR of N,N-dibutyl-3-(trifluoromethyl)aniline (101 MHz, $CDCl_3$) (3l)

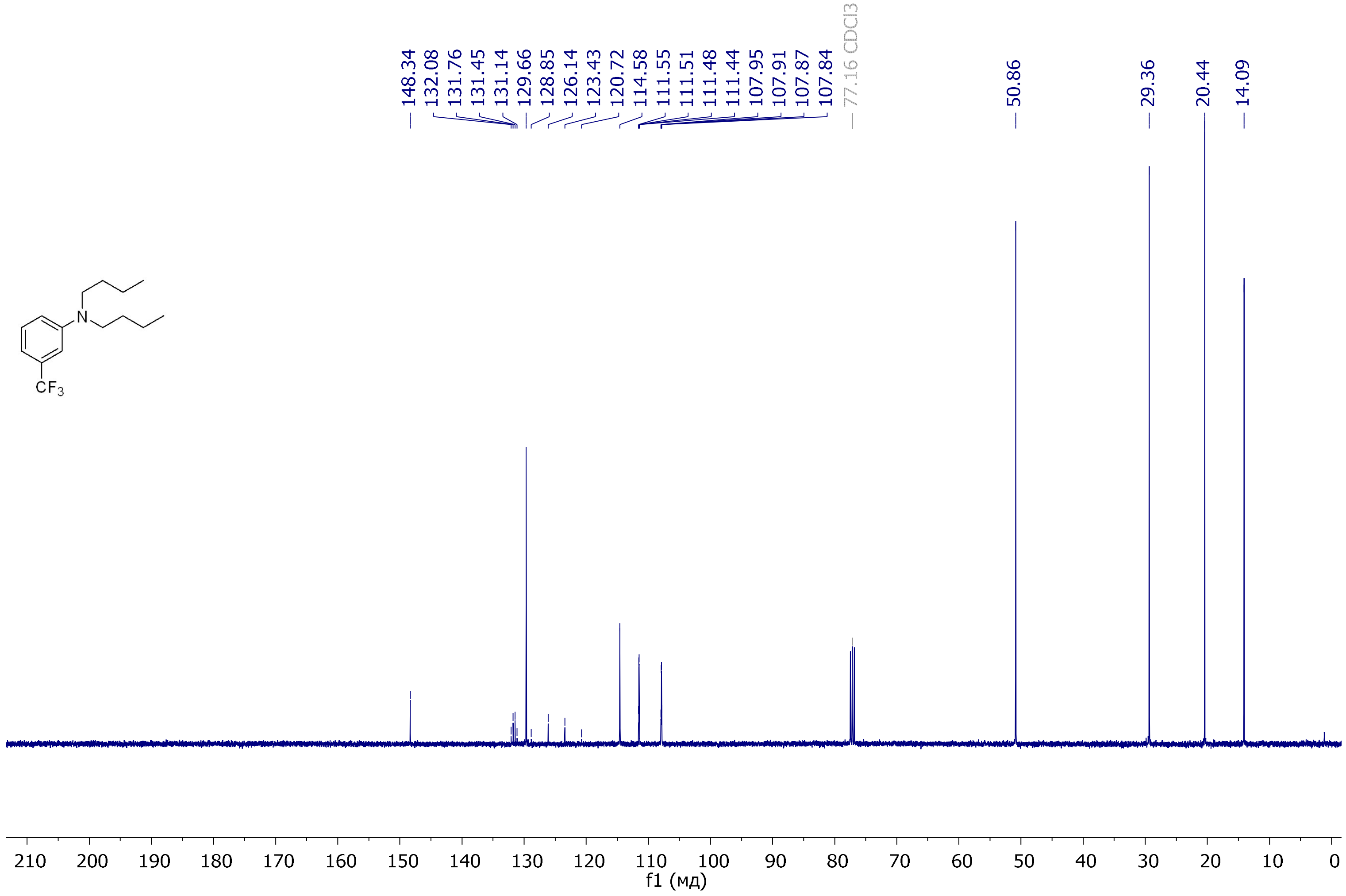

^{19}F NMR of N,N-dibutyl-3-(trifluoromethyl)aniline (376 MHz, $CDCl_3$) (3l)

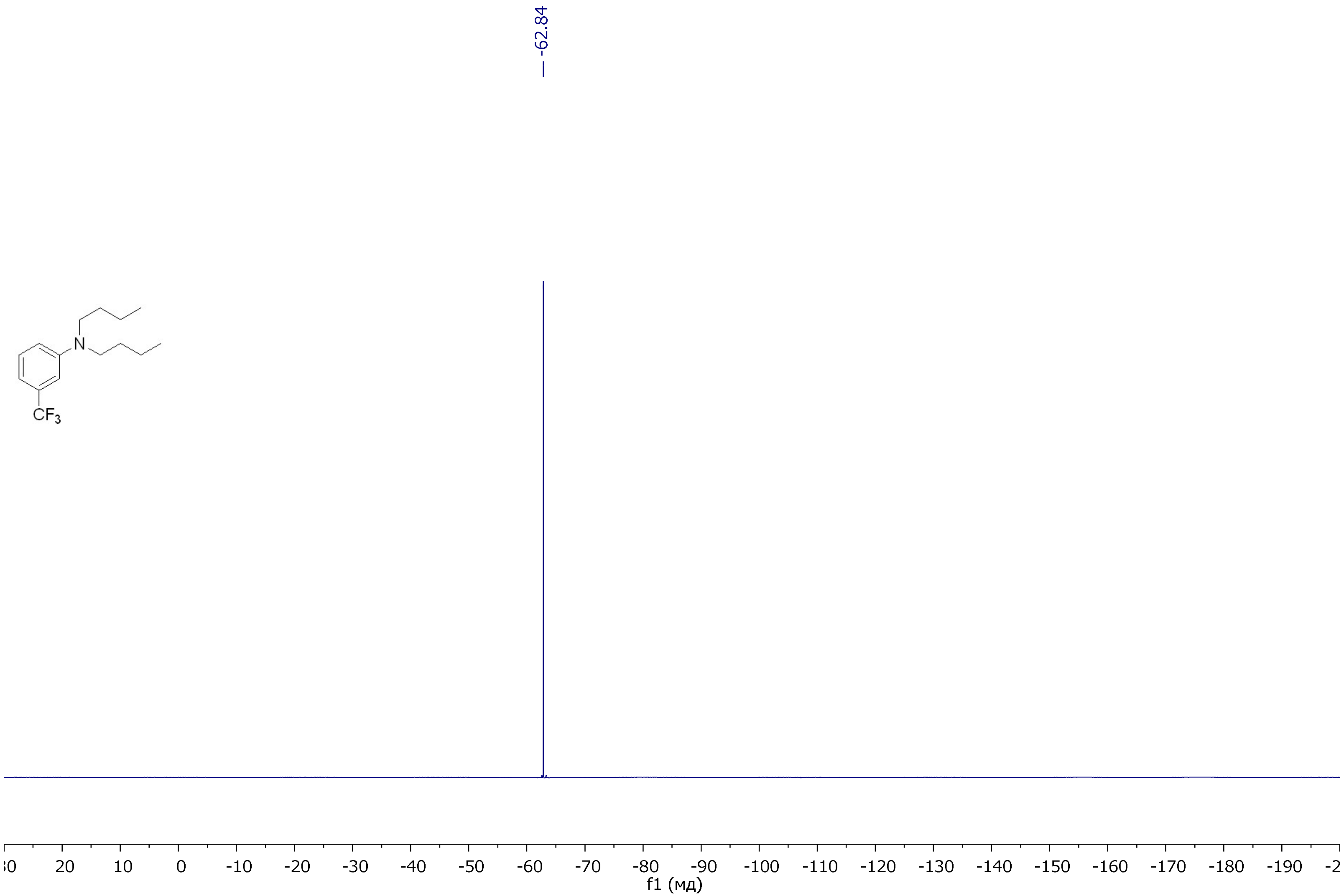

^{1}H NMR of N,N-dibutyl-4-fluoroaniline (400 MHz, $CDCl_3$) (3m)

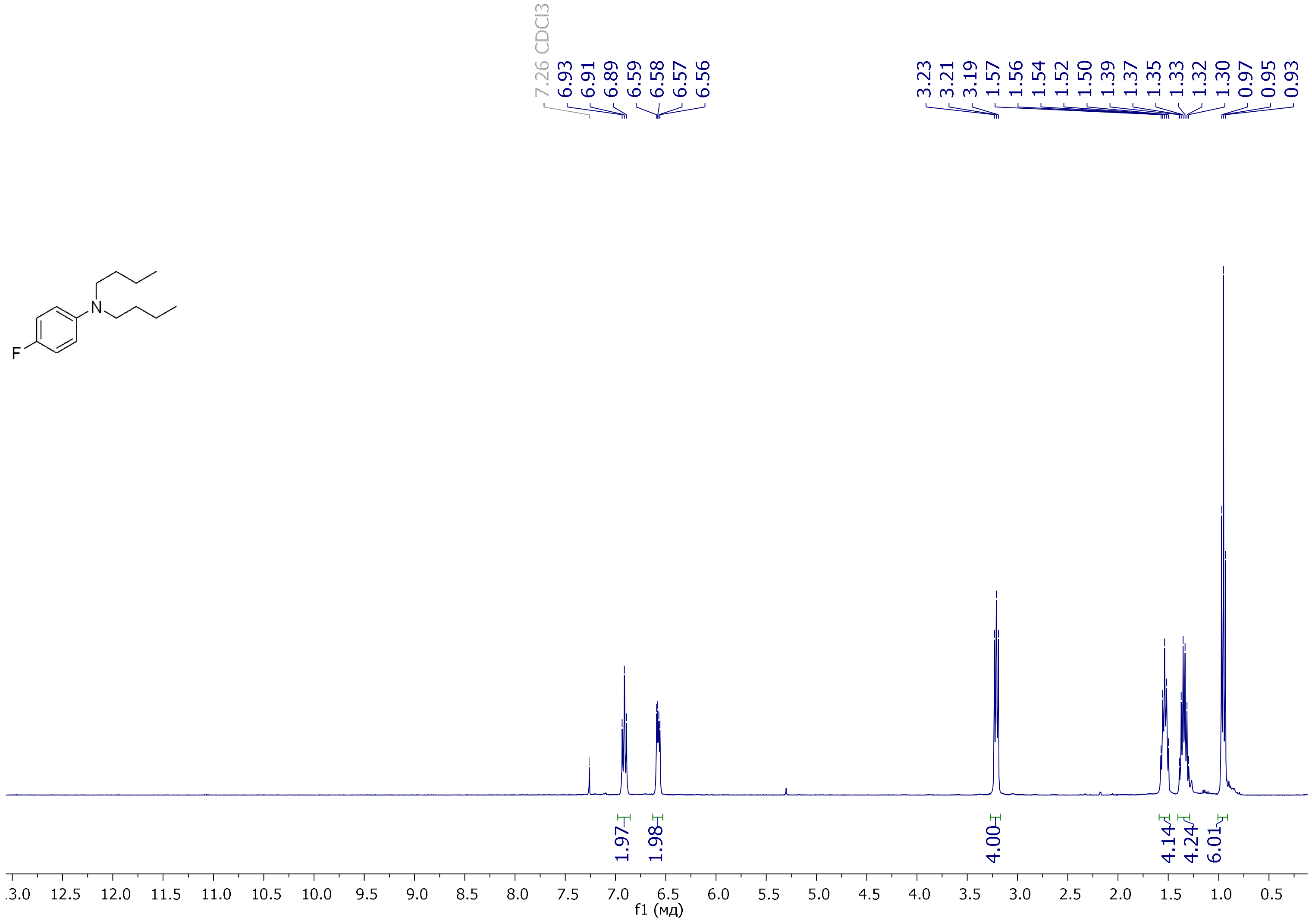

^{13}C{^{1}H} NMR of N,N-dibutyl-4-fluoroaniline (101 MHz, $CDCl_3$) (3m)

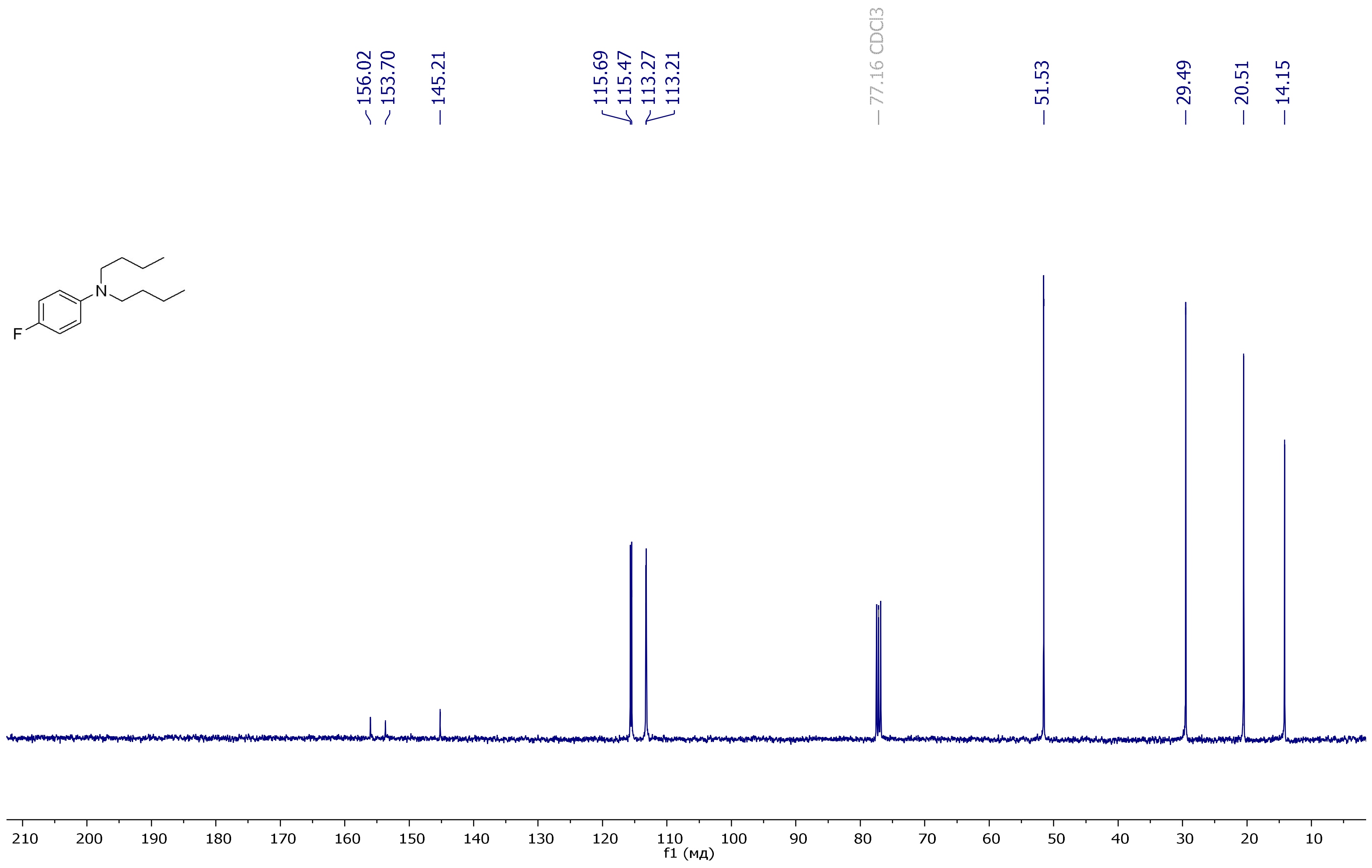

^{19}F NMR of N,N-dibutyl-4-fluoroaniline (376 MHz, $CDCl_3$) (3m)

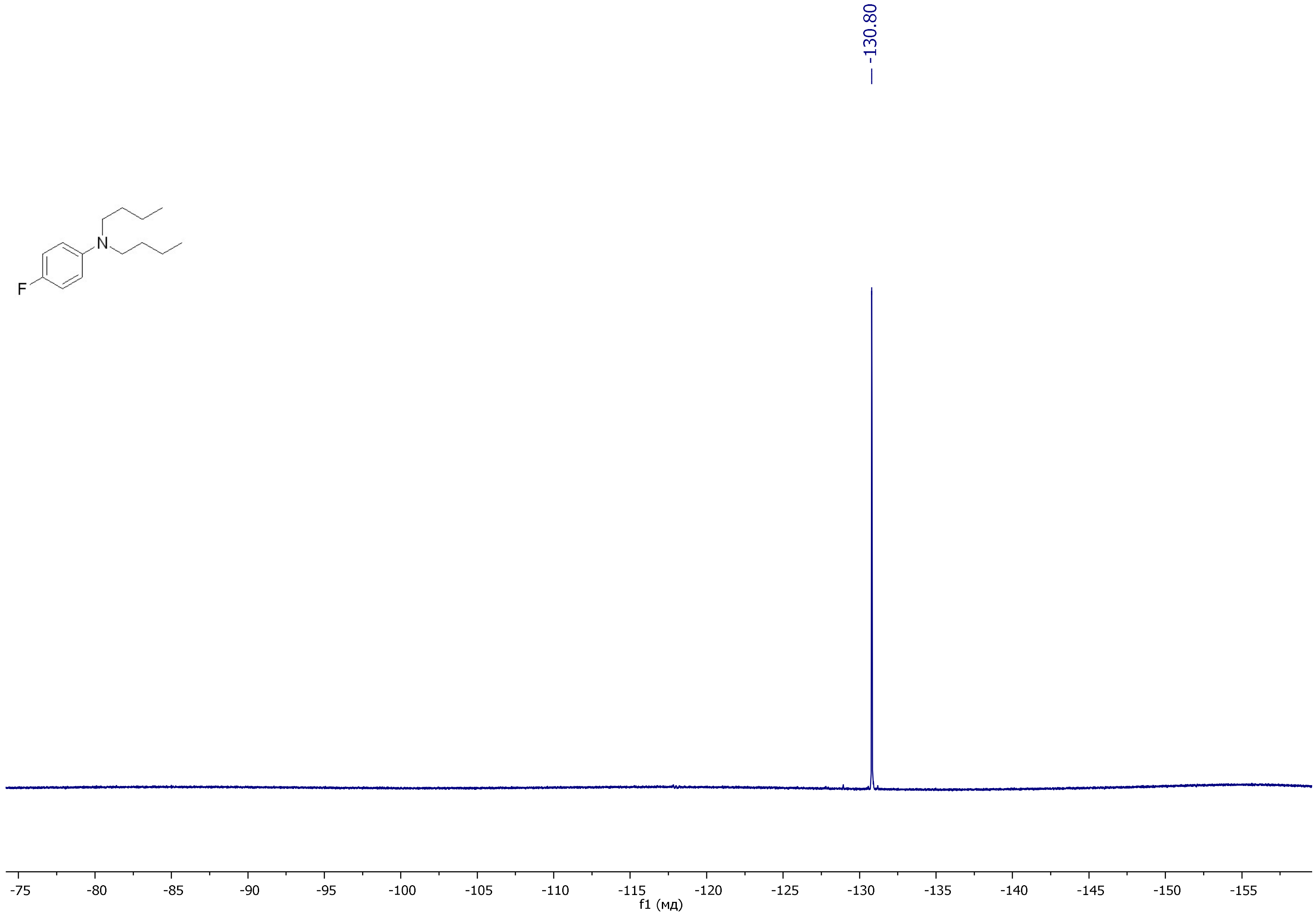

^{1}H NMR of N,N-dibutyl-2,6-dimethylaniline (400 MHz, $CDCl_3$) (3n)

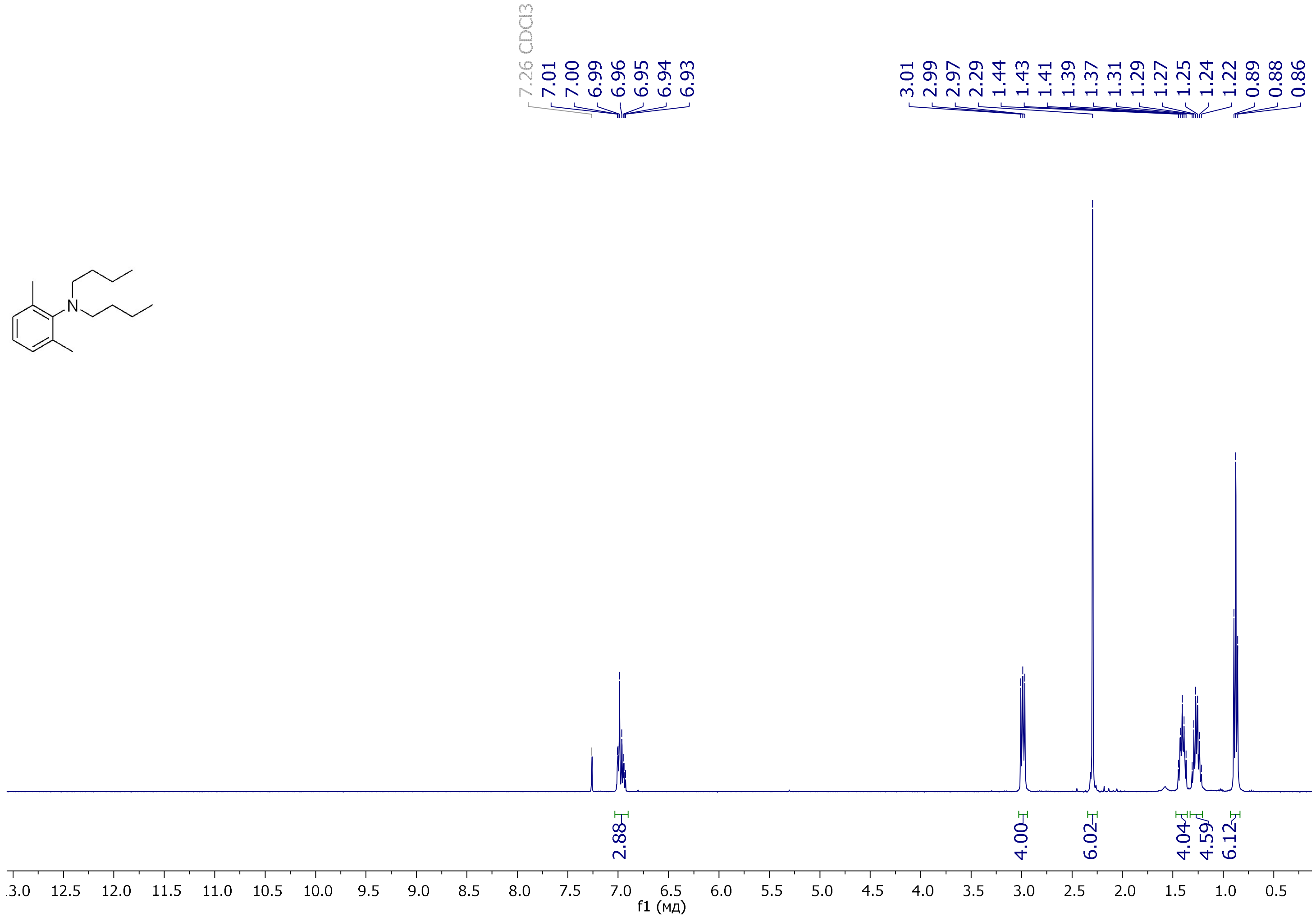

$^{13}C\{^1H\}$ NMR of N,N-dibutyl-2,6-dimethylaniline (101 MHz, $CDCl_3$) (3n)

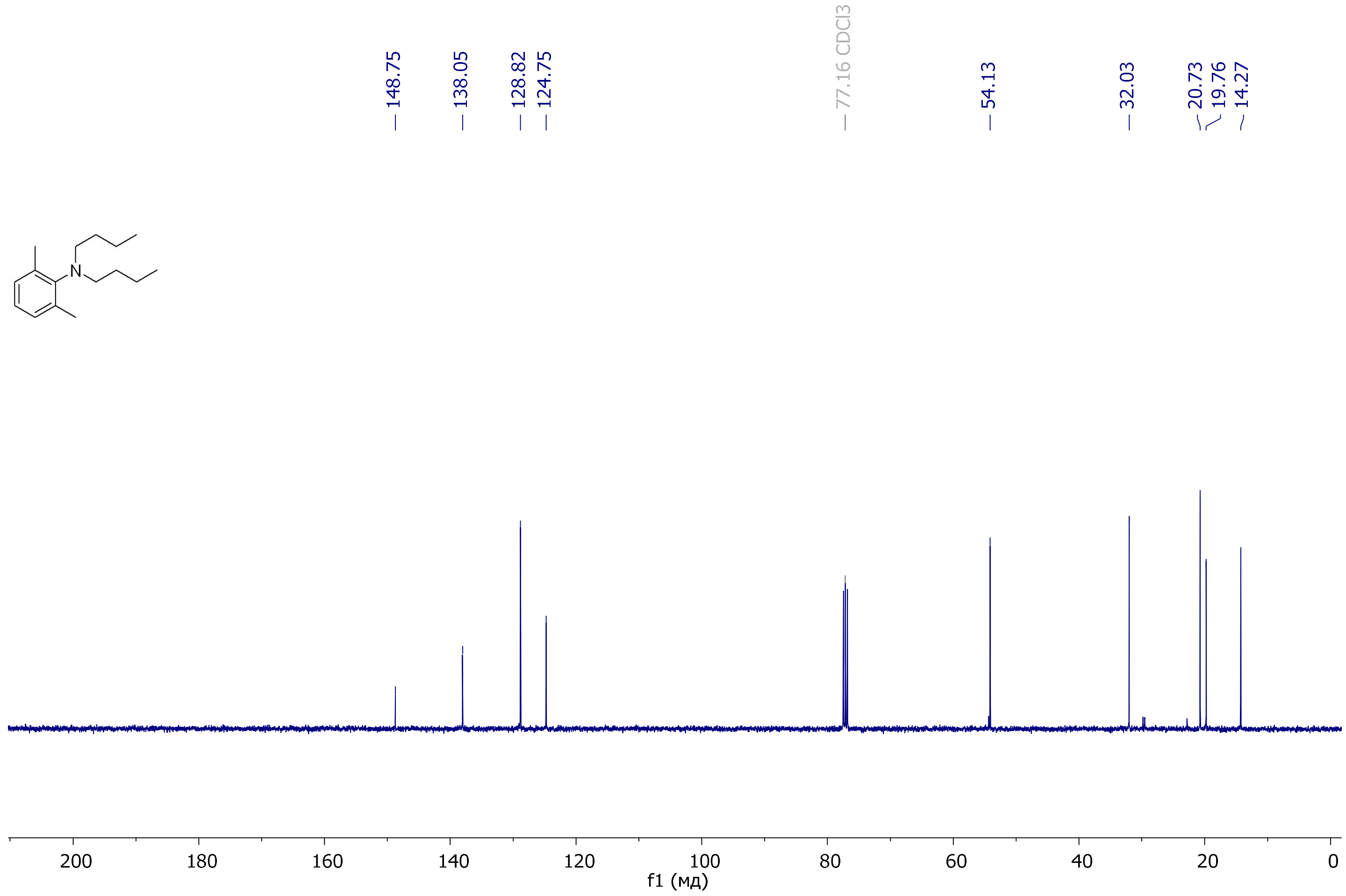

HRMS (ESI-TOF) of N,N-dibutyl-2,6-dimethylaniline (3n)

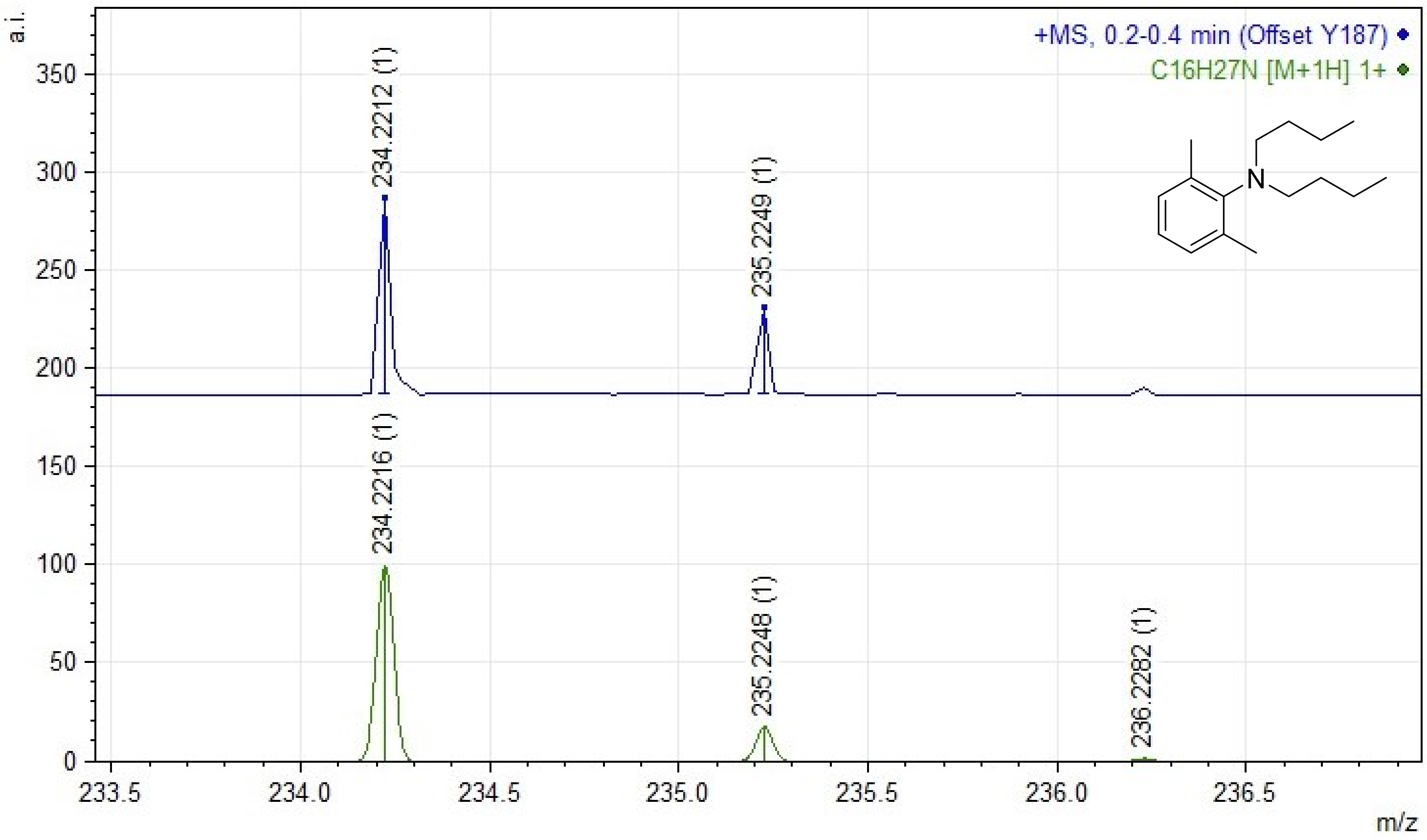

^{1}H NMR of N,N-dibutyl-2,6-diisopropylaniline (400 MHz, $CDCl_3$) (3o)

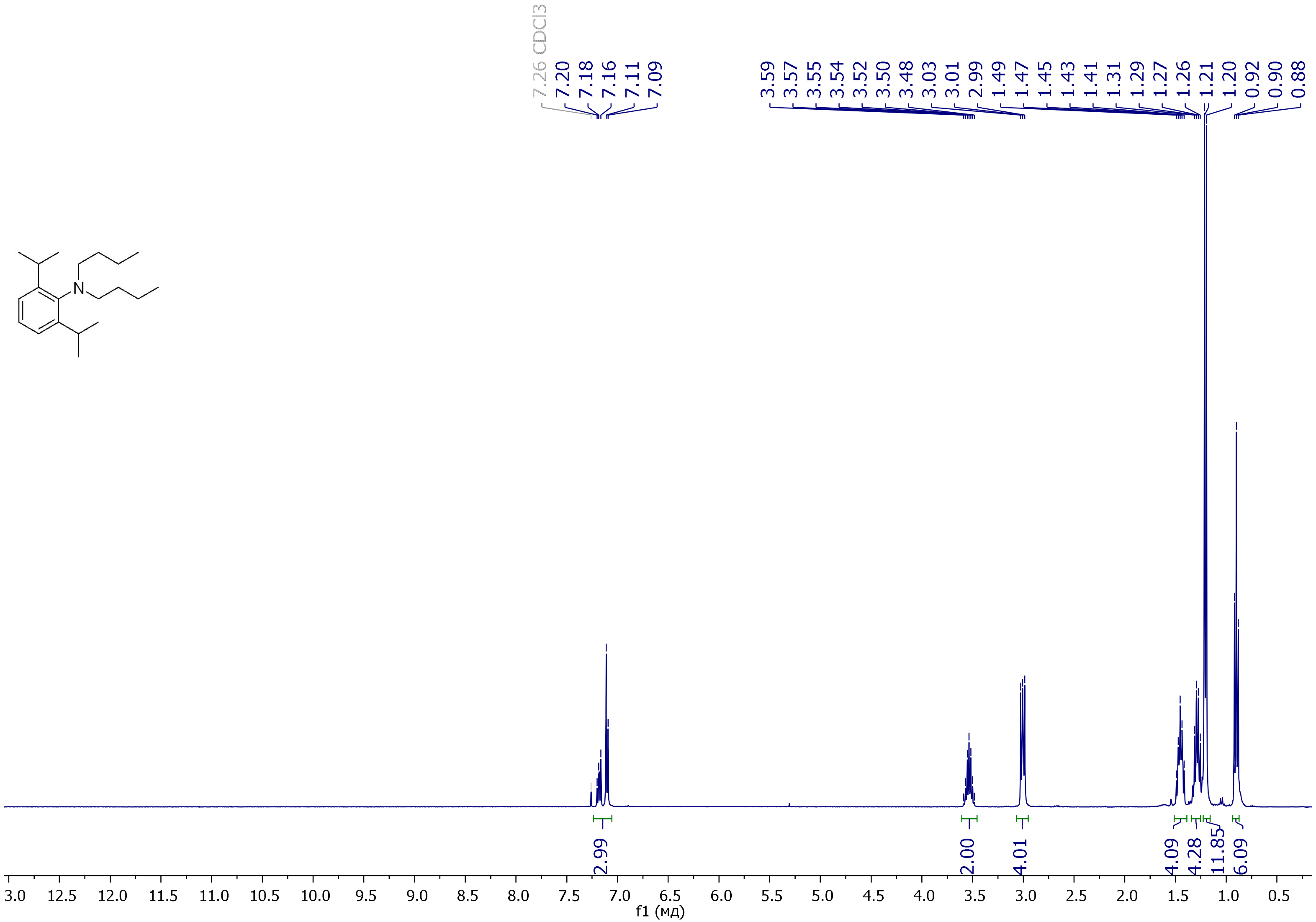

$^{13}C\{^{1}H\}$ NMR of N,N-dibutyl-2,6-diisopropylaniline (101 MHz, $CDCl_3$) (3o)

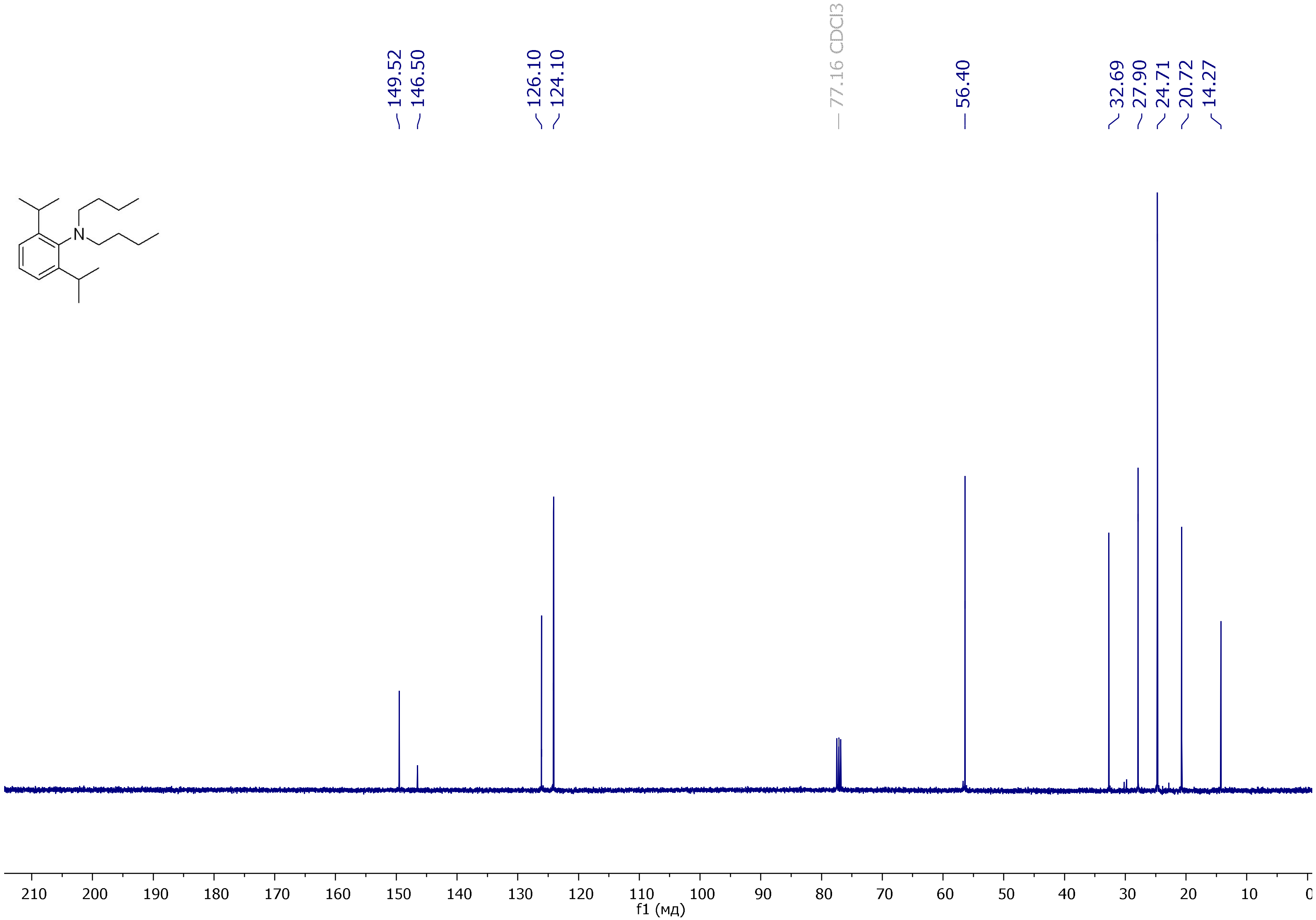

HRMS (ESI-TOF) of N,N-dibutyl-2,6-diisopropylaniline (3o)

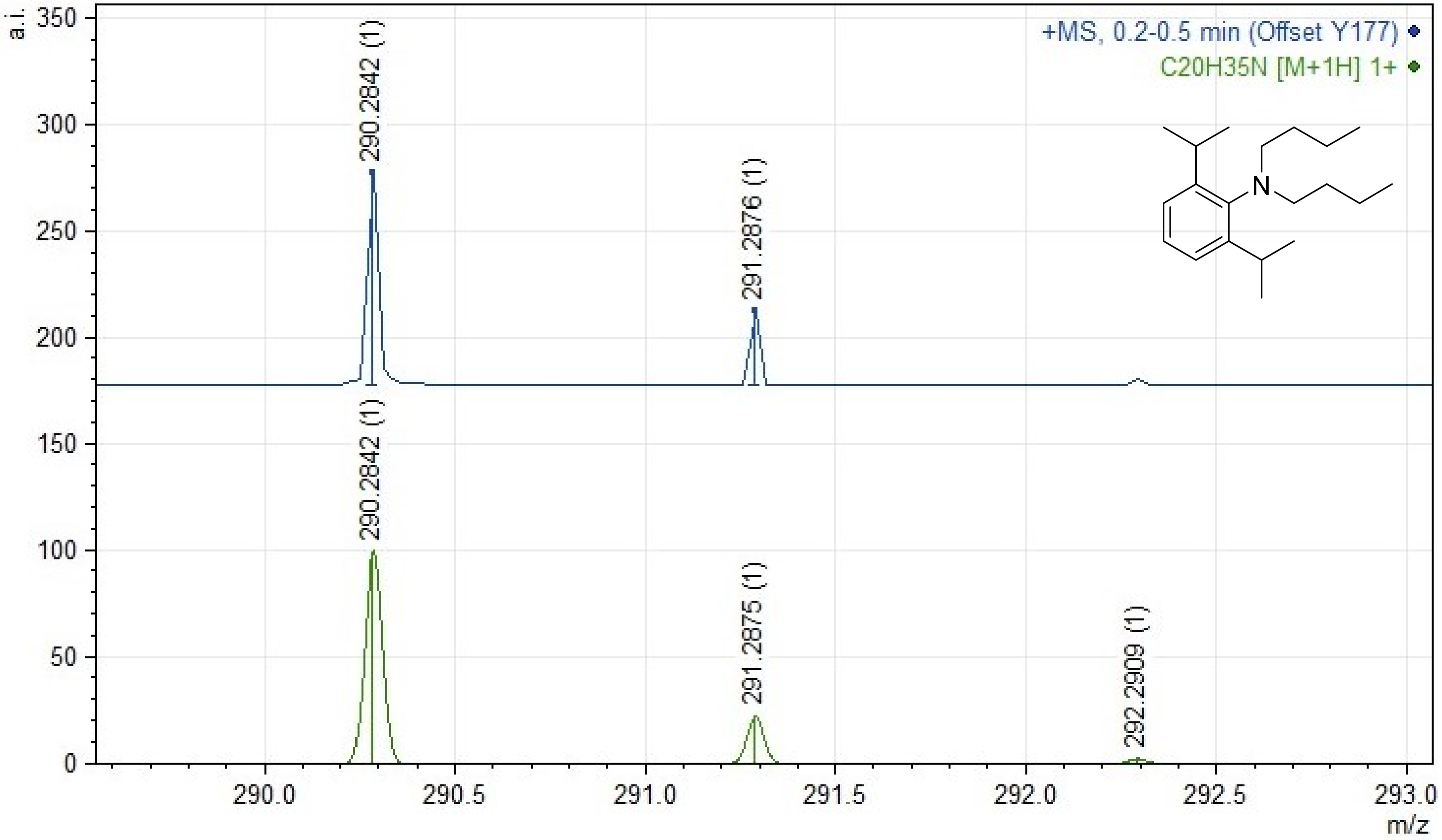

^{1}H NMR of ethyl 4-(dibutylamino)benzoate (400 MHz, $CDCl_3$) (3p)

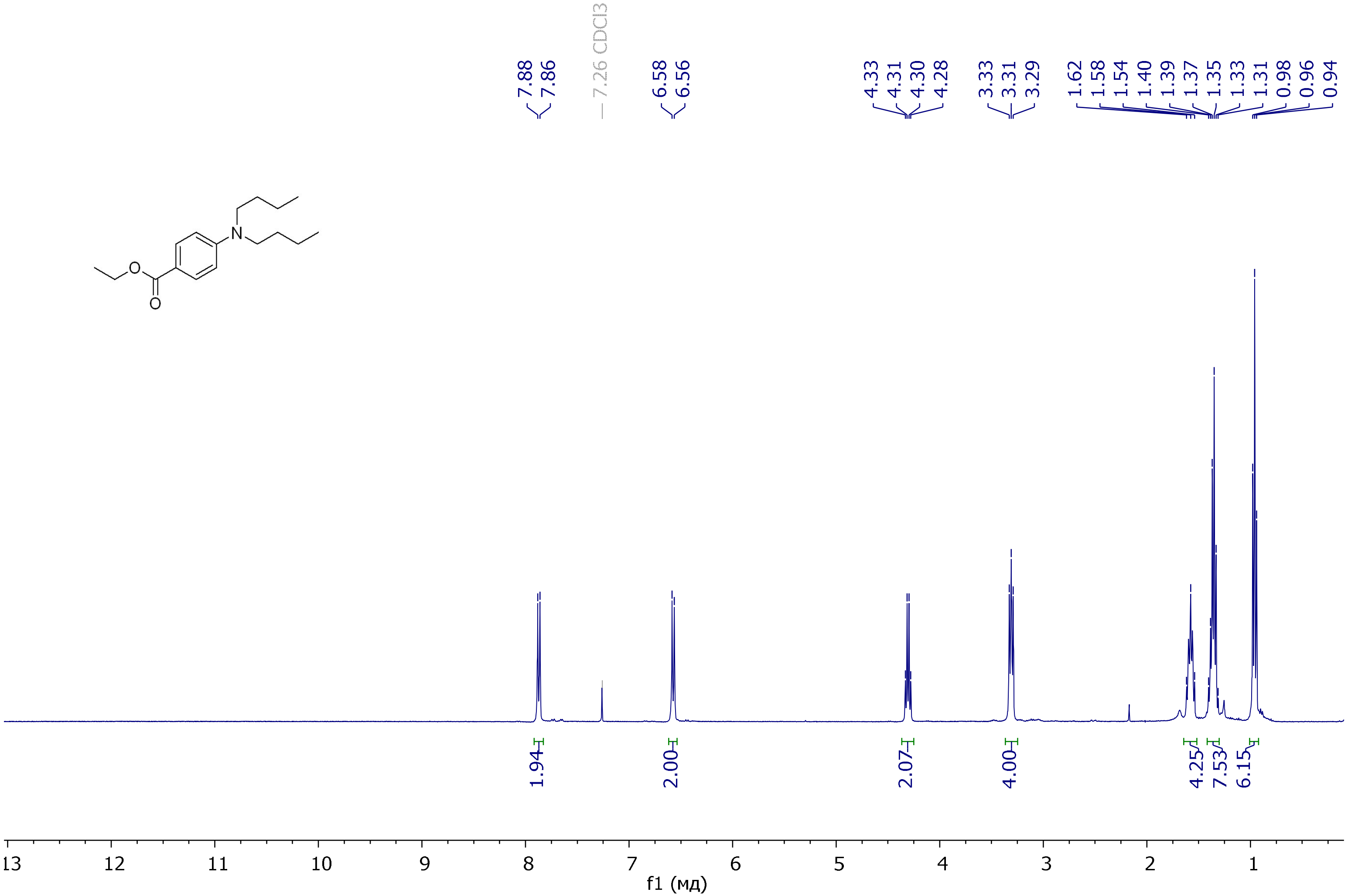

$^{13}C\{^1H\}$ NMR of ethyl 4-(dibutylamino)benzoate (101 MHz, $CDCl_3$) (3p)

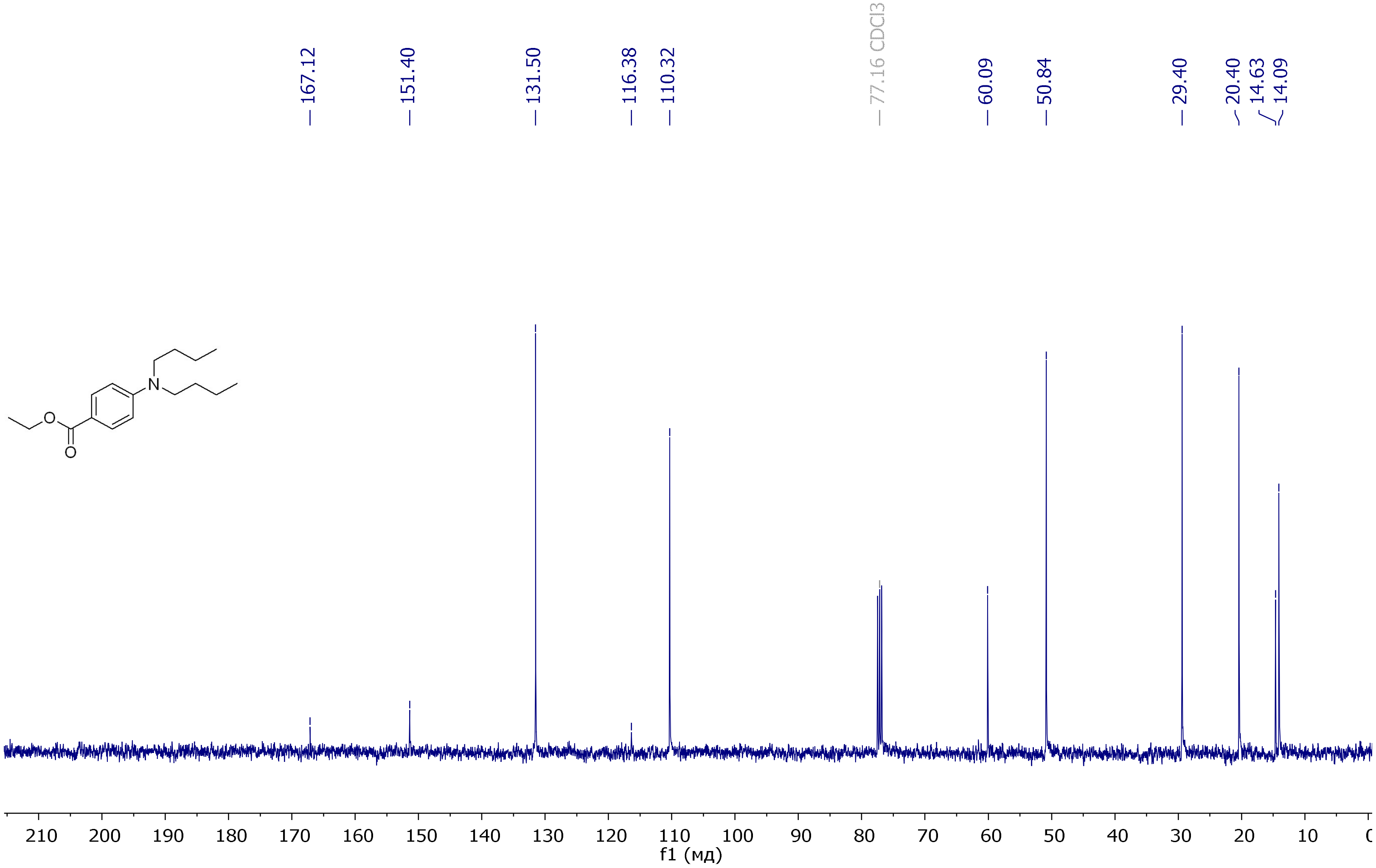

^{1}H NMR of 4-methoxy-N-pentyl-anilines mixture (400 MHz, $CDCl_3$)

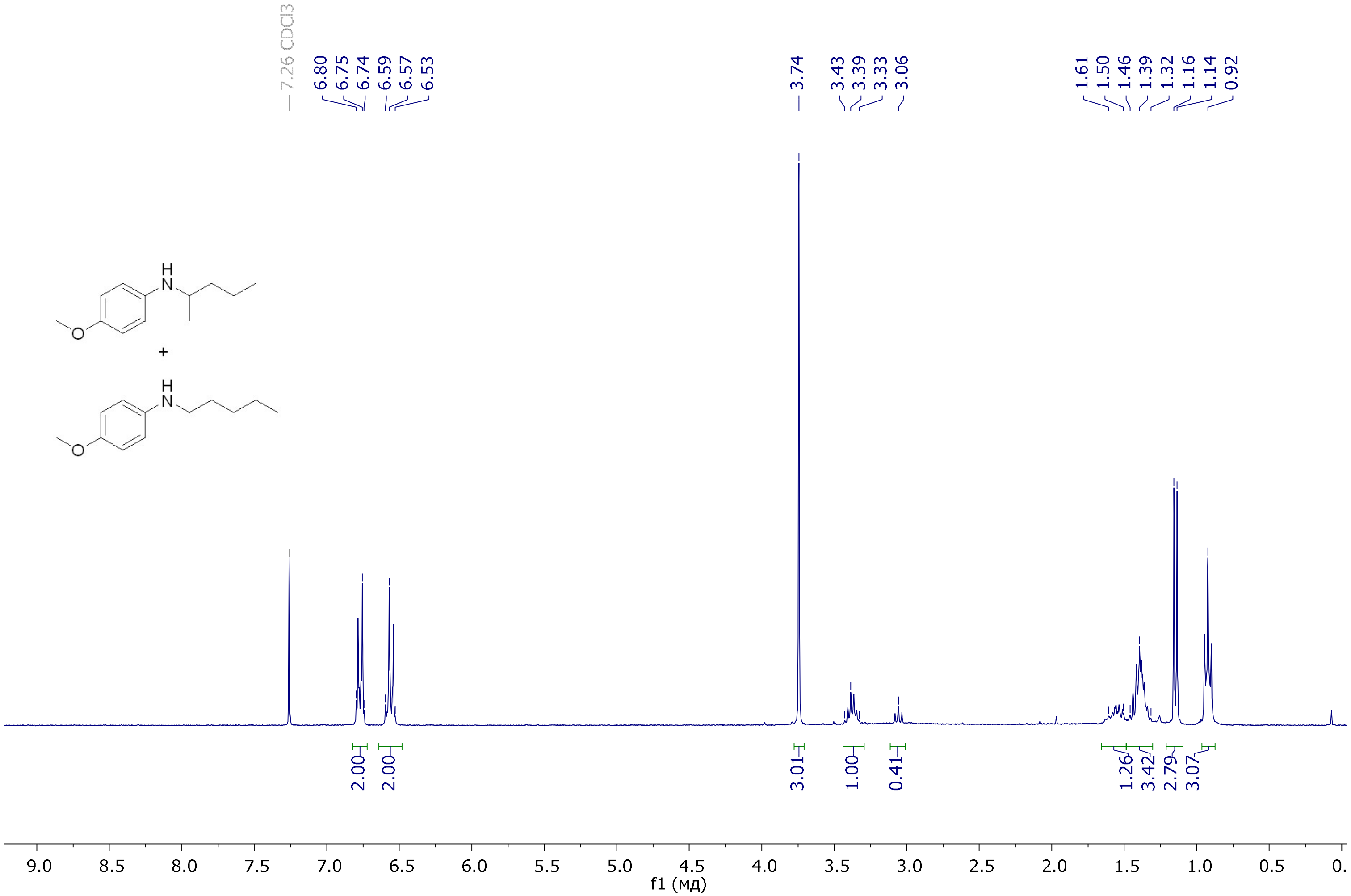

$^{13}C\{^1H\}$ NMR of 4-methoxy-N-pentyl-anilines mixture (101 MHz, $CDCl_3$)

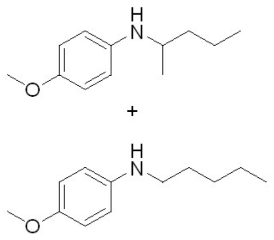

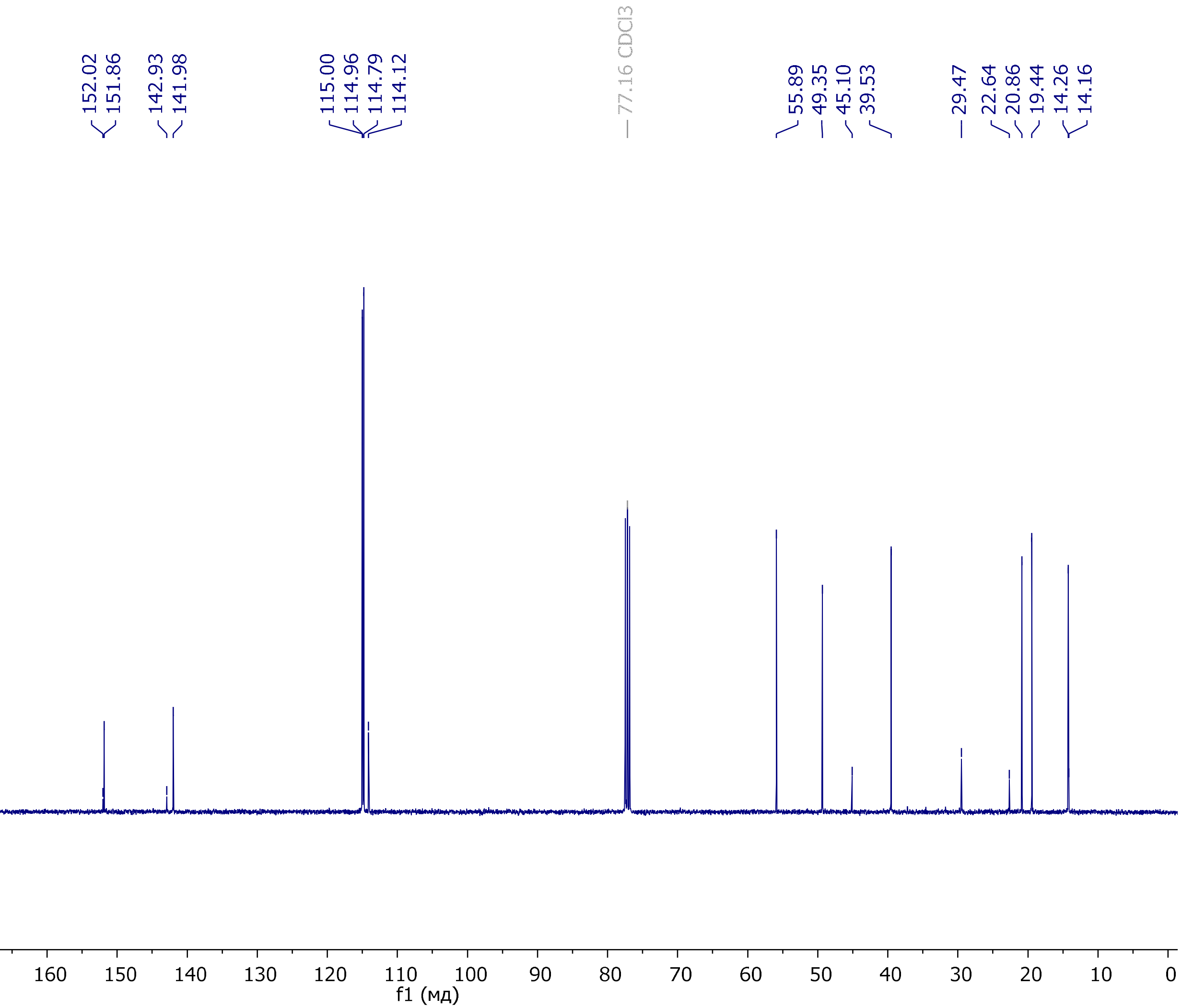

^{1}H NMR of N-(but-3-en-1-yl)-4-methoxyaniline (400 MHz, $CDCl_3$)

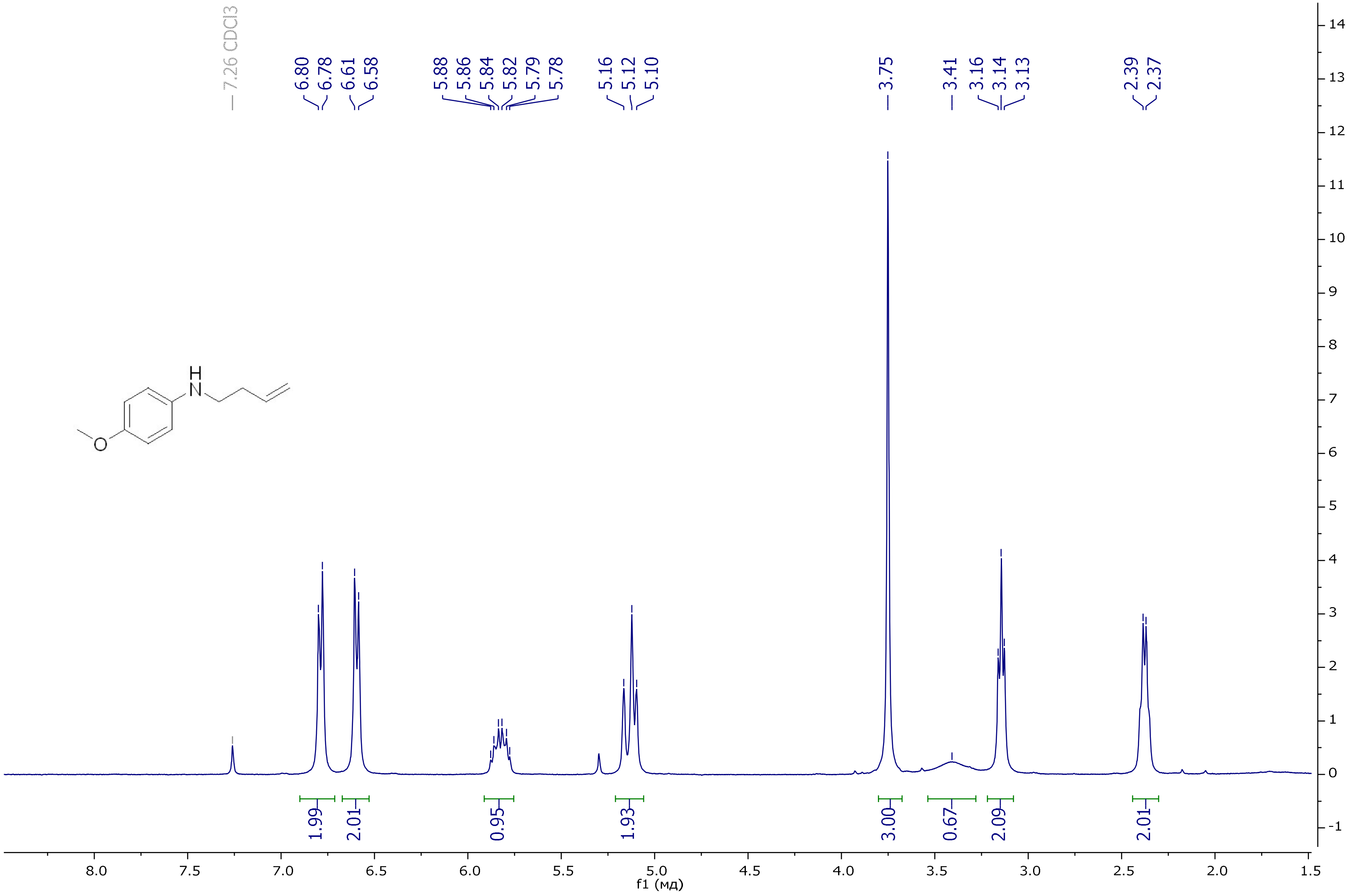

^1H NMR of 4-((4-methoxyphenyl)amino)butan-1-ol (300 MHz, $CDCl_3$)

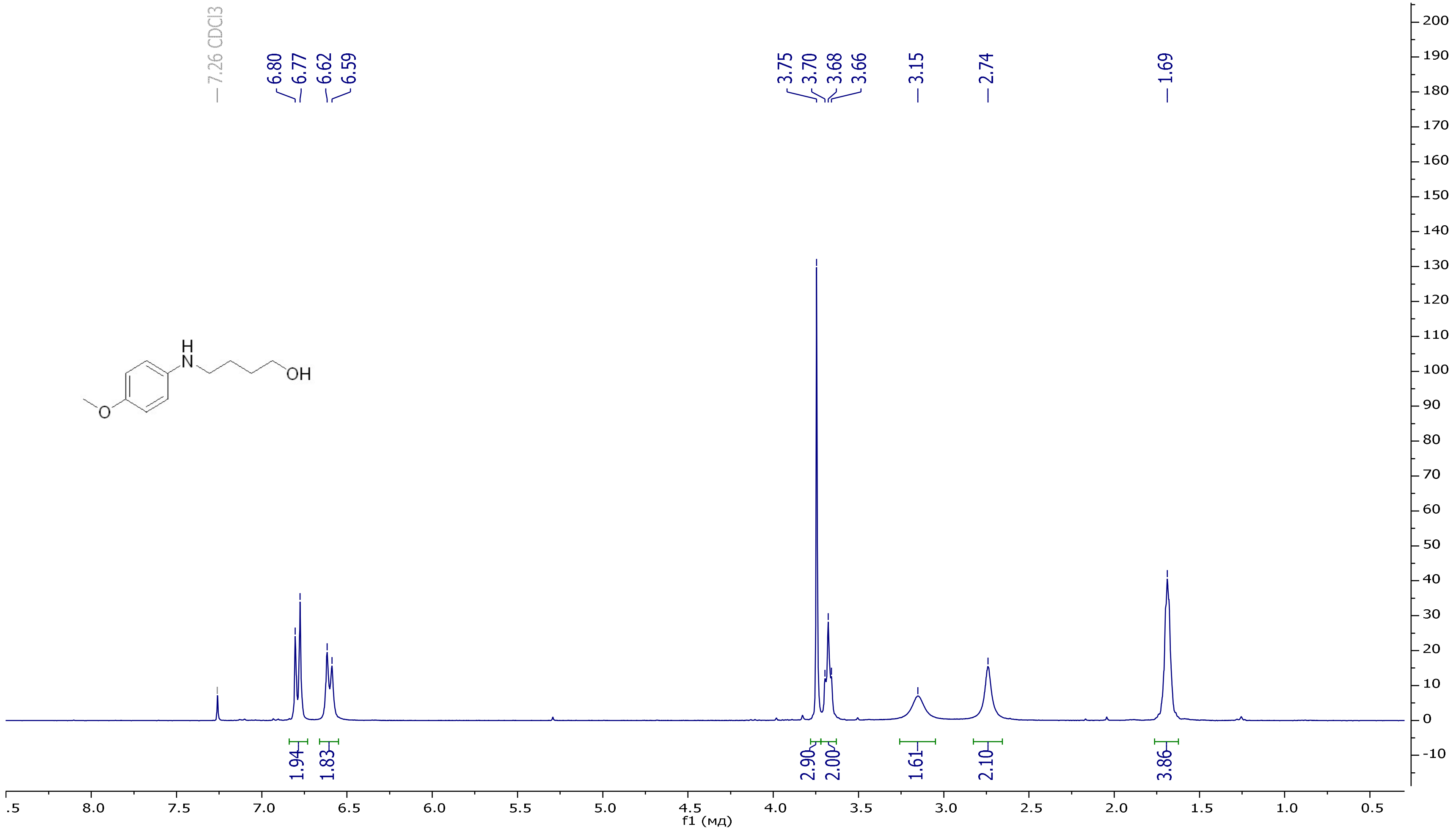

^{1}H NMR of 5-(butyl(4-methoxyphenyl)amino)pentan-1-ol (400 MHz, $CDCl_3$)

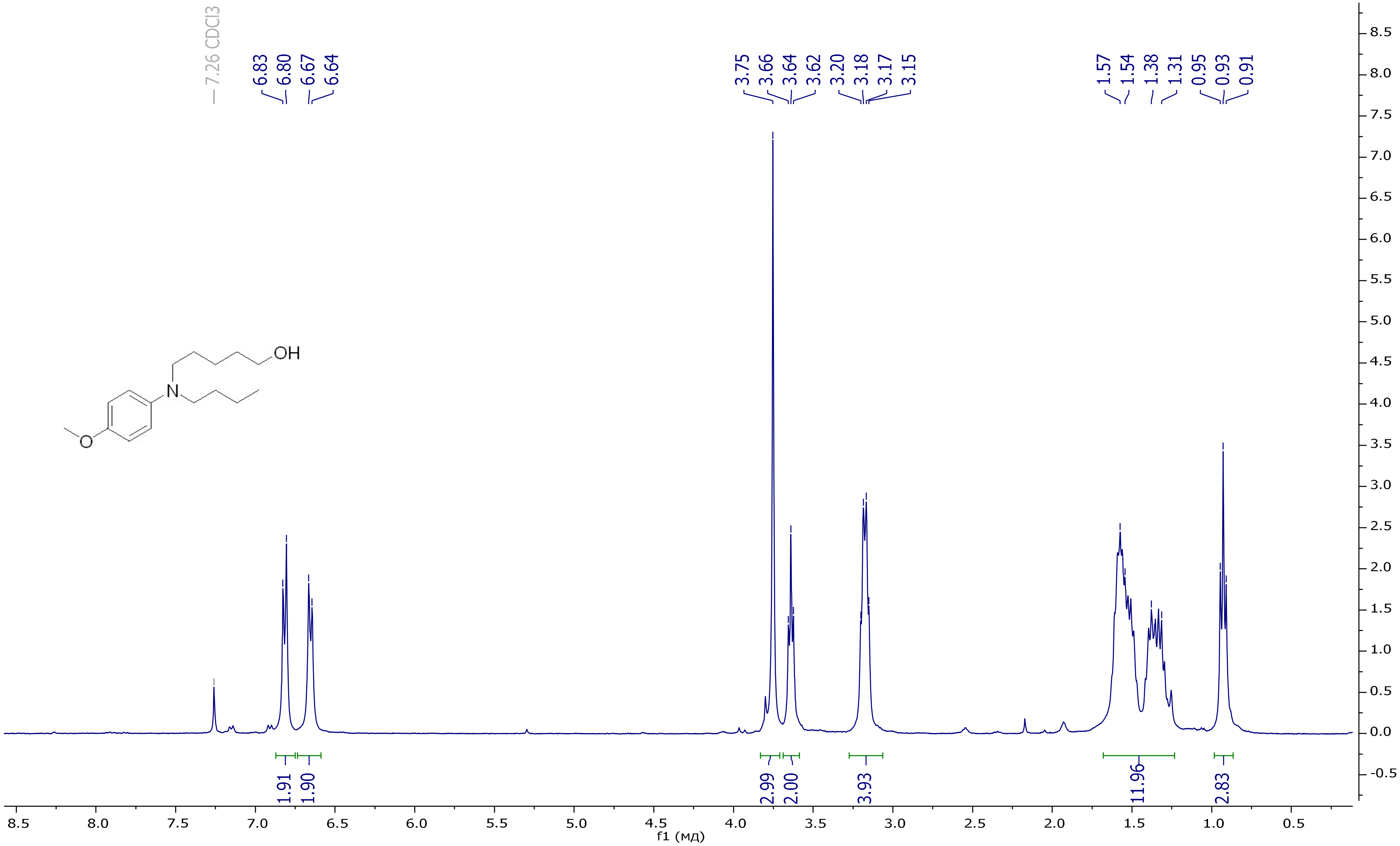

$^{13}C\{^1H\}$ NMR of 5-(butyl(4-methoxyphenyl)amino)pentan-1-ol (101 MHz, $CDCl_3$)

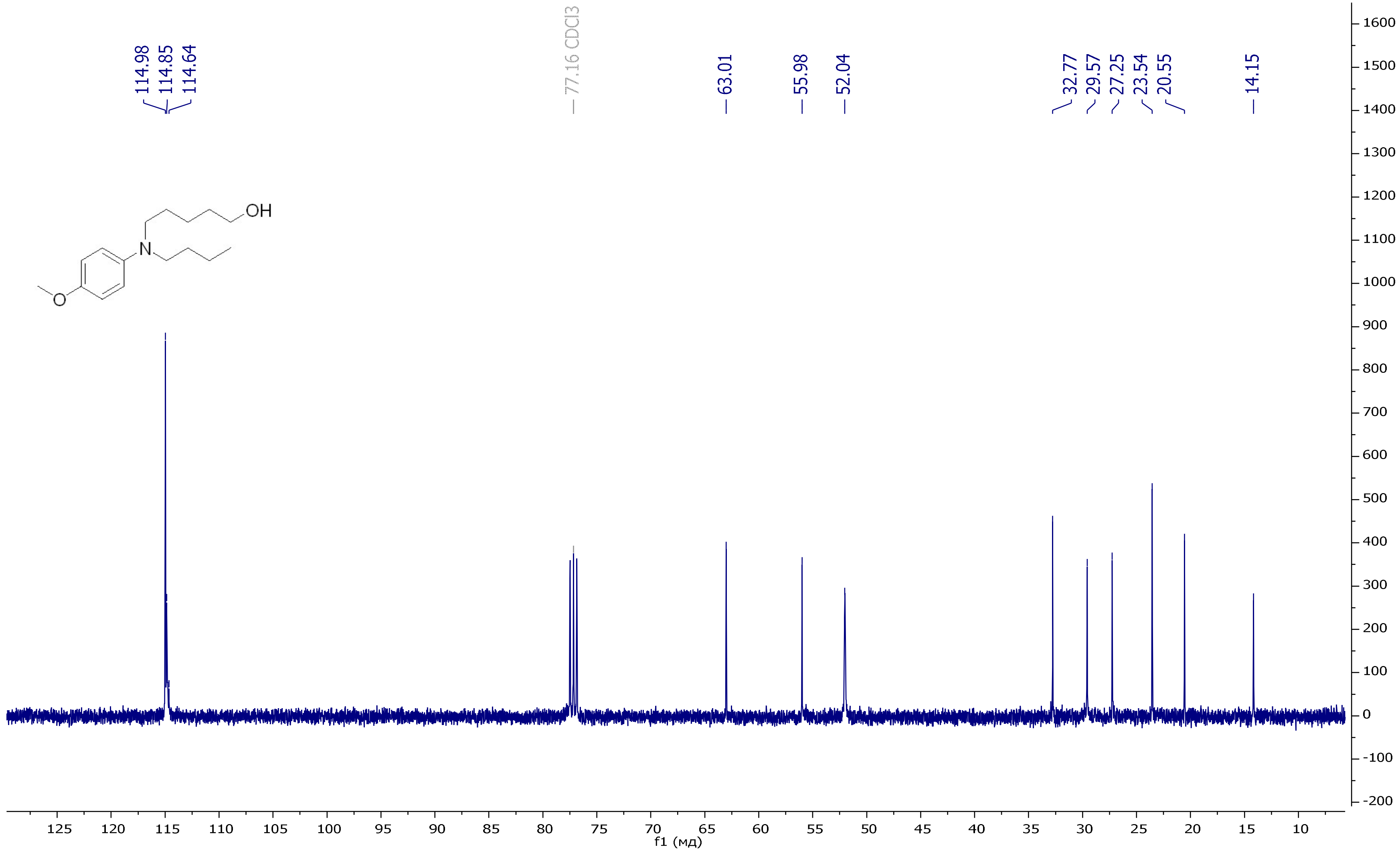

^{1}H NMR of 5-(butyl(4-methoxyphenyl)amino)pentan-1-ol (400 MHz, DMSO-d_6)

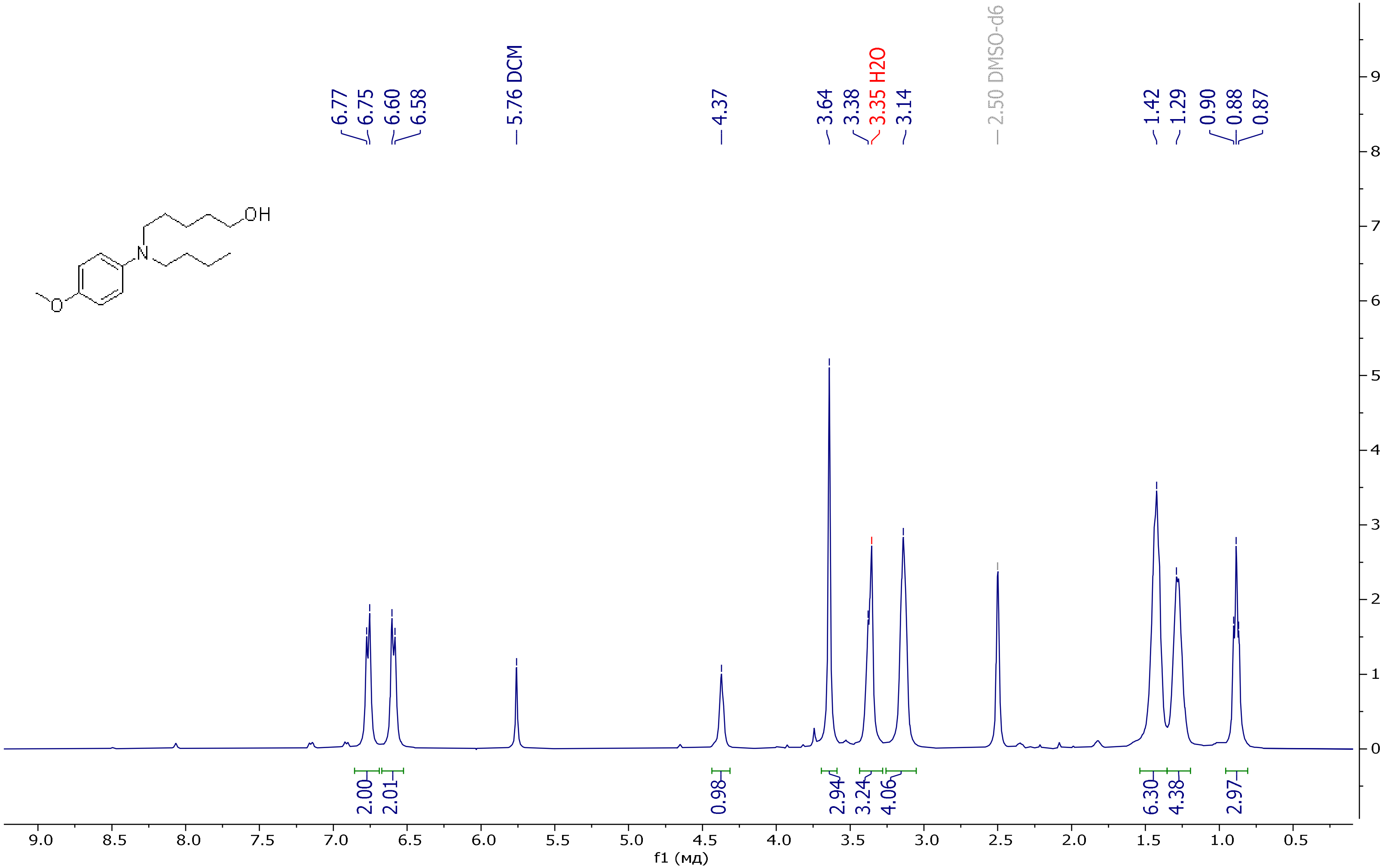

$^{13}C\{^1H\}$ NMR of 5-(butyl(4-methoxyphenyl)amino)pentan-1-ol (101 MHz, DMSO-d_6)

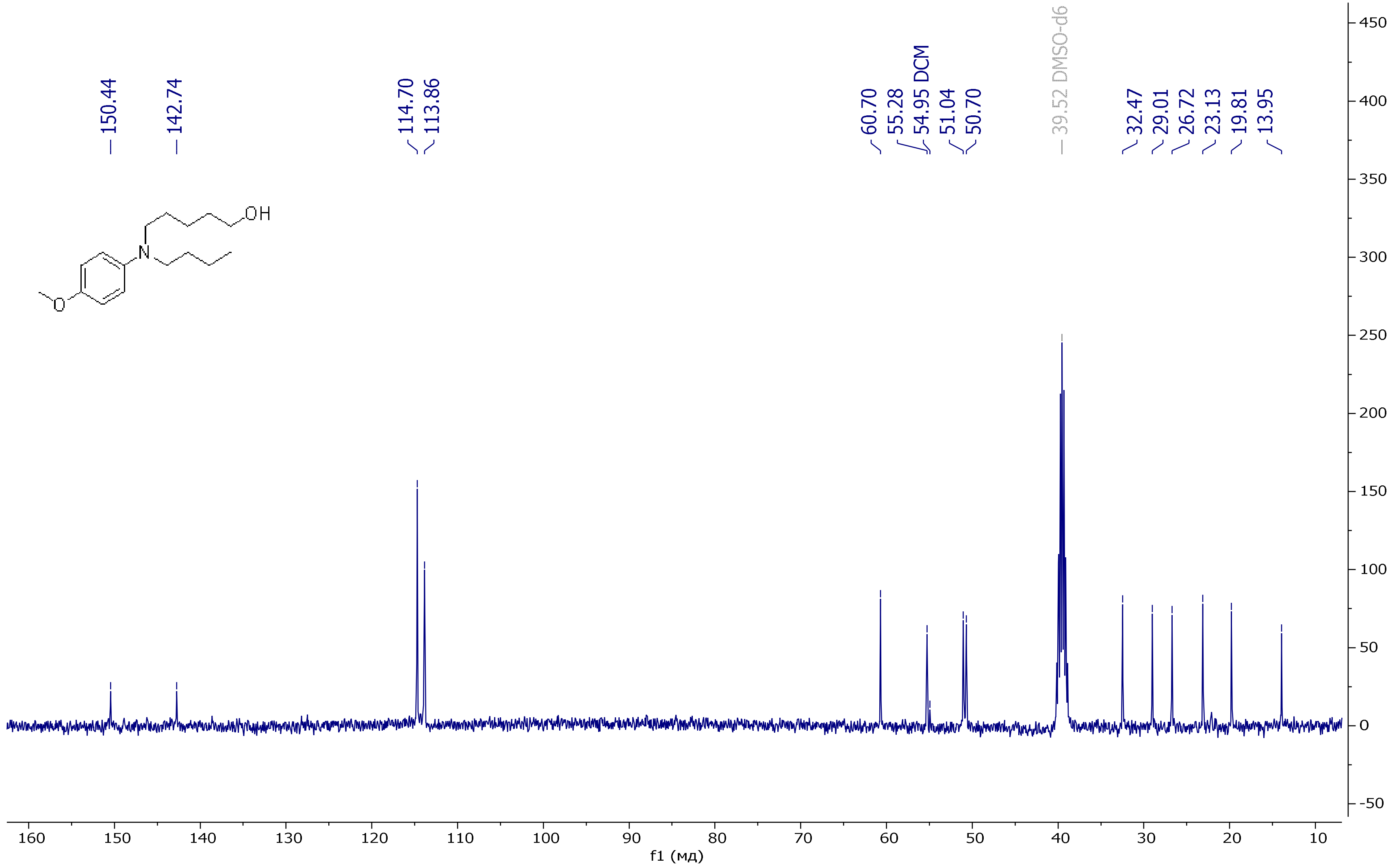

Supplement: Supplementary file 1 — Supplementary Material [file CSSC-18-e202402622-s001.pdf]
